# Cognitive appeal promotes the persistence of inefficient solutions and hinders cumulative cultural evolution

**DOI:** 10.1038/s41467-026-77677-9

**Published:** 2026-09-12

**Authors:** Ali Seyhun Saral, Manvir Singh, Antoine Jacquet, Haneul Jang, Maxime Derex

**Affiliations:** 1 Institute for Advanced Study in Toulouse, Toulouse, France; 2 University of California, Davis, CA USA; 3 Sciences Po, Paris, France; 4 CNRS, Toulouse School of Economics, Toulouse, France

**Keywords:** Human behaviour, Cultural evolution

## Abstract

Through cumulative cultural evolution, human populations discover novel solutions that allow them to solve complex problems. However, populations also reliably develop and sustain practices that are costly yet appear to have no effect on their stated outcome, such as bloodletting, divination, and malicious sorcery. Here, we show that ineffective, costly solutions can develop and be maintained because of their cognitive appeal. In a pre-registered transmission-chain experiment, participants were tasked with developing a solution to a problem over several trials, after which they passed instructions onto the subsequent generation. The visual appearance of the experimental task was manipulated to vary the extent to which cognitive biases could interfere with participants’ expectations of efficacy, enabling a direct test of the effect of cognitive appeal on cumulative cultural evolution. Participants in the low-appeal treatment (*n* = 240) progressively develop more efficient solutions across generations. Participants in the high-appeal treatment (*n* = 240), in contrast, produce costlier solutions, and we did not observe a statistically significant increase in efficiency over the course of the experiment. These results suggest that, even under conditions favorable to cumulative cultural evolution, a form of cognitive appeal can alone prevent the process and promote the emergence and cultural persistence of ineffective solutions.

## Abstract (web summary)

Societies develop both effective practices and costly ineffective ones. Here, the authors show that opportunities for intuitive solutions can prevent cumulative refinement and promote the persistence of ineffective practices across generations.

## Introduction

Cumulative cultural evolution appears critical for humans’ success^1–3^. Through the accumulation of cultural innovations over many generations, populations have developed solutions that no single individual could have invented, allowing us to inhabit new environments and achieve feats unparalleled in the animal world, from space exploration to industrial-scale agriculture. Several experiments have shown that cumulative cultural evolution occurs through instrumental, payoff-biased social learning, which allows the gradual accumulation of beneficial modifications over time^4–6^. Participants tasked with solving a problem, such as building a basket to carry rice or designing a wheel to roll down a track, preferentially adopt and retain solutions that yield greater expected payoffs^7–9^. When successive generations of learners select both successful demonstrators and effective-seeming solutions, cumulative cultural evolution can drive the emergence of complex, highly efficient solutions^6,8^.

Theoretical and empirical work has shown that cumulative cultural evolution operates under a wide range of conditions, albeit with differing levels of effectiveness. When payoffs are immediate and accurate, payoff-biased social learning allows individuals to filter out less efficient solutions, gradually bringing populations closer to the optimal solution over time^10,11^. However, when payoffs are noisy or delayed, gradual improvements are slower, as individuals struggle to accurately assess the effectiveness of solutions^12^. Additionally, experimental work has demonstrated that populations tend to converge on less efficient solutions in environments that are misaligned with their prior assumptions^13^. These mitigating factors notwithstanding, cumulative cultural evolution remains powerful enough to foster the emergence of increasingly efficient solutions across diverse conditions, even when those solutions are counterintuitive and beyond individual understanding^8,9^.

However, while cumulative cultural evolution reliably promotes the emergence of increasingly efficient solutions in experimental settings, it also appears to produce and preserve ineffective practices in the real world. Humans around the world engage in practices that seem to have no effect on their stated outcome, such as divination^14,15^, rain magic^16^, malicious sorcery^17^, and bloodletting^18^. These practices are widespread and elaborate, requiring non-trivial investments of time and resources. Research at the intersection of cognitive science and anthropology suggests that these practices may develop because of systematic biases in how individuals evaluate solutions. These biases are examples of “content biases” that can influence cultural evolution^19^. For example, prior research showed that participants in the United States and Brazil evaluated rituals as more effective when they involved features like repetition or specific times, despite the rituals having no effect on their intended outcome^20^.

Of the features that can bias evaluations of potential solutions, one of the most widespread seems to be a visual correspondence between an intervention and its target. Anthropologists have long observed that people often use magic and folk medicines that visually resemble the desired outcome^21^. Well-known examples include voodoo dolls or effigies, in which a practitioner tries affecting another person by manipulating an object that resembles them, as well as the doctrine of signatures, which holds that herbs resembling specific body parts can be used to treat ailments affecting those parts^22,23^. Such correspondences have been hypothesized to tap into widespread cognitive biases^24,25^.

Here, we test whether subjective appeal, in the form of visual similarity between an intervention and its target, can drive the emergence and persistence of ineffective solutions. Using a preregistered transmission-chain experiment in which successive participants repeatedly improve solutions to a complex task, we manipulate whether solutions can visually resemble their targets without changing the underlying payoff structure. We find that subjective appeal promotes the emergence and cultural persistence of ineffective solutions, revealing how content biases can undermine cumulative cultural evolution.

## Results

**Fig. 1:**
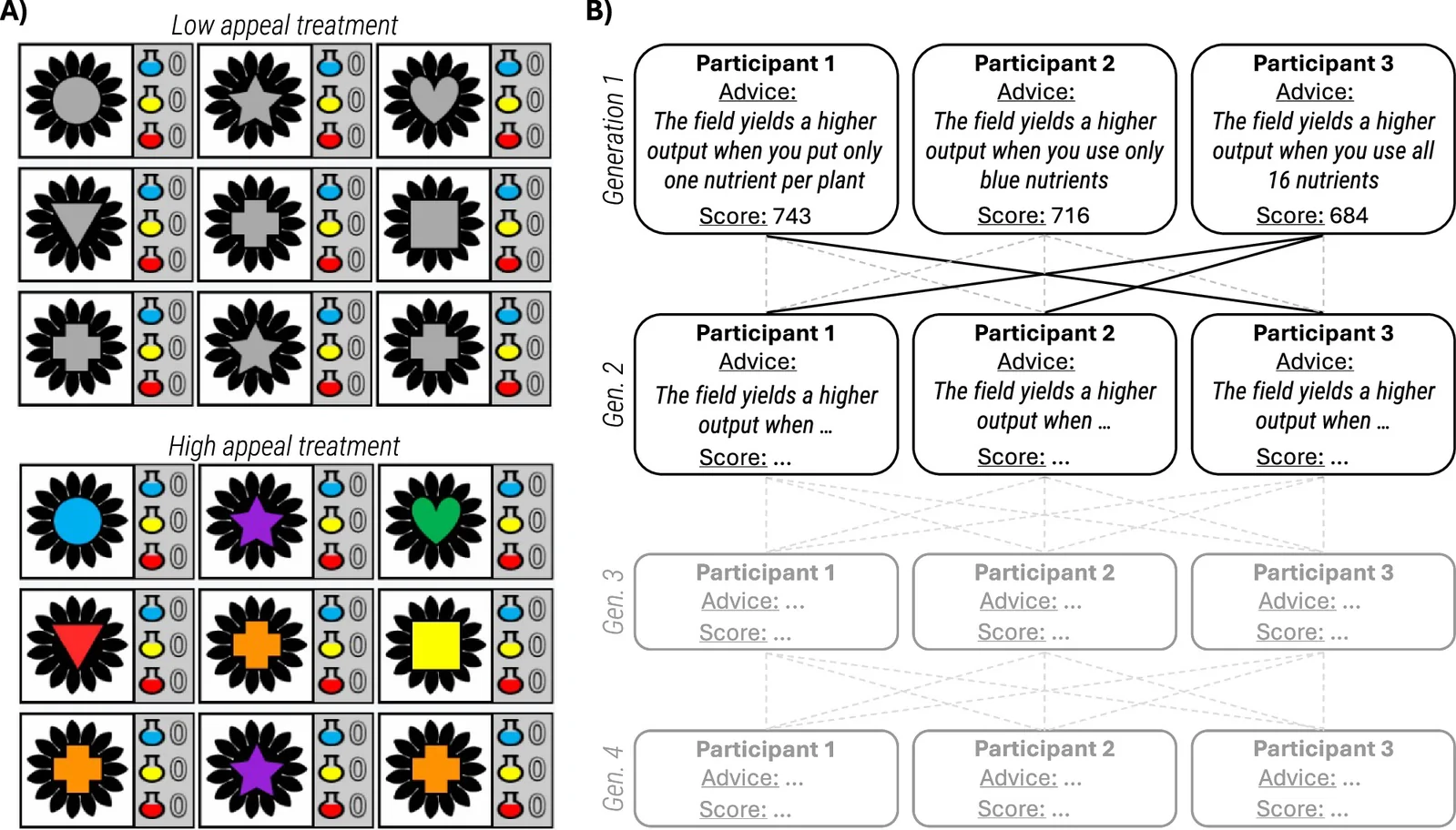
Problem-solving task and experimental design. **A** Plant-feeding task: participants were presented with six uniquely shaped flowers within a field of nine plants. The plants appeared either uniformly grey (low-appeal treatment) or in various colors, with each shape paired to a specific color (high-appeal treatment). Participants had access to three different nutrient types and could use up to 16 nutrients across the entire field. After each trial, participants received feedback on the score generated by their solution. **B** Participants were organized into experimental chains consisting of four generations, with three participants per generation. After engaging with the task for six trials, each participant wrote a piece of advice for participants in the subsequent generation. From Generation 2 onward, participants could select one cultural demonstrator based on that individual’s written advice and final-trial score before solving the task and writing their own piece of advice for the next generation.

To test whether subjective appeal can interfere with cumulative cultural evolution, we conducted an experiment in which participants were tasked with maximizing their score by allocating nutrients to nine plants in a virtual field (Fig. 1A). Nutrients came in three colors of different costs—blue (2 units), yellow (6 units), and red (10 units)—and participants could allocate up to sixteen nutrients across the nine plants. This resulted in a vast solution space, with billions of distinct configurations. The field’s output varied as a function of how nutrients were allocated across plants, although scores were noisy through the inclusion of a random component (see “Methods” section). The optimal solution was to provide each plant with a single nutrient of the lowest cost (the blue nutrient).

Participants were organized into experimental chains of four generations, with each generation consisting of three participants (Fig. 1B). Each participant completed six trials and received feedback on their score after each one. After their final trial, participants wrote a piece of advice for the next generation. From the second generation onward, participants were shown the final scores and written advice of three individuals from the previous generation and were asked to choose one as their cultural demonstrator before beginning their first trial.

We implemented two experimental treatments that differed in the degree of visual similarity between the features of the plants and those of the nutrients. In the low-appeal treatment (*n* = 240), the plants had different shapes but were all grey in color (Fig. 1A). In the high-appeal treatment (*n* = 240), in contrast, plants were additionally assigned one of six colors: plant colors were either primary colors, matching the colors of the nutrients (blue, yellow, and red), or secondary colors that can typically be created by mixing the primary colors (green, purple, and orange). Thus, in this treatment, the nutrients fed to the plants could resemble the plants themselves, potentially allowing for more visually appealing solutions than in the low-appeal treatment. Importantly, the size of the solution space was kept equivalent across experimental treatments by pairing each plant shape with a unique color (Fig. 1A).

Under these conditions, rates of cumulative cultural evolution should be comparable across treatments if cognitive biases do not influence participants’ expectations of efficacy. Conversely, if changes in the visual appearance of stimuli interfere with these expectations, we would expect rates of cumulative cultural evolution to differ between treatments. To test this, we compared the score and cost of culturally transmitted solutions—that is, the solutions produced on the sixth trial—across generations in the low-appeal and high-appeal treatments. We predicted that the score of transmitted solutions would increase over generations in the low-appeal treatment and that this increase would exceed any change observed in the high-appeal treatment (see Pre-registration section). Additionally, we predicted that participants in the low-appeal treatment would rely on payoff information to a greater extent when selecting their cultural demonstrator than participants in the high-appeal treatment.

**Fig. 2:**
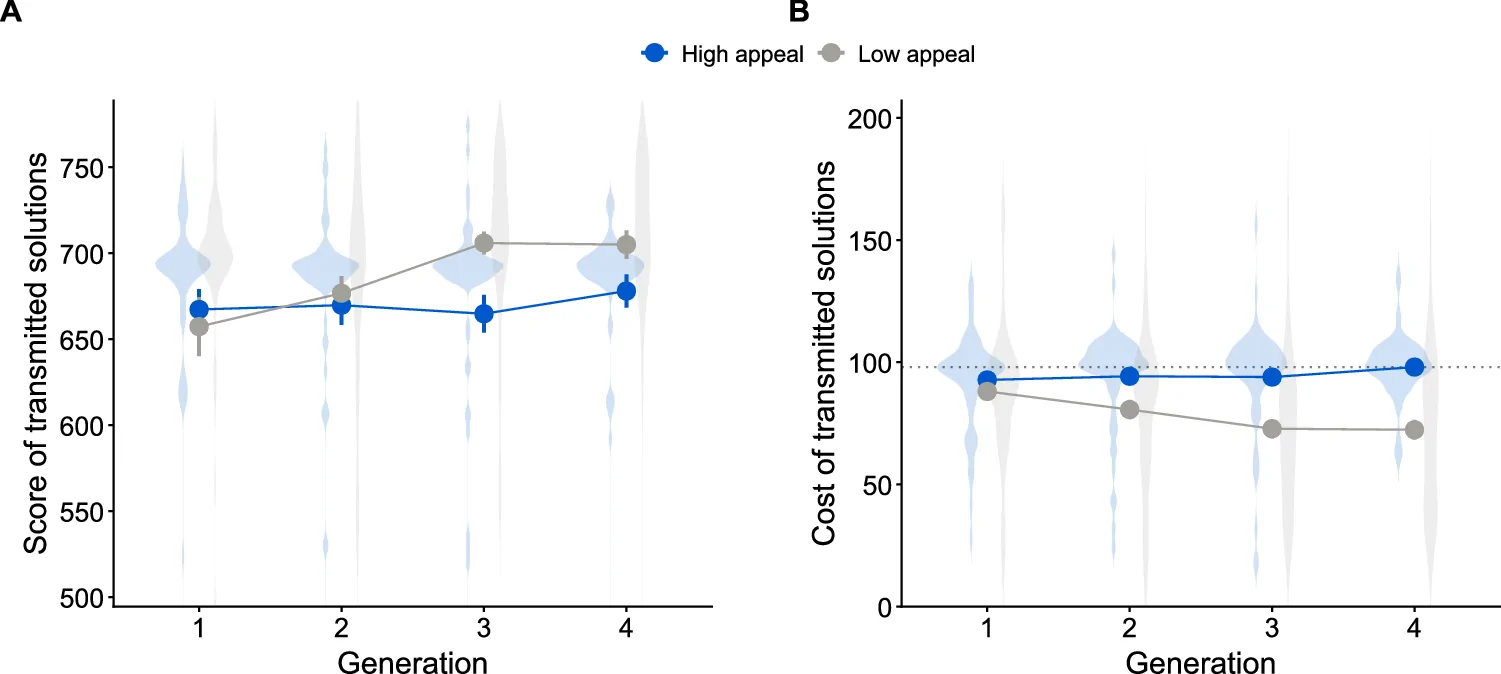
Score and cost of culturally transmitted solutions across generations. **A** In the low-appeal treatment (grey), scores increased across generations, whereas no statistically significant change was observed in the high-appeal treatment (blue). **B** In the low-appeal treatment, solution costs decreased across generations, whereas no statistically significant change was observed in the high-appeal treatment. The dotted line represents the cost of the mix-and-match solution (see Fig. 5 for an illustration). Total sample size: *N* = 480, with *n* = 60 observations contributing to each mean and violin plot. Data are presented as mean values, and error bars indicate ±1 standard error of the mean (SEM).

In the low-appeal treatment, the score of the transmitted solutions increased over the four generations (Fig. 2A), while their cost progressively decreased (Fig. 2B, see “Methods” for details on score and cost calculation). In contrast, in the high-appeal treatment neither the score (Fig. 2A) nor the cost (Fig. 2B) changed significantly across generations. Scores in the low-appeal treatment increased by an estimated 17.23 points with each additional generation (*t*(220.00) = 3.49, *p* < 0.001, *b* = 17.23, 95% CI = [7.55, 26.90]), whereas no statistically significant change was observed in the high-appeal treatment across generations (*t*(220.00) = 0.56, *p* = 0.575, *b* = 2.72, 95% CI = [−6.77, 12.21]; see Table 1 for details).

**Table 1:** Linear mixed-effect regression model estimates for the score and cost of culturally transmitted solutions across treatments

Term	Estimate (*b*)	SE	95% CI	d*f*	*t*	*p*
**Score**						
Low-appeal only (*n* = 240, num. chains = 20)						
(Intercept)	660.39	9.71	[641.37, 679.42]	97.32	68.04	<0.001
Generation	17.23	4.94	[7.55, 26.90]	220.00	3.49	<0.001
High-appeal only (*n* = 240, num. chains = 20)						
(Intercept)	665.87	9.42	[647.40, 684.34]	101.47	70.68	<0.001
Generation	2.72	4.84	[−6.77, 12.21]	220.00	0.56	0.575
Pooled (*n* = 480, num. chains = 40)						
(Intercept)	665.87	9.56	[647.12, 684.62]	198.61	69.62	<0.001
Low-appeal	−5.48	13.53	[−31.99, 21.03]	198.61	−0.40	0.686
Generation	2.72	4.89	[−6.86, 12.30]	440.00	0.56	0.578
Low-appeal:generation	14.51	6.91	[0.96, 28.06]	440.00	2.10	0.036
**Cost**						
Low-appeal only (*n* = 240, num. chains = 20)						
(Intercept)	86.67	4.40	[78.04, 95.31]	36.11	19.68	<0.001
Generation	−5.49	1.50	[−8.43, −2.56]	220.00	−3.67	<0.001
High-appeal only (*n* = 240, num. chains = 20)						
(Intercept)	92.40	2.11	[88.26, 96.53]	114.70	43.80	<0.001
Generation	1.55	1.11	[−0.64, 3.73]	220.00	1.39	0.167
Pooled (*n* = 480, num. chains = 40)						
(Intercept)	92.40	3.45	[85.63, 99.17]	86.84	26.76	<0.001
Low-appeal	−5.72	4.88	[−15.30, 3.85]	86.84	−1.17	0.244
Generation	1.55	1.32	[−1.04, 4.13]	440.00	1.17	0.242
Low-appeal:generation	−7.04	1.87	[−10.70, −3.38]	440.00	−3.77	<0.001

Separate models are reported for the low-appeal condition, the high-appeal condition, and the pooled data. All models included chain as a random intercept. Fixed effects were evaluated using two-sided Wald t-tests with Satterthwaite approximation for degrees of freedom. Confidence intervals are Wald 95% intervals. Reference levels: Generation = 1, Treatment = High-appeal (pooled-model).

Among the three variables that affected solution scores (number of plants treated, number of nutrients used, and the type of nutrients used; see “Methods”), only the type of nutrients significantly differed between treatments, with the number of blue nutrients increasing across generations in the low-appeal treatment (Generation × Low-appeal interaction: *t*(440.00) = 3.88, *p* < 0.001, *b* = 0.95, 95% CI = [0.47, 1.43]; see Supplementary Fig. 1 and Supplementary Table 1 for details).

Contrary to our second prediction, we observed no statistically significant differences between treatments in social learning strategies. Participants in both treatments relied on payoff information when choosing among the three available cultural models, selecting the highest-scoring demonstrators 72% of the time in the low-appeal treatment and 78% of the time in the high-appeal treatment. The mean agreement among learners, calculated as the pairwise proportion of learners within a chain who selected the same demonstrator, was also comparable between treatments (67.2% and 66.1%, respectively; see Supplementary Fig. 2), as was the probability of a participant implementing the inherited solution in their first trial (14.4% and 18.3%, respectively; see Supplementary Fig. 3A and Supplementary Table 2). However, the similarity between the solution participants inherited and the solution they used in their first trial was higher in the high-appeal treatment than in the low-appeal treatment overall (Low-appeal: *t*(105.75) = 2.54, *p* = 0.013, *b* = 3.71, 95% CI = [0.85, 6.58]; see Supplementary Fig. 3 Panel B and Supplementary Table 2 Section B for details).

**Fig. 3:**
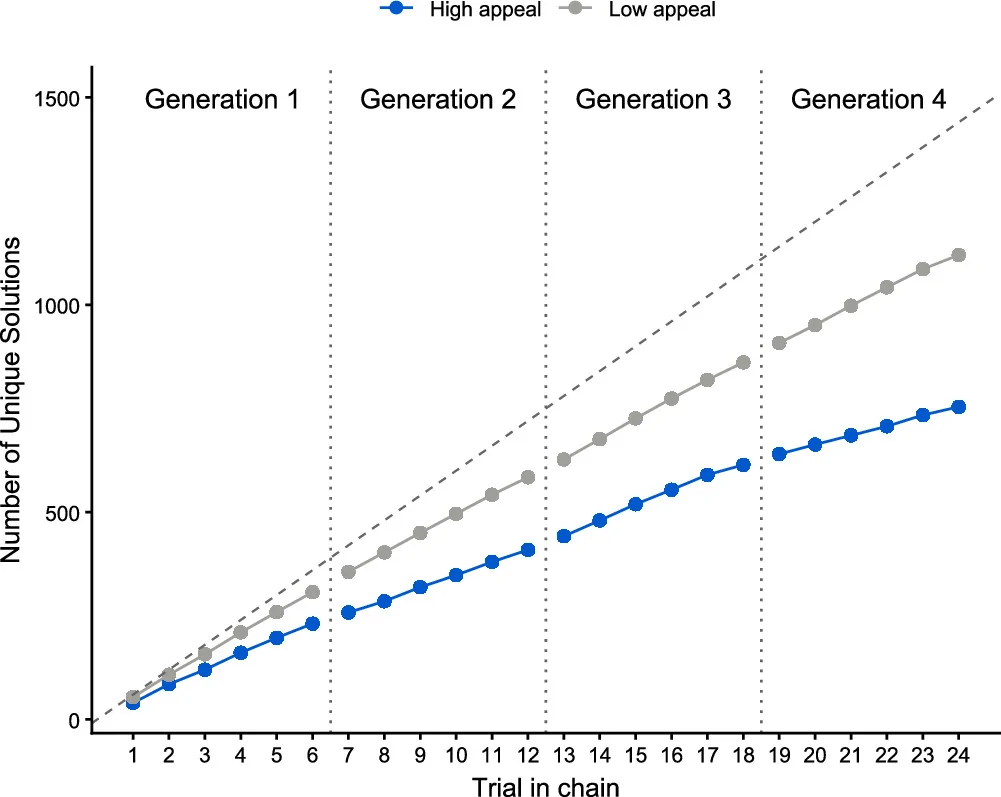
Exploration across trials. Cumulative number of unique solutions (i.e., nutrient-to-plant allocation patterns) across trials in each treatment. The dashed black line indicates the theoretical upper bound of exploration, assuming every participant submitted a solution not previously produced in this treatment. The grey line shows the cumulative number of unique solutions observed in the low-appeal treatment, and the blue line shows the high-appeal treatment.

To understand why cumulative performance differs between treatments despite similar social learning strategies, we conducted exploratory analyses comparing exploration and cultural transmission patterns. Results from these analyses show that participants explored the solution space more extensively in the low-appeal treatment than in the high-appeal treatment. On average, they produced 5.43 unique solutions out of 6 trials in the low-appeal treatment, compared to 4.61 in the high-appeal treatment. Across all experimental replicates, 77.8% of solutions were unique in the low-appeal treatment, compared to only 52.4% in the high-appeal treatment. Figure 3 shows the cumulative number of unique solutions across trials, illustrating that exploration was broader in the low-appeal condition. Reduced exploration was also apparent in analyses of within-participant solution diversity across generations, measured by the pairwise distance between solutions in six trials. Within-participant diversity tended to decrease across generations in both treatments, but remained overall lower in the high-appeal treatment (Generation: *t*(440.00) = − 4.72, *p* < 0.001, *b* = − 1.59, 95% CI = [−2.26, −0.93]; Low-appeal: *t*(125.97) = 4.13, *p* < 0.001, *b* = 4.40, 95% CI = [2.32, 6.49]; see Supplementary Fig. 4 and Supplementary Table 3 for details). Additionally, relative to the high-appeal treatment, the low-appeal treatment showed greater within-chain solution distance (*t*(136.59) = 3.13, *p* = 0.002, *b* = 4.85, 95% CI = [1.81, 7.88]) and greater between-chain solution distance (*t*(127.50) = 6.31, *p* < 0.001, *b* = 4.72, 95% CI = [3.25, 6.18]; see Supplementary Fig. 5 and Supplementary Table 4 for details).

**Fig. 4:**
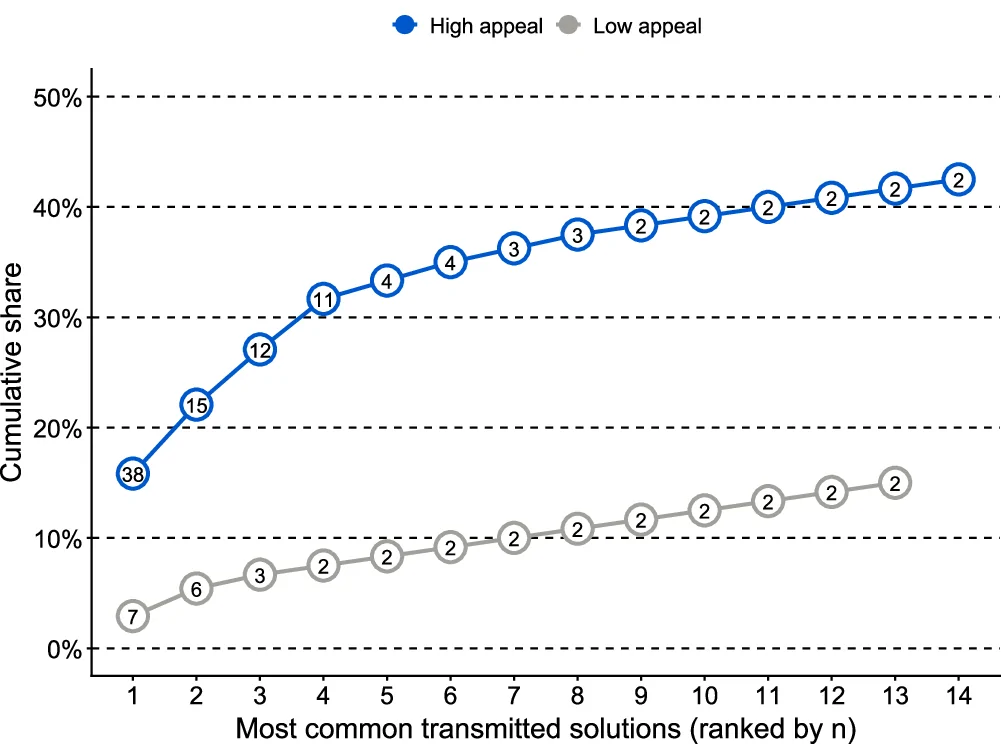
Cumulative share of transmitted solutions. Cumulative proportion of transmitted solutions ranked by frequency in each treatment. In the high-appeal treatment, a relatively small number of solutions account for a substantial share of all transmitted solutions. In the low-appeal condition, transmitted solutions are more evenly distributed across a broader range of configurations, with no single solution dominating to the same extent. Numbers inside the circles indicate how many times each solution was transmitted. Only solutions observed more than once are shown. Grey circles correspond to the low-appeal treatment, and blue circles correspond to the high-appeal treatment.

Analyses of the solutions produced in the sixth trial—that is, the solutions transmitted between generations—reveal important differences between treatments. In the high-appeal treatment, the most common solution accounted for about 16% of all transmitted solutions (38 of 240, Fig. 4), and the two most common solutions together accounted for about 22% of transmitted solutions (53 of 240). In the low-appeal treatment, no single solution was observed more than seven times, and the two most common solutions accounted for about 5% of all transmitted solutions (13 of 240). Overall, solutions observed more than once represented about 42.5% of all transmitted solutions in the high-appeal treatment (102 of 240 solutions across 14 unique solutions), compared with about 15% in the low-appeal treatment (36 of 240 solutions across 13 unique solutions).

**Fig. 5:**
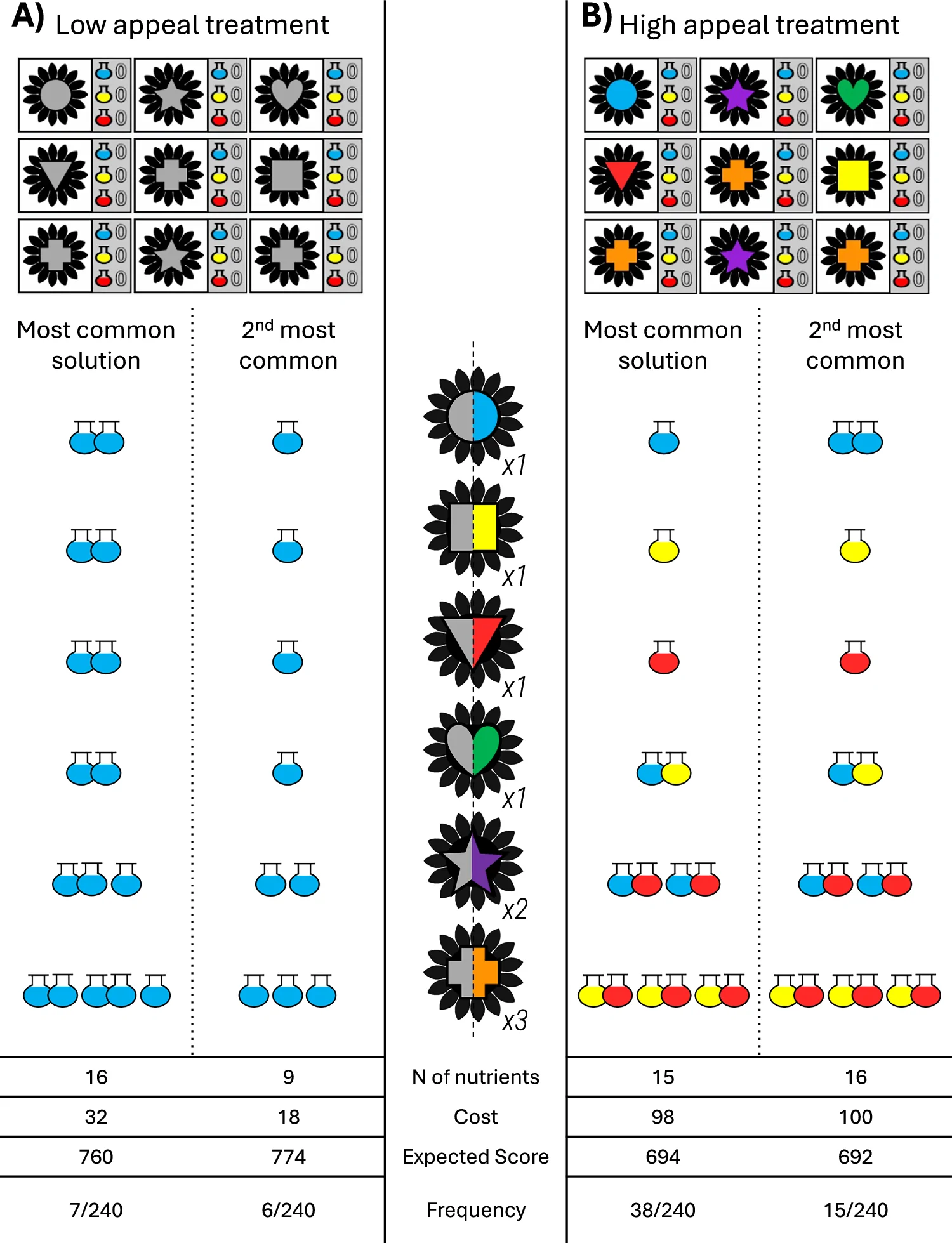
Most common culturally transmitted solutions. The central column shows the composition of the field, alongside the nutrient configurations used in the most common solutions for each treatment. Note that the star-shaped/violet and cross-shaped/orange plants appear twice and three times, respectively, in the field. **A** The two most common culturally transmitted solutions in the low-appeal treatment exclusively involve blue nutrients. The most common solution in this treatment uses 16 blue nutrients, the most efficient solution when all nutrients are used. The second most common solution involves 9 blue nutrients—one for each plant—which is the optimal solution. **B** In the high-appeal treatment, the two most common solutions use a mix-and-match strategy, where primary-colored plants are fed the corresponding colored nutrient, while secondary-colored plants receive a mix of colored nutrients.

Figure 5 illustrates how the most common solutions visually differed between treatments. In the low-appeal treatment, the two most common solutions involved only blue nutrients: the most common used 16 blue nutrients, while the second most common—which was the optimal solution—used nine blue nutrients, one per plant (Fig. 5). In the high-appeal treatment, the most common solutions followed a costly “mix-and-match” strategy: participants matched nutrient colors to flower colors whenever possible, and when exact matches were not possible, they combined nutrient colors based on primary-color mixing (e.g., mixing blue and red nutrients for a purple flower; Fig. 5). These mix-and-match solutions cost approximately five times more than the optimal solution.

Analyses of the written advice show that advice messages in the high-appeal treatment were semantically more similar than those in the low-appeal treatment, despite also being longer (see Supplementary Fig. 7 for details). A classification of advice content indicates that participants in the high-appeal treatment transmitted advice consistent with the transmitted nutrient allocation patterns, most often recommending the “mix-and-match” strategy throughout the experiment. By contrast, advice in the low-appeal treatment increasingly recommended favoring blue nutrients over generations, shifting from relatively uncommon in the first generation to being frequently transmitted by the end of the experiment (see Supplementary Fig. 8).

## Discussion

Our findings demonstrate that changes to the visual appearance of stimuli, by increasing the opportunity for cognitive biases to interfere with expectations of efficacy, can hinder cumulative cultural evolution. In the low-appeal treatment, where all plants were grey, solutions became progressively more efficient across generations. In contrast, in the high-appeal treatment, where plants were colorful, participants produced costly, ineffective solutions and showed no statistically significant improvement across generations.

This failure to shift toward more efficient solutions in the high-appeal treatment appears to be driven by reduced exploration both within and across participants. Our findings suggest that this lower level of exploration reflects the strong appeal of the “mix-and-match” strategy in the high-appeal treatment, wherein participants aligned nutrient colors with flower colors whenever possible or combined nutrient colors based on primary-color mixing. The appeal of this strategy is evident in several ways. First, participants in the high-appeal treatment produced fewer unique solutions, indicating a shift toward greater exploitation in the exploration–exploitation dilemma faced by participants. This pattern is consistent with the idea that cognitive biases shaped participants’ expectations of efficacy in our experiment. Second, when participants did explore, exploration was more local and more convergent in the high-appeal treatment: successive solutions produced by the same participant were more similar (smaller within-individual solution distances across the six trials), and solutions overlapped more across participants (smaller within-chain and between-chain solution distances).

Limited exploration may also have been self-reinforcing through the cultural transmission of advice. Experiments have shown that receiving a theory can channel exploration toward a specific region of the search space^8,26^. In our experiment, the limited variation in the theories transmitted in the high-appeal treatment, which tended to focus on the mix-and-match solution, would therefore be expected to channel exploration toward a similar region across participants, further constraining the total exploration across individuals. Regardless of the precise mechanism, the observed reduction in variation constrains cultural evolvability—the capacity of a cultural system to generate, retain, and build upon culturally transmitted variation—by limiting the raw material on which selection operates^27^.

Importantly, no statistically significant improvement across generations was observed in the high-appeal treatment, despite participants relying on payoff information when selecting a cultural demonstrator and most often choosing the highest-scoring option. This finding is particularly striking because payoff-biased social learning is known to be critical for the emergence of increasingly efficient solutions^6^. Indeed, in a previous version of the same experiment, in which payoff information was unavailable when participants selected a demonstrator, no statistically significant cumulative improvement was observed in either treatment (see Supplementary Fig. 15 and Supplementary Table 7). Disrupting participants’ ability to identify the highest-performing cultural demonstrator would therefore be expected to hinder cumulative cultural evolution even more than what we observe here.

A likely reason that participants continued to rely on payoff information, despite the opportunity to also judge demonstrators by the appeal of their advice, is that payoff comparison was cognitively straightforward (participants compared three numerical scores), whereas comparing advice required reading and integrating several text messages. Outside the lab, payoff information is often noisier, delayed, or difficult to quantify, which could increase reliance on appeal. A second factor is that strong convergence in the high-appeal condition limited variation in the appeal of competing messages. Consistent with this, advice messages in the high-appeal condition were more homogeneous than in the low-appeal condition and thus less diagnostic for differentiating demonstrators, making payoffs a more informative cue.

The core feature of mix-and-match—a perceptual alignment between the intervention and the target object—mirrors patterns found in magic and folk remedies, exemplifying what anthropologists term the “law of similarity.”^21^ Such cultural patterns suggest that humans reliably develop tendencies to infer causal relationships from visual similarity. The reasons for such causal biases remain debated. Proposals include that people erroneously learn causal relationships from environmental regularities^24^ and that the principle of like-affects-like occurs frequently enough in the natural world that it proved a useful scaffold for understanding causality for our ancestors^22^. Whatever their developmental or evolutionary origins, such intuitions can lead individuals to overestimate causal links between features and effects, such as assuming that a plant’s shape is linked to its medicinal properties or, as in our experiment, that nutrients are more effective when their colors match those of the plants to which they are applied. The exposure to cultural products that harness these intuitions, such as video games, may further amplify such causal associations.

Given the apparent functionality of many cultural practices found across much of culture, researchers have often explained those that seem instrumentally ineffective, such as shamanism, magic, and divination, by proposing alternative functional benefits, such as enhanced confidence or social cohesion^28–30^. However, consistent with recent arguments^14,31,32^, our results experimentally illustrate that ineffective cultural practices can be maintained by subjective appeal. Our experiment investigated a single form of appeal—visual similarity—and the resulting cultural product was much simpler than practices like divination or shamanism. We suspect that these more complex practices tap into other dimensions of subjective appeal beyond those investigated here. Nevertheless, our study serves as proof of concept, demonstrating that functional benefits are not required for the emergence and persistence of costly ineffective cultural practices.

Our study has important implications for studying cumulative cultural evolution under controlled conditions. Although researchers have investigated the conditions that constrain cumulative cultural evolution^5,13,33–38^, most experiments are designed in ways that may artificially inflate the potential for gradual cultural improvement. For example, with few exceptions^12^, researchers typically provide participants with immediate and accurate payoffs, despite many interventions in the real-world leading to payoffs that are noisier and time-delayed. More importantly, the solution space in laboratory experiments is often artificially constrained^39,40^, limiting the opportunity for inefficient but cognitively appealing solutions to compete with more efficient ones. These design choices potentially lead to an underappreciation of the inefficiencies of cumulative cultural evolution as manifested both in practices that are cheap and effective yet rare, such as handwashing^41^, and in practices that are common and costly yet futile.

In our experiment, the high-appeal treatment created opportunities for visual similarity, which we expected would bias how participants evaluated potential solutions. By embedding this cognitive bias into the task environment, we created a setting in which visually appealing—but costly and inefficient—solutions could plausibly emerge. This does not mean, however, that the experiment was artificially tailored to produce such outcomes. First, the patterns observed in the low-appeal treatment confirm that the conditions of the experiment—such as task complexity, payoff noise, and number of demonstrators—were conducive to cumulative cultural evolution. Second, even in the high-appeal treatment, the opportunity for cognitively appealing solutions to emerge was limited. In real-world contexts, learners routinely encounter similarity-based cues alongside many other sources of biased inference. Outside the lab, individuals interact with a broader set of variables—such as timing and other contextual factors—that may trigger additional biases not captured in our design^20^. This broader and more complex inferential landscape may further amplify the emergence and persistence of ineffective solutions, underscoring the importance of understanding how subjective appeal shapes the dynamics of cultural evolution across contexts and domains.

## Methods

### Ethics statement

The study was approved by the Toulouse School of Economics/Institute for Advanced Study in Toulouse Review Board for Ethical Standards in Research (2022-11-001). All participants provided informed consent before taking part in the experiment. All individual information has been anonymized.

### Participants

A total of 480 participants were recruited for the study via Prolific. Eligible participants were required to be between 18 and 35 years old, reside in the United Kingdom, the United States, Ireland, Australia, Canada, or New Zealand, have English as their first language, report no color vision deficiencies, and have a Prolific approval rate of 98–100%. Participants received a £1.20 participation fee, with an average bonus of £1.80 based on their performance. Participants reported a mean age of 27.8 in the low-appeal treatment and 28.0 in the high-appeal treatment. The share of female participants was 51.7% in the low-appeal treatment and 47.1% in the high-appeal treatment.

### Experimental task

The experiment relied on a plant-feeding task in which participants were presented with a virtual field containing nine plants (Fig. 1A). Participants aimed to maximize the output of their virtual field across six trials by feeding the plants with three types of nutrients that varied in their cost: blue (2 units), yellow (6 units), and red (10 units). Participants’ decisions in each trial would impact the output of their field and the cost of their solution, which affected their score for that trial. The field’s output was determined by the number of plants that received at least one nutrient and a random component. Additional nutrients on the same plant, the shapes and colors of plants, or the color of the nutrients used had no effect on the output. Specifically, the field’s output was the number of plants fed, multiplied by a uniformly distributed number between 60 and 100. The participant’s score for that trial was then obtained by subtracting the total nutrient cost and adding a fixed score of 72:

$${{\rm{Score}}}=72+{{\rm{Uniform}}}(60,100) \times {{\rm{Number}}}\;{{\rm{of}}}\;{{\rm{plants}}}\;{{\rm{fed}}} - {{\rm{Total}}}\; {{\rm{cost}}}\; {{\rm{of}}}\; {{\rm{nutrients}}}$$

The fixed score of 72 points was added to prevent participants from receiving negative scores in the worst-case scenario.

Participants were limited to using a maximum of 16 nutrients across the entire field, with each plant receiving no more than 9 nutrients of a given color. Under these constraints, trial scores could range from 0 to 954. The optimal strategy (feeding each plant a single blue nutrient) yielded an average score of 774. However, in the best-case scenario, the field could produce an output of 900 (9 plants × 100), at a cost of 18, resulting in a total score of 72 + 900 − 18 = 954. In contrast, the worst strategy (feeding a single plant 9 red and 7 yellow nutrients) yielded an average score of 20. In the worst-case scenario, this approach could produce an output of 60 at a cost of 132, resulting in a total score of 72 + 60 − 132 = 0.

Participants were informed about the random component affecting their score. At the end of each trial, they were shown their score for that trial. However, they were not told the exact function governing the field’s output and the costs of the nutrients.

### Treatments

The study implemented two treatments: low-appeal and high-appeal (see Fig. 1A). A total of 480 participants were equally and randomly assigned to one of the two conditions: high-appeal (*n* = 240) and low-appeal (*n* = 240). Each condition consisted of 20 transmission chains, each comprising four generations of three participants, for a total of 12 participants per chain. In the low-appeal treatment, all plants were grey and exhibited one of six distinct shapes: circle, star, heart, triangle, cross, and square (see Fig. 1A, upper figure). The high-appeal treatment featured the same shapes but in distinct colors: the circle was blue, the star was purple, the heart was green, the triangle was red, the cross was orange, and the square was yellow (see Fig. 1A, lower figure). All participants in each treatment saw the same fixed arrangement of plants. In both treatments, the shapes and colors of the plants were purely aesthetic differences and did not influence the output of the field.

### Cultural transmission

After completing their six trials, participants were required to provide a piece of advice to the next participants in their chain (see Fig. 1B). To ensure comparability across responses, the advice was pre-formatted to begin with the phrase “*The field yields a higher output when…*”.

Except those in the first generation, participants began the experiment by viewing three pieces of advice written by participants in the previous generation, along with the score from their final trial, and selected one participant as their cultural demonstrator. Once a cultural demonstrator had been selected, participants were shown the chosen advice and the corresponding solution (i.e., which nutrients were provided to each plant) for the remainder of the experiment.

### Statistical analysis

#### Score and cost of transmitted solutions

We used the transmitted solutions of each of the 480 participants as the unit of analysis in our models. The response variable was each participant’s expected score—that is, the score excluding the effect of the random component—to reduce the impact of artificial noise on the statistical analyses. To compare how the score changed over the course of the experiment within each treatment, we first fitted two separate models, one for each treatment (*n* = 240 for each, *n*_chain_ = 20). We then fitted a model to the pooled data (high-appeal: *n* = 240, low-appeal: *n* = 240, *n*_chain_ = 40), including generation, treatment, and their interaction as fixed effects to test whether changes across generations differed between treatments. We repeated those three models using participants’ costs as the response variable. In all models, we accounted for chain-specific random effects.

#### Social learning strategies

The response variable was a binary indicator of whether participants selected the highest-scoring demonstrator among the three available cultural models. We used a multilevel model with treatment as a fixed effect and included chain and generation as random effects.

#### Distance measure

For analyses comparing configurations, we used a distance measure based on representing each solution as a vector of nutrient counts, i.e., an ordered sequence of 27 non-negative integers recording how many blue, yellow, and red nutrients each of the nine plants received. This representation is consistent across all solutions, allowing direct numerical comparison. To quantify the difference between solutions, we computed the Manhattan distance between their respective vectors. We computed the mean pairwise distance as the average distance between all possible pairs of solutions in the set. Higher values of mean pairwise distance indicate that solutions in the set are more dispersed and varied, while lower values indicate convergence toward similar solutions. We used this measure to quantify within-individual exploration across trials, as well as convergence of solutions within and between transmission chains^42,43^.

#### Statistical models

All statistical tests reported in the manuscript were evaluated using mixed-effect models with chain-level random intercepts. All fixed-effect tests were two-tailed. We assessed model assumptions, normality of the residuals and homoscedasticity, using standard diagnostics applied to the conditional residuals after accounting for the fitted fixed effects and chain structure. When assumptions were not met, additional robustness checks were run using heteroscedastic linear mixed-effects models. The direction and substantive interpretation of the main treatment-by-generation effects were stable across these checks.

#### Software

The experimental task was implemented using oTree (v5.8.5)^44^. Interactive visual elements were developed using PixiJS (v6.5.8). Data processing and statistical analyses were conducted in R (v4.4.2)^42^ using the tidyverse (v2.0.0)^45^ and lme4 (v1.1-36)^43^ packages, and in Python (v3.9.18) using pandas (v3.0.1) and sentence-transformers (v5.2.3).

#### Pre-registration

The experiment was pre-registered prior to data collection (26 June 2024) on the platform AsPredicted with the number #180,876 (https://aspredicted.org/83mj-fztn.pdf). The study design, sample size, treatment assignment, transmission-chain structure, and primary analyses were preregistered. There were no substantial deviations from the pre-registration.

## Supplementary information

Supplementary Information

Transparent Peer Review File

## Data Availability

The data for this study is publicly available at https://doi.org/10.5281/zenodo.21210938.

## Code availability

The code for the analyses is publicly available at https://doi.org/10.5281/zenodo.21210938.
