# Supplementary material for: Cognitive appeal promotes the persistence of inefficient solutions and hinders cumulative cultural evolution
Source: Nat Commun. 2026 Sep 12;17:10752. doi: 10.1038/s41467-026-77677-9 (PMC13661520; doi:10.1038/s41467-026-77677-9)

Supplementary Information

for

Cognitive appeal promotes the persistence of inefficient solutions and hinders cumulative cultural evolution

Ali Seyhun Saral[1], Manvir Singh[2,*], Antoine Jacquet[3], Haneul Jang[1], Maxime Derex[1,4*]

[1] Institute for Advanced Study in Toulouse; [2] University of California, Davis
[3] Sciences Po, Paris; [4] Toulouse School of Economics, IAST, CNRS, University of Toulouse Capitole.

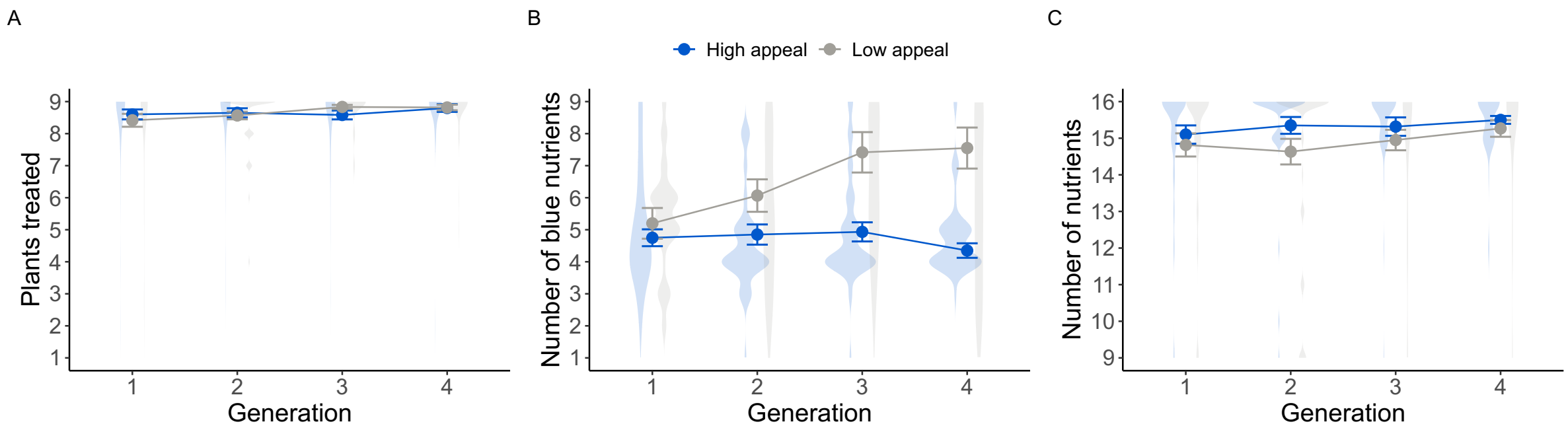

Supplementary Fig.1. Patterns of nutrient use. (A) Mean number of plants treated (i.e., plants receiving at least one nutrient). (B) Mean number of blue nutrients used. (C) Mean total number of nutrients used. Across treatments, participants consistently fed most plants (A) and used most available nutrients (C). However, in the low-appeal condition, the use of blue nutrients—the least costly nutrient—increased across generations. In contrast, blue nutrient use remained stable across generations in the high-appeal condition. Total sample size: N=480, with n=60 observations contributing to each mean and violin plot. Data are presented as mean values and error bars indicate ±1 standard error of the mean (SEM). Blue indicates the high-appeal treatment, and grey indicates the low-appeal treatment.

Term	Estimate (b)	SE	95% CI	df	t	p
**A Plants treated**						
Model 1 (n=480, num. chains = 40)						
(Intercept)	8.51	0.09	[8.33, 8.69]	480.00	92.23	<0.001
Low-Appeal	0.00	0.09	[-0.19, 0.19]	480.00	0.00	1.000
Generation	0.10	0.04	[0.02, 0.18]	480.00	2.36	0.019
Model 2 (n=480, num. chains = 40)						
(Intercept)	8.58	0.11	[8.36, 8.80]	480.00	76.70	<0.001
Low-Appeal	-0.14	0.16	[-0.45, 0.17]	480.00	-0.89	0.377
Generation	0.05	0.06	[-0.06, 0.17]	480.00	0.89	0.373
Low-Appeal:Generation	0.09	0.08	[-0.07, 0.26]	480.00	1.10	0.270
**B Blue nutrients**						
Model 1 (n=480, num. chains = 40)						
(Intercept)	4.17	0.46	[3.27, 5.08]	56.85	9.00	<0.001
Low-Appeal	1.84	0.60	[0.66, 3.01]	40.00	3.06	0.004
Generation	0.36	0.12	[0.12, 0.61]	440.00	2.92	0.004
Model 2 (n=480, num. chains = 40)						
(Intercept)	4.89	0.50	[3.91, 5.86]	74.72	9.82	<0.001
Low-Appeal	0.41	0.70	[-0.97, 1.79]	74.72	0.58	0.562
Generation	-0.11	0.17	[-0.45, 0.23]	440.00	-0.64	0.520
Low-Appeal:Generation	0.95	0.25	[0.47, 1.43]	440.00	3.88	<0.001
**C Total nutrients**						
Model 1 (n=480, num. chains = 40)						
(Intercept)	15.10	0.20	[14.71, 15.50]	94.78	75.58	<0.001
Low-Appeal	-0.40	0.23	[-0.84, 0.04]	40.00	-1.77	0.085
Generation	0.14	0.08	[-0.01, 0.30]	440.00	1.77	0.077
Model 2 (n=480, num. chains = 40)						
(Intercept)	15.14	0.23	[14.68, 15.60]	161.64	64.98	<0.001
Low-Appeal	-0.48	0.33	[-1.12, 0.17]	161.64	-1.44	0.151
Generation	0.12	0.11	[-0.10, 0.34]	440.00	1.03	0.302
Low-Appeal:Generation	0.05	0.16	[-0.26, 0.36]	440.00	0.31	0.754

Supplementary Table 1. Linear mixed-effects model estimates for three dependent variables in transmitted solutions: (A) number of plants treated, (B) number of blue nutrients used, and (C) total number of nutrients used. For each outcome, separate models were fit to pooled data across treatments and generations. To account for the nested data structure, all models included a random intercept for transmission chain. Fixed effects were tested using two-sided Wald t-tests with Satterthwaite-approximated degrees of freedom, and uncertainty is reported as 95% Wald confidence intervals Model baselines: Generation=1, Treatment: High-Appeal

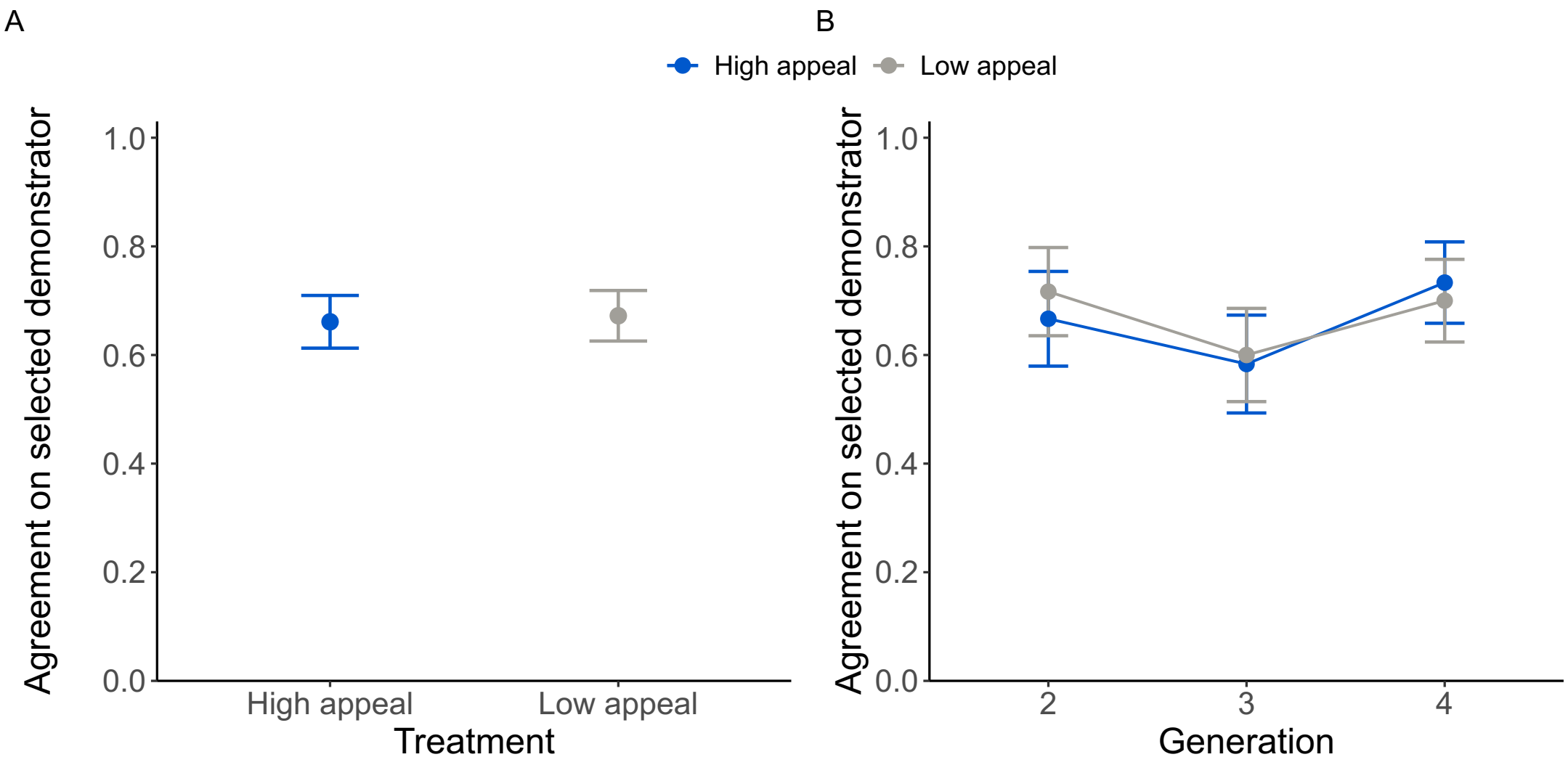

Supplementary Fig. 2. Agreement among learners in demonstrator selection. (A) Mean pairwise agreement within each treatment. (B) Mean pairwise agreement by generation and treatment. Agreement is computed as the proportion of learner pairs within a chain who chose the same demonstrator. It takes three values within a chain/generation consisting of three participants: 1 when all three participants agree (all three pairs), 0.33 when exactly two participants agree (one agreeing pair out of three), and 0 when all three participants chose different demonstrators (no agreeing pairs). Levels of agreement are similar across treatments and remain relatively stable across generations, indicating comparable patterns of demonstrator selection in the high- and low-appeal conditions. Total sample size: N=360 (Panel A. n=180 each mean. Panel B. n=60 observations contributing to each mean). Data are presented as mean values and error bars indicate ±1 standard error of the mean (SEM). Blue indicates the high-appeal treatment condition, whereas grey indicates the low-appeal treatment condition.

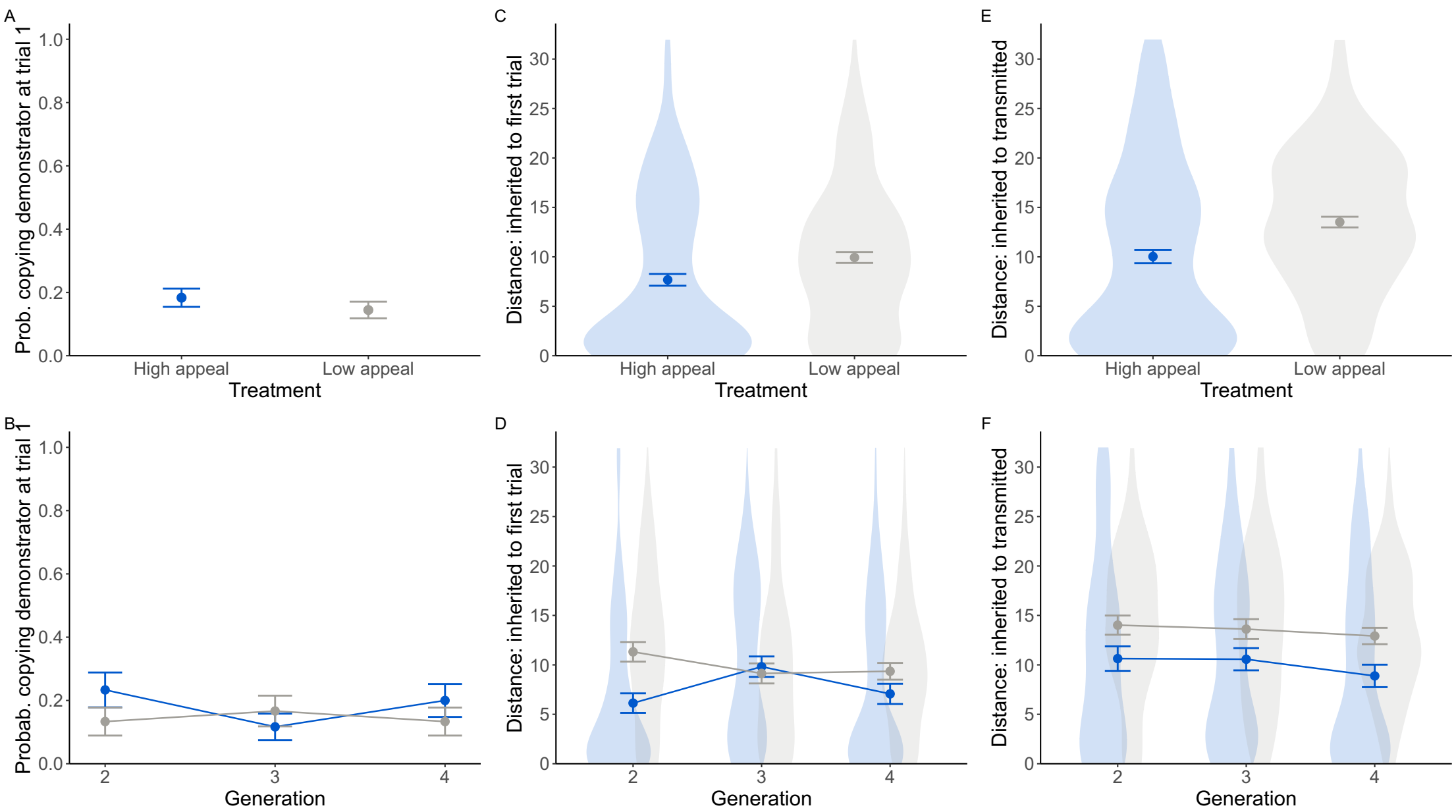

Supplementary Fig. 3. Social influence. (A–B) Probability of copying the inherited solution at Trial 1, defined as the proportion of participants whose first-trial solution exactly matched the inherited solution from their selected demonstrator. (C–D) Mean distance between the inherited solution and the solution used at Trial 1. (E–F) Mean distance between the inherited solution and the transmitted solution. Distance is measured as the sum of absolute differences across all nutrient allocations in the solution vector. Panels A, C, and E show treatment-level means, whereas panels B, D, and F show means by treatment and generation. Total sample size: N=360 (Panels A, C, E: n=180 observations contributing to each boxplot and violin plot; Panels B, D, F: n=60 observations contributing to each boxplot and violin plot). Blue indicates the high-appeal treatment condition, whereas grey indicates the low-appeal treatment condition. Data are presented as mean values and error bars indicate ±1 standard error of the mean (SEM).

Term	Estimate (b)	SE	95% CI	df	t	p
**A Copy probability**						
Model 1 (n=360, num. chains = 40)						
(Intercept)	0.19	0.04	[0.11, 0.27]	78.93	4.56	<0.001
Low-Appeal	-0.04	0.05	[-0.14, 0.06]	40.00	-0.78	0.441
Generation	-0.01	0.02	[-0.05, 0.04]	320.00	-0.36	0.716
Model 2 (n=360, num. chains = 40)						
(Intercept)	0.20	0.05	[0.11, 0.29]	124.43	4.18	<0.001
Low-Appeal	-0.06	0.07	[-0.19, 0.08]	124.43	-0.82	0.414
Generation	-0.02	0.03	[-0.08, 0.05]	320.00	-0.52	0.607
Low-Appeal:Generation	0.02	0.05	[-0.07, 0.11]	320.00	0.36	0.716
**B Distance between inherited solution and the first trial**						
Model 1 (n=360, num. chains = 40)						
(Intercept)	7.93	0.93	[6.12, 9.74]	70.61	8.57	<0.001
Low-Appeal	2.26	1.13	[0.04, 4.48]	40.00	2.00	0.052
Generation	-0.26	0.47	[-1.17, 0.65]	320.00	-0.56	0.579
Model 2 (n=360, num. chains = 40)						
(Intercept)	7.21	1.03	[5.18, 9.23]	105.75	6.97	<0.001
Low-Appeal	3.71	1.46	[0.85, 6.58]	105.75	2.54	0.013
Generation	0.47	0.66	[-0.82, 1.75]	320.00	0.71	0.477
Low-Appeal:Generation	-1.45	0.93	[-3.27, 0.37]	320.00	-1.57	0.119
**C Distance between the inherited solution and transmitted solution**						
Model 1 (n=360, num. chains = 40)						
(Intercept)	10.74	1.04	[8.71, 12.77]	64.50	10.38	<0.001
Low-Appeal	3.49	1.30	[0.95, 6.03]	40.00	2.69	0.010
Generation	-0.71	0.48	[-1.66, 0.23]	320.00	-1.48	0.140
Model 2 (n=360, num. chains = 40)						
(Intercept)	10.90	1.14	[8.67, 13.14]	92.76	9.55	<0.001
Low-Appeal	3.16	1.61	[-0.00, 6.33]	92.76	1.96	0.053
Generation	-0.87	0.68	[-2.21, 0.46]	320.00	-1.29	0.199
Low-Appeal:Generation	0.32	0.96	[-1.56, 2.21]	320.00	0.34	0.736

Supplementary Table 2. Linear mixed-effects model estimates for three social-influence outcomes: (i) probability of copying the inherited solution at Trial 1, (ii) distance between the inherited solution and the solution used at Trial 1, and (iii) distance between the inherited and transmitted solutions. For each outcome, separate models were fit to pooled data. To account for the nested data structure, all models included a random intercept for transmission chain. Fixed effects were tested using two-sided Wald t-tests with Satterthwaite-approximated degrees of freedom, and uncertainty is reported as 95% Wald confidence intervals. Model baselines: Generation=2, Treatment: High-Appeal (pooled-model)

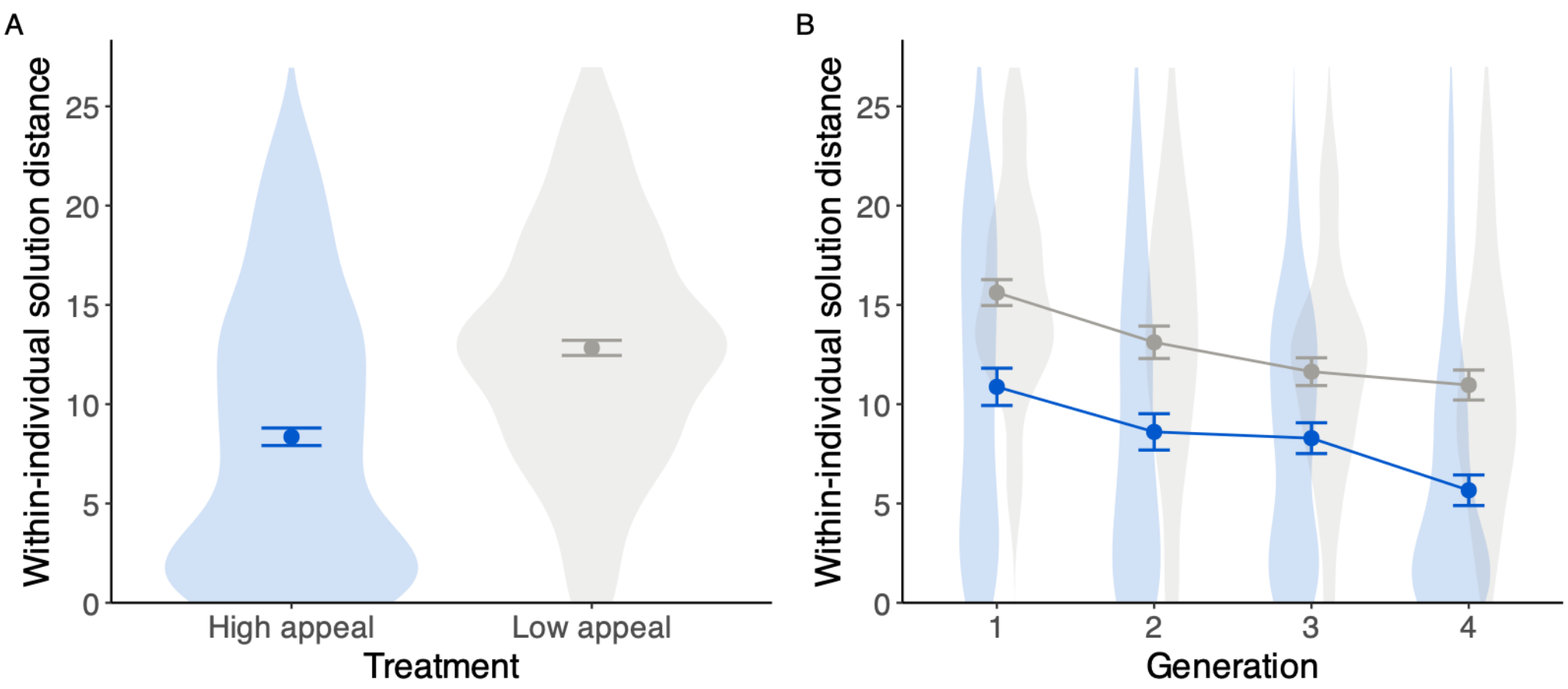

Supplementary Fig. 4. Individual exploration. (A) Mean within-individual solution distance by treatment. (B) Mean within-individual solution distance by treatment and generation. Distance is measured as the sum of absolute differences across all nutrient allocations in the solution vector averaged over trial pairs. Larger distances indicate greater within-individual exploration across trials. Total sample size: N=480 (Panel A. n=240 observations contributing to boxplot. Panel B. n=60 observations contributing to each mean and violin plot) Blue indicates the high-appeal treatment condition, whereas grey indicates the low-appeal treatment condition. Data are presented as mean values and error bars indicate ±1 standard error of the mean (SEM).

Term	Estimate (b)	SE	95% CI	df	t	p
**Within-Individual Distance (n=480, num. chains=40)**						
Model 1						
(Intercept)	10.71	0.66	[9.42, 12.01]	78.82	16.18	<0.001
Low-Appeal	4.48	0.79	[2.93, 6.02]	40.00	5.69	<0.001
Generation	-1.57	0.24	[-2.04, -1.10]	440.00	-6.57	<0.001
Model 2						
(Intercept)	10.75	0.75	[9.27, 12.23]	125.97	14.28	<0.001
Low-Appeal	4.40	1.06	[2.32, 6.49]	125.97	4.13	<0.001
Generation	-1.59	0.34	[-2.26, -0.93]	440.00	-4.72	<0.001
Low-Appeal:Generation	0.05	0.48	[-0.89, 0.99]	440.00	0.10	0.917

Supplementary Table 3. Linear mixed-effects model estimates for individual exploration, measured as mean within-individual solution distance. Separate models were fit to pooled data. To account for the nested data structure, all models included random intercepts for transmission chain. Fixed effects were tested using two-sided Wald t-tests with Satterthwaite-approximated degrees of freedom, and uncertainty is reported as 95% Wald confidence intervals. Model baselines: Generation=1, Treatment: High-Appeal (pooled-model)

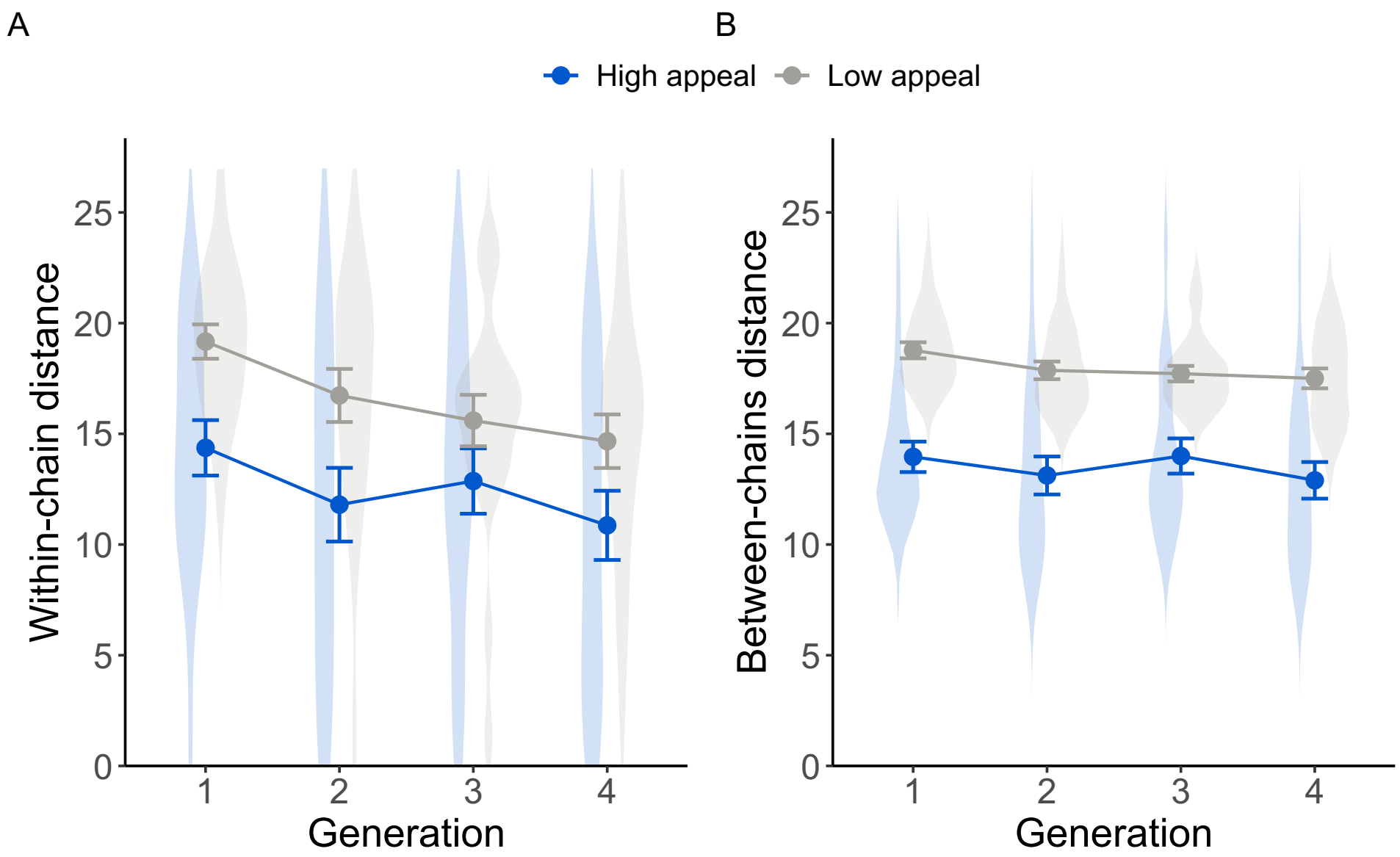

Supplementary Fig. 5. Cultural convergence of transmitted solutions. (A) Within-chain mean pairwise distance among the three transmitted solutions in each chain. (B) Between-chains mean pairwise distance, computed for each chain as the average distance between its three transmitted solutions and all transmitted solutions from other chains in the same treatment-generation group. Total sample size: N=480 (n=60 observations contributing to each mean and violin plot) Data are presented as mean values and error bars indicate ±1 standard error of the mean (SEM). Blue indicates the high-appeal treatment condition, whereas grey indicates the low-appeal treatment condition.

Term	Estimate (b)	SE	95% CI	df	t	p
**Within-chain solution distance**						
Model 1 (n=160, num. chains = 40)						
(Intercept)	14.28	0.93	[12.46, 16.10]	96.82	15.40	<0.001
Low-Appeal	4.07	1.02	[2.08, 6.06]	40.00	4.01	<0.001
Generation	-1.20	0.39	[-1.97, -0.44]	120.00	-3.08	0.003
Model 2 (n=160, num. chains = 40)						
(Intercept)	13.89	1.10	[11.74, 16.04]	136.59	12.68	<0.001
Low-Appeal	4.85	1.55	[1.81, 7.88]	136.59	3.13	0.002
Generation	-0.94	0.55	[-2.03, 0.14]	120.00	-1.71	0.090
Low-Appeal:Generation	-0.52	0.78	[-2.05, 1.01]	120.00	-0.67	0.507
**Between-chains solution distance**						
Model 1 (n=160, num. chains = 40)						
(Intercept)	13.96	0.45	[13.08, 14.85]	88.79	30.82	<0.001
Low-Appeal	4.47	0.51	[3.47, 5.47]	40.00	8.74	<0.001
Generation	-0.31	0.18	[-0.67, 0.04]	120.00	-1.72	0.088
Model 2 (n=160, num. chains = 40)						
(Intercept)	13.84	0.53	[12.80, 14.88]	127.50	26.18	<0.001
Low-Appeal	4.72	0.75	[3.25, 6.18]	127.50	6.31	<0.001
Generation	-0.23	0.26	[-0.73, 0.27]	120.00	-0.90	0.372
Low-Appeal:Generation	-0.16	0.36	[-0.88, 0.55]	120.00	-0.45	0.651

Supplementary Table 4. Linear mixed-effects model estimates for cultural convergence measures: within-chain solution distance and between-chain solution distance. Separate models were fit for each dependent variable using pooled data. To account for the nested data structure, all models included random intercepts for transmission chain. Fixed effects were tested using two-sided Wald t-tests with Satterthwaite-approximated degrees of freedom, and uncertainty is reported as 95% Wald confidence intervals. Model baselines: Generation=1, Treatment: High-Appeal (pooled-model)

Analyses of Written Advice

Quantitative Textual Analysis

To complement the manual coding of advice content, we conducted quantitative analyses of the linguistic properties of participants' written advice (n = 480; 240 per treatment). Before analysis, we removed the fixed prompt prefix ("The field yields a higher output when…") in order to reflect participants' free-response content only. We compared two text measures across treatments and generations: message length, and semantic similarity.

Message Length

Message length was defined as the number of characters in each cleaned advice message. This measure captures the overall verbosity and degree of elaboration in communicated strategies (Supplementary Fig. 6).

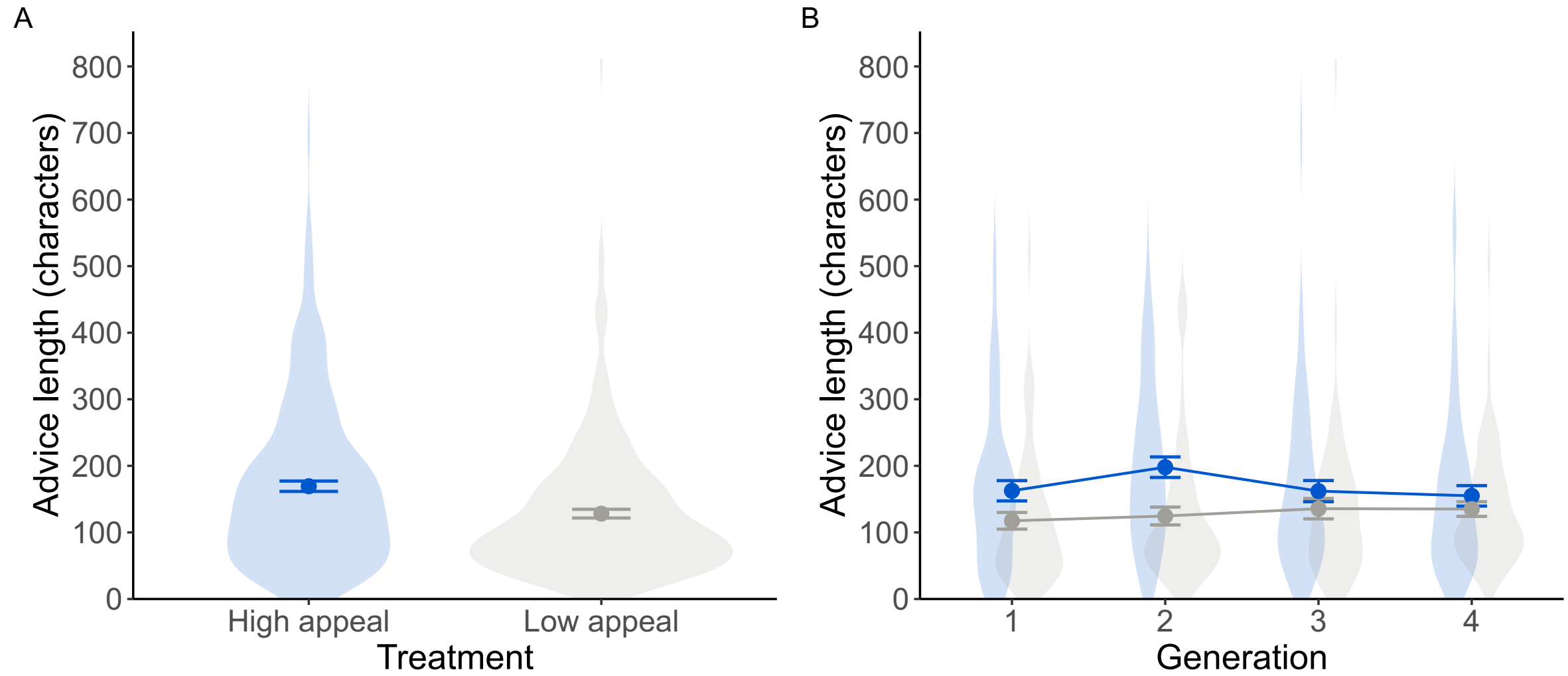

Supplementary Fig. 6. Advice length in characters by generation and treatment. (A) Mean advice length by treatment. (B) Mean advice length by treatment and generation. (Panel A. n=240 observations contributing to each mean and violin plot. Panel B. n=60 observations contributing to each mean and violin plot) Blue indicates the high-appeal treatment condition, whereas grey indicates the low-appeal treatment condition. Data are presented as mean values and error bars indicate ±1 standard error of the mean (SEM).

Semantic Similarity

To quantify convergence in the semantic content of advice messages, we used a sentence-embedding approach. Each advice message was transformed into a fixed-length numerical vector using a pre-trained Sentence-BERT (SBERT) model (all-MiniLM-L6-v2). We then computed pairwise cosine similarity between message vectors. Mean cosine similarity was calculated (i) within each treatment overall and (ii) within each treatment × generation cell by averaging across all pairwise comparisons among messages. This procedure allowed us to examine whether advice became more semantically aligned over generations and whether semantic convergence differed between treatments (Supplementary Fig. 7).

Semantic similarity is initially lower in the low-appeal treatment than in the high-appeal treatment, indicating greater diversity in advice content in early generations. Across generations, similarity increases in the low-appeal condition, whereas changes in the high-appeal condition are comparatively modest. By the fourth generation, both treatments reach a comparable level of within-group semantic similarity.

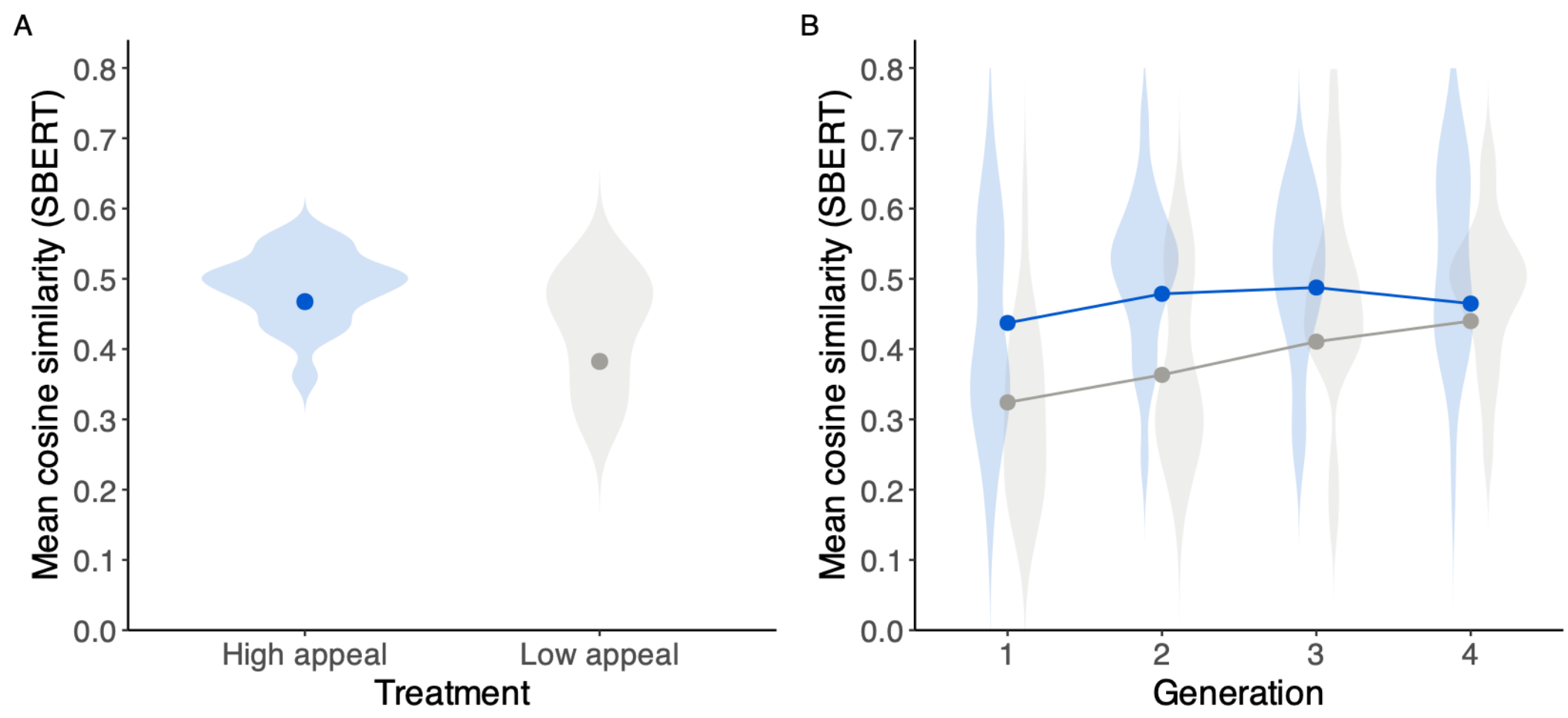

Supplementary Fig. 7. Semantic similarity by treatment and generation. Semantic similarity of transmitted advice messages was measured using SBERT embeddings (all-MiniLM-L6-v2) and cosine similarity. Violin plots show distributions of chain-level mean pairwise similarities (Panel A) and chain-generation-level mean pairwise similarities (Panel B). Overlaid points indicate aggregated means: (A) treatment-level means and (B) treatment x generation means. Higher cosine similarity values indicate greater semantic overlap (values near 1 indicate more similar content; values near 0 indicate weaker overlap). Panel A: n = 240 observations contributed to each treatment-level mean, with 20 chain-level observations per treatment in the violin overlay; this corresponds to 28,680 pairwise comparisons per treatment. Panel B: n = 60 observations contributed to each treatment-by-generation mean, with 20 chain-generation-level observations per treatment-by-generation cell in the violin overlay; this corresponds to 1,770 pairwise comparisons per cell. Blue indicates the high-appeal treatment condition, whereas grey indicates the low-appeal treatment condition. Data are presented as mean value. Standard errors of the mean are omitted as they are too small to plot and uninformative.

Classification of the written advice

In addition to quantitative outcomes, we analyzed the content of participants' written advice to classify how strategies were represented and transmitted across generations. Written advice text pieces were coded into a set of pre-determined strategy categories. Categories that could conceptually be advised together were treated as non-mutually exclusive, allowing multiple strategy elements to be coded within the same message when applicable. We show category frequencies by generation and treatment condition to assess how transmitted messages evolved over time in Supplementary Fig 8. We additionally compared the manual classification to an automated LLM-based classification using GPT-5.1. The two approaches agree with 93% accuracy, and all results and figures are robust to using the LLM-based classification (with an exception due to GPT overcategorizing mix nutrients, resulting in 65% accuracy in that category). The classifications, along with the prompt and script used to generate the GPT-5.1 labels, are provided in the supplementary repository.

**Category**	**Description**	**Example Advice (The field yields a higher output when...)**
Mix & Match	Recommends matching nutrient colors to primary plant colors (blue, yellow, red) when possible and combining nutrients to recreate remaining secondary colors (orange, purple, green) based on color theory.	*...you match the colours according to colour theory. For example, for green, add one blue and one yellow. For purple, add one red and one blue. and so forth. For the already existing primary colours e.g. red, blue or yellow, just add one of the same colour*
Mismatch	Recommends avoiding matching nutrient colors to plant colors (blue, yellow, red), including both simple strategies (using non-corresponding colors) and more complex strategies, such as the opposite of *Mix & Match*, that allocate remaining	*... you use the colours that do not make up the plants colour. If the plant is blue select one each for yellow and red. If the plant is orange, select one for blue as yellow + red = orange so should not be selected.*

	primary colors using color theory to contrast the plant's existing color.	
(Other) Plant Color Based matching	Recommends a strategy in which plant color is the primary cue for nutrient allocation (without explicit *Mix & Match* or *Mismatch* strategies). Includes simple color matching for primary colors only.	*...you focus on matching the colours, I found you got a higher score when prioritising the colours that do not require mixing to match (e.g. giving a red plant two drops of red instead of focusing on giving orange both red and yellow)*
Plant Shape Based Matching	Recommends a strategy in which plant shape is the primary cue for nutrient allocation, including feeding plants with the same shape in the same way.	*...Crosses are Red, Stars are Yellow I can provide that lower output will be achieved when circles are blue and have tested traingles are blue to some succes, Hearts are red .*
Even Out/Spread nutrients	Recommends distributing nutrients broadly or evenly across plants or feeding each plant.	*...the nutrients are spread out.*
Less Nutrients per Plant	Recommends allocating fewer nutrients to each plant without explicitly suggesting even distribution.	*...you use fewer bottles of the solution.*
More Nutrients per Plant	Recommends allocating more nutrients to each plant.	*...you give more than one of the same nutrient per plant.*
Mix Nutrient Colors	Recommends combining multiple nutrient colors within a single plant.	*...you combine two different solutions on the same field*
Pure Nutrients	Recommends avoiding mixing nutrient colors within a plant.	*...I used individual colours by themselves rather than combining other colours together*
Spatial Strategy	Recommends space-based strategies related to the field (e.g., feeding specific rows, columns, corners, or the center in specific ways).	*...blue and yellow is used on the top row. Red and yellow on the middle row.*
Use All/Most Nutrients	Recommends using all or nearly all 16 available nutrients per round.	*...I used all the nutrients i got a higher score*
Time-Based	Recommends strategies based on speed, timing, or temporal pacing.	*...you move fast and submit faster*
Favor Blue Nutrient	Recommends explicitly prioritizing blue nutrients over other colors (or prioritizing blue equally with another nutrient color).	*...you use blue potions*
Favor Yellow Nutrient	Recommends explicitly prioritizing yellow nutrients over other colors (or prioritizing yellow equally with another nutrient color).	*...when I only use the yellow solution instead of only the blue or red*
Favor Red Nutrient	Recommends explicitly prioritizing red nutrients over other colors (or prioritizing red equally with another nutrient color).	*...red nutrients are used compared to yellow and blue nutrients*
Variation	Recommends diversifying allocations, randomizing, or changing strategies across trials.	*...you try to input the colors of the nuitrients randomly on different shapes*
No Information / No Advice	No Information / No Advice<br>Contains no actionable strategic information or refers only to the latest solution.	*...sorry, I really couldn't figure out the pattern for success*
Other	Contains advice that does not fit into the predefined categories.	*...you trial different combinations, find one that generally scores highly, then repeat that.*

Supplementary Table 5. Coding scheme and illustrative examples of written advice. Advice messages were categorized into strategy types based on their substantive content. Categories are treated as not mutually exclusive; a single message could be assigned to multiple categories where applicable. Descriptions summarize the defining features of each category, and example excerpts illustrate typical coded responses. Examples are extracted from actual participant advice keeping the original spelling and grammatical errors.

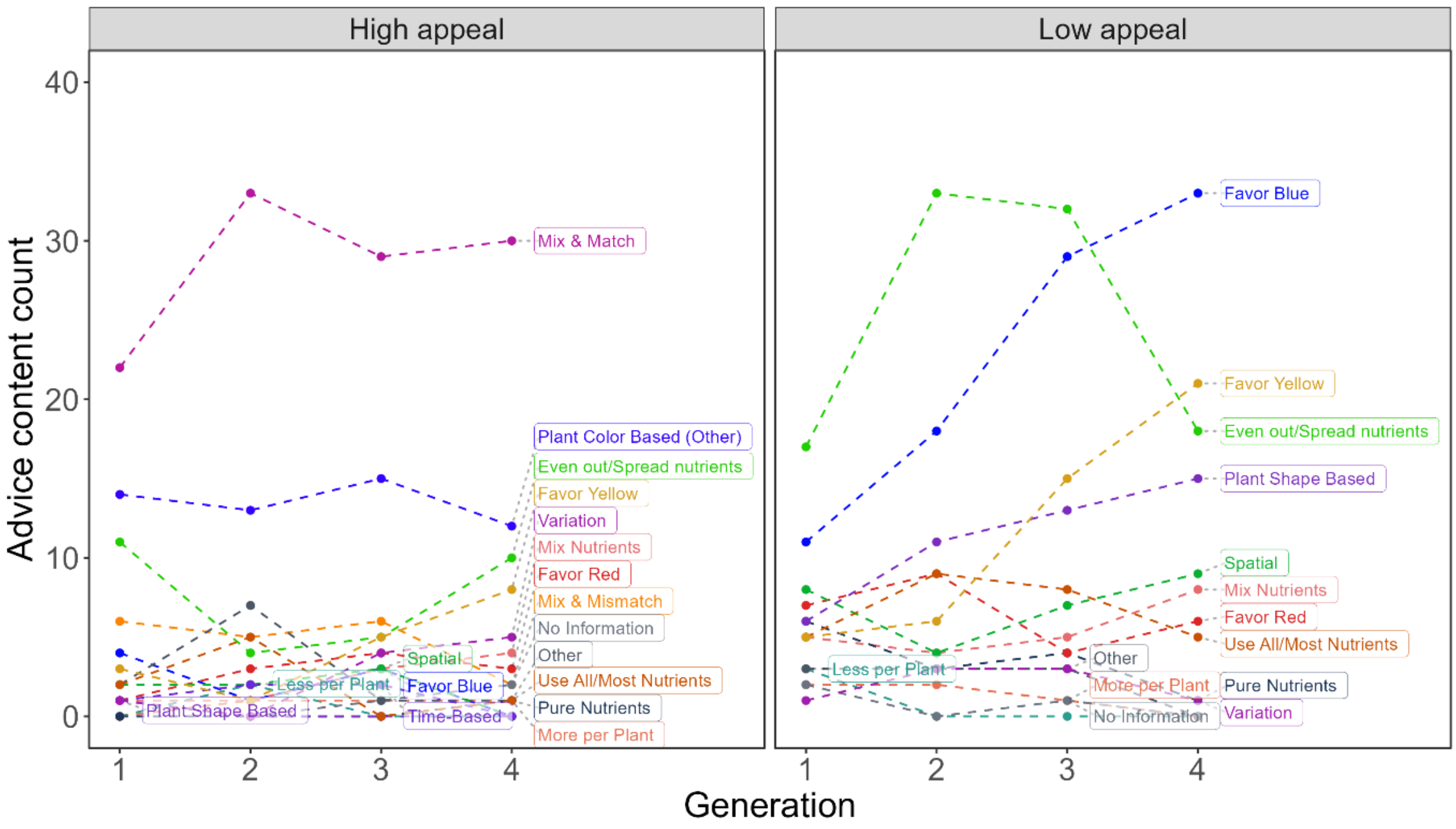

Supplementary Fig. 8. Advice content across generations by treatment conditions. Counts of advice messages coded into a predefined set of strategy categories. In the high-appeal treatment, advice promoting the costly "mix-and-match" strategy increased rapidly in the first two generations and remained relatively stable with a mild decrease. In contrast, in the low-appeal treatment, advice favoring the least costly blue nutrient increased gradually across generations. Advice recommending an even distribution of nutrients—an effective strategy in this task—was more prevalent in the low-appeal treatment. For visual convenience, different colors used for each category.

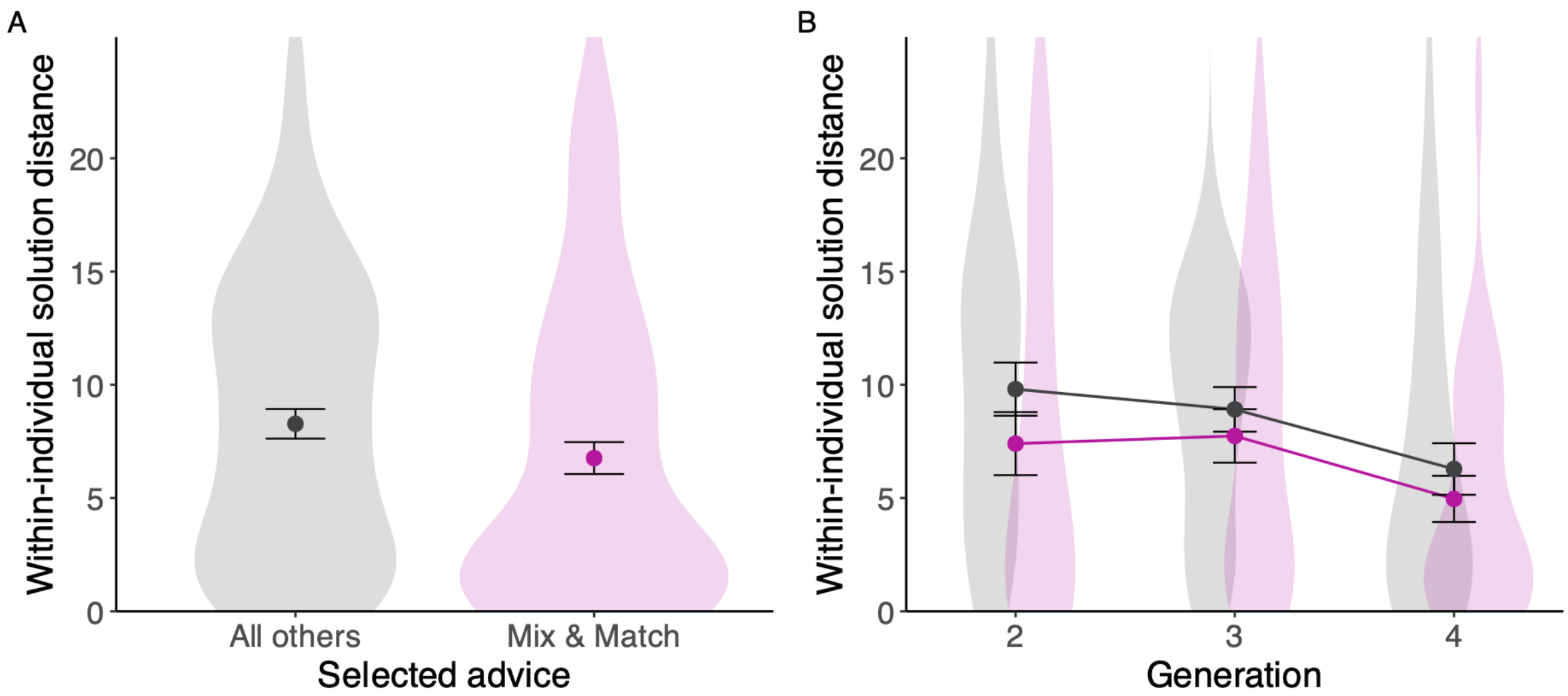

Supplementary Fig. 9. Individual exploration among participants who inherited a mix-and-match solution. (A) Mean within-individual solution distance by treatment. (B) Mean within-individual solution distance by treatment and generation. Distance is measured as the sum of absolute differences across all nutrient allocations in the solution vector, averaged over trial pairs. Error bars represent ±1 SE. Larger distances indicate greater within-individual exploration across trials. Total sample size: N = 180 (high-appeal condition, generations 2-4). Panel A: n = 90 per group (All others, Mix & Match). Panel B: Generation 2 n = 30/30, Generation 3 n = 28/32, Generation 4 n = 32/28 (All others/Mix & Match), with n = 60 per generation. Error bars indicate ±1 standard error of the mean (SEM). Purple indicates participants who inherited a mix-and-match solution whereas grey indicates other participants.

Term	Estimate (b)	SE	95% CI	df	t	p
**A Within-individual solution distance**						
Model 1 (n=180, num. chains = 20)						
(Intercept)	9.78	0.90	[8.02, 11.55]	92.40	10.86	<0.001
Selected Mix & Match	-1.54	0.95	[-3.41, 0.33]	100.48	-1.61	0.110
Generation	-1.50	0.57	[-2.62, -0.37]	159.72	-2.62	0.010
(Intercept)	10.10	1.07	[8.01, 12.20]	117.60	9.45	<0.001
Selected Mix & Match	-2.18	1.50	[-5.11, 0.76]	156.60	-1.46	0.148
Generation	-1.81	0.80	[-3.38, -0.25]	178.88	-2.27	0.024
Selected Mix & Match:Generation	0.66	1.16	[-1.61, 2.92]	171.56	0.57	0.570

Supplementary Table 6. Linear mixed-effects model estimates for individual exploration among participants who chose to inherit the Mix & Match solution. To account for the nested data structure, all models included random intercepts for transmission chain. Fixed effects were tested using two-sided Wald t-tests with Satterthwaite-approximated degrees of freedom, and uncertainty is reported as 95% Wald confidence intervals. Model baselines: Generation=2

Chain Level Dynamics

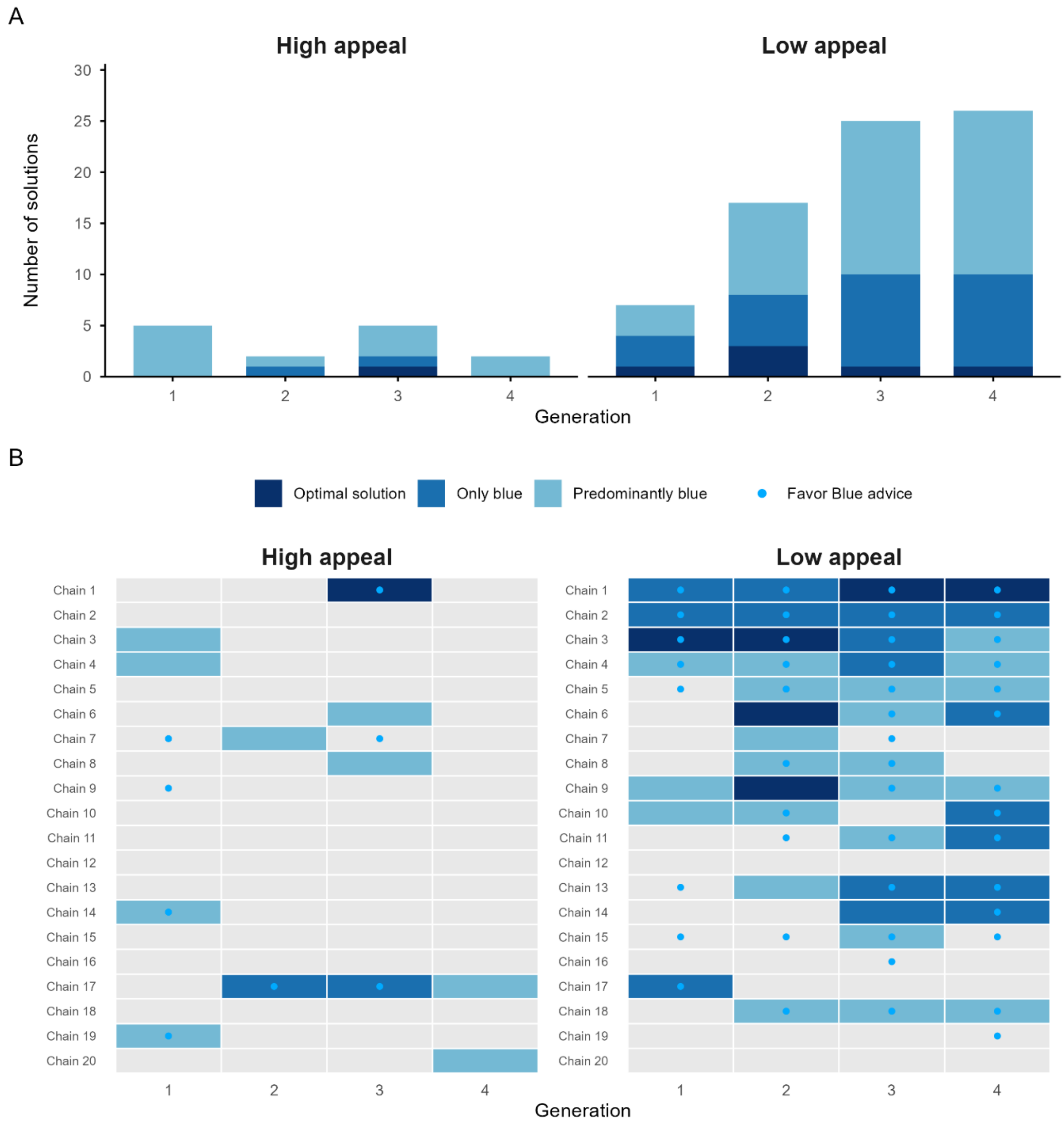

Supplementary Fig. 10. Prevalence of blue-nutrient solutions and written advice favoring blue nutrients. (A) Number of transmitted blue-nutrient heavy solutions per generation, broken down by category: *Optimal solution (only* one blue nutrient for each plant), *Only Blue,* sub-optimal solutions with blue nutrients only (using more than one nutrient each plant or not treating all plants), and solutions that are *blue predominant* (number of blue nutrients is higher than the sum of red and yellow nutrients), for high- and low-appeal conditions. (B) Blue-nutrient status of each chain's transmitted solution at each generation. Each tile reflects the best blue-nutrient strategy solution (Optimal solution > Only blue > Predominantly blue) achieved by any of the three participants within that chain–generation cell. Dots indicate generations in which at least one participant in the cell gave textual advice favoring blue nutrients based on manual classification. Chains are ordered by average expected score of the transmitted solution in the last generation within the chain.

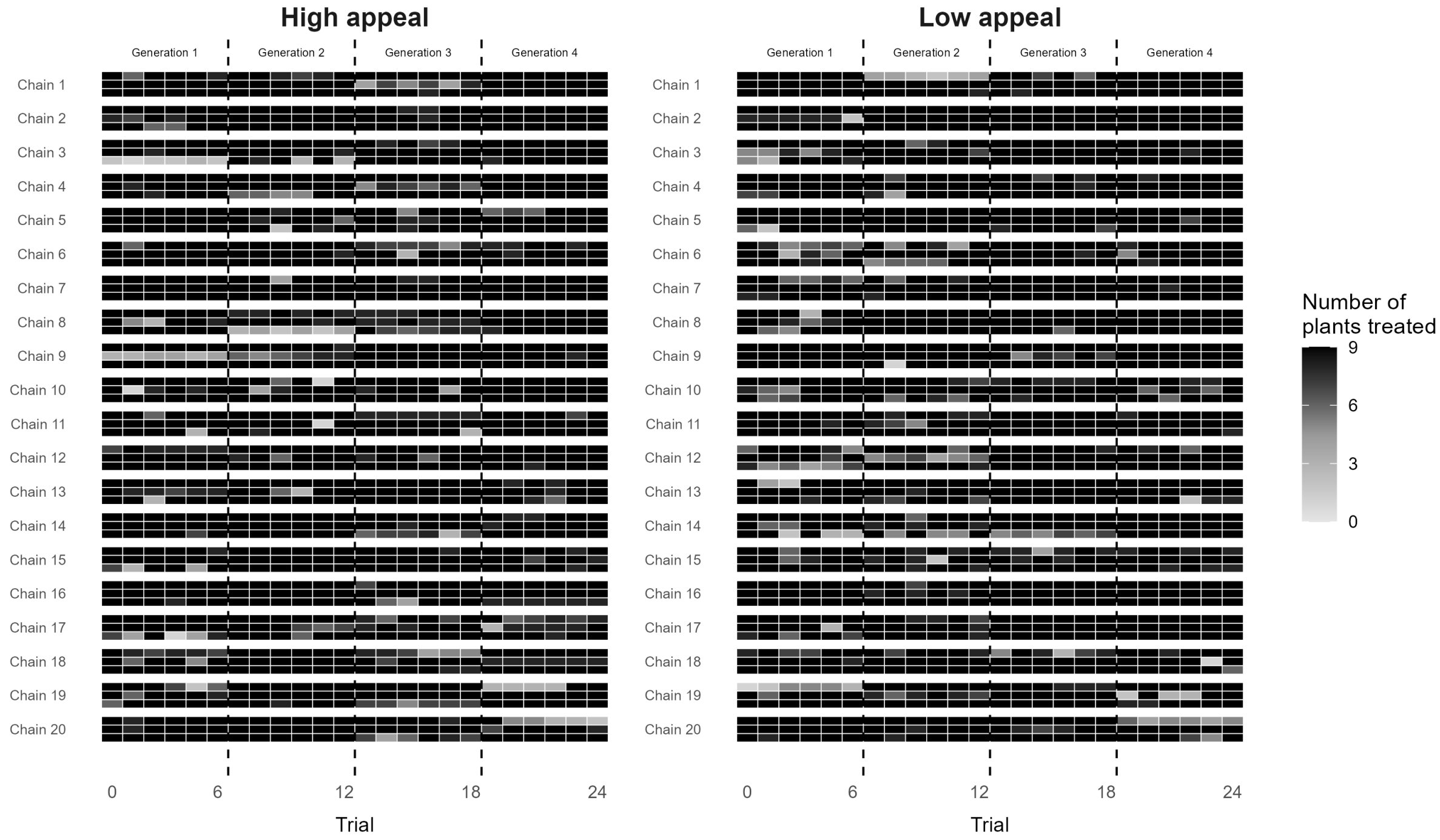

Supplementary Fig 11. Number of plants treated per trial. Each row represents 4 different generations of participants, and a tile represents a trial by a single participant. Rows are grouped by transmission chains (3 adjacent rows). Color indicates the number of plants treated.

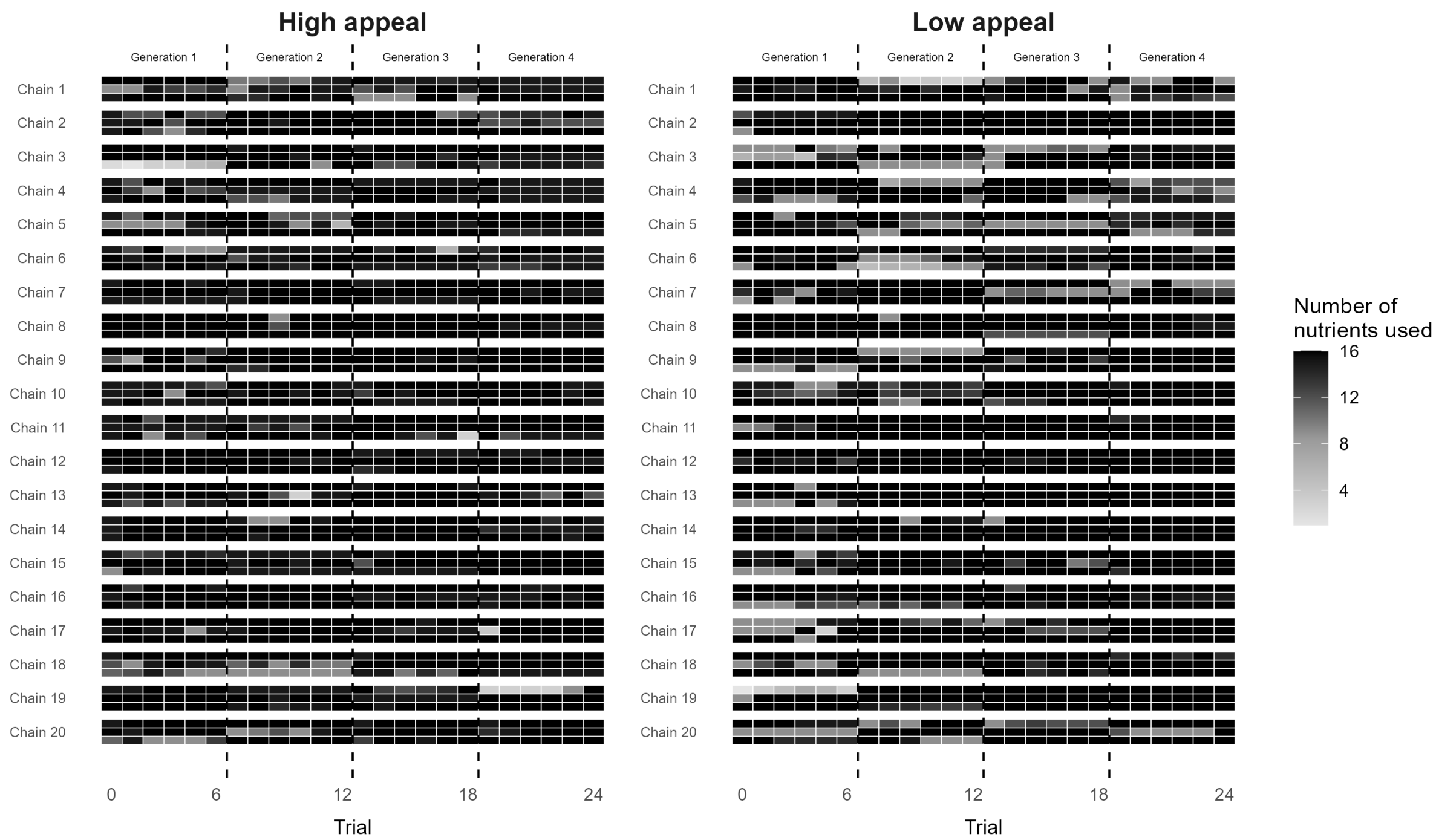

Supplementary Fig. 12. Number of nutrients used per trial. Each row represents 4 different generations of participants, and a tile represents a trial by a single participant. Rows are grouped by transmission chains (3 adjacent rows). Color indicates the number of nutrients used.

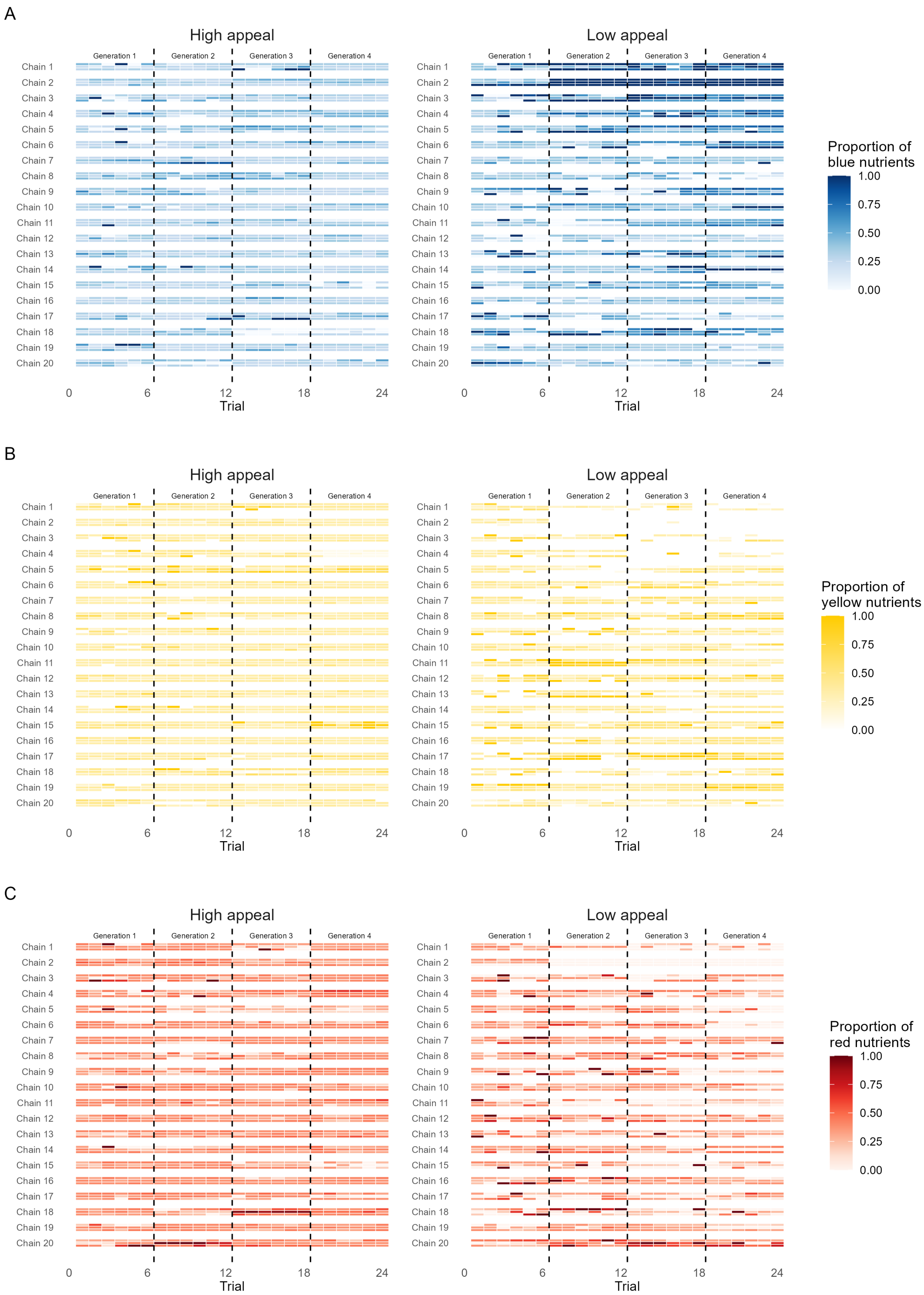

Supplementary Fig. 13. Proportion of each nutrient type per trial. Each row represents 4 different generations of participants, and a tile represents a trial by a single participant. Rows are grouped by transmission chains (3 adjacent rows). (A) Proportion of blue nutrients. (B) Proportion of yellow nutrients. (C) Proportion of red nutrients.

Scores and Costs within Transmission Chains

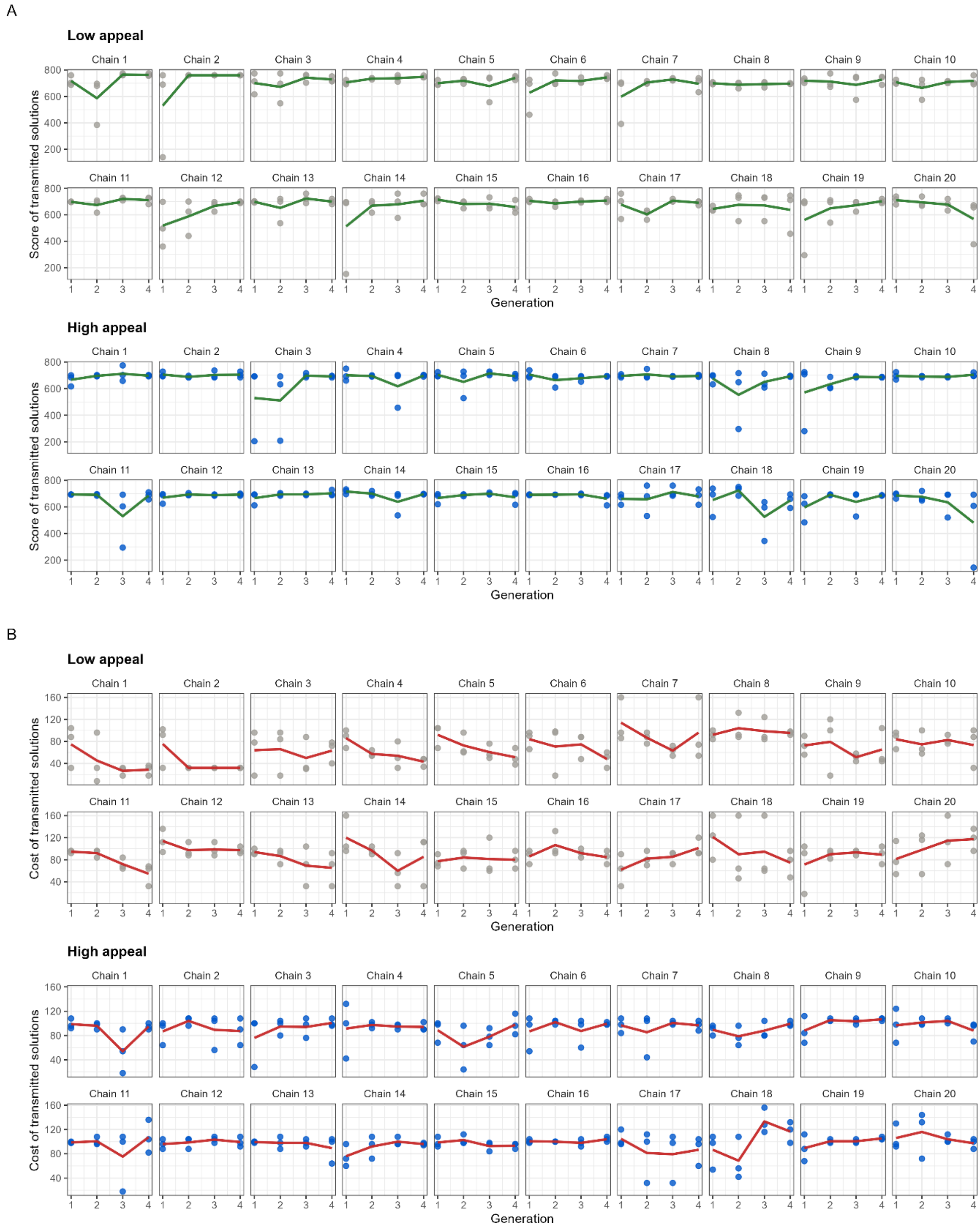

Supplementary Fig. 14. Scores and cost trajectories across chains. (A) Scores (expected) of transmitted solutions within a chain. (B) Costs of transmitted solutions within a chain. Each dot represents a participant's score (or cost) of the transmitted solution. Green lines indicate the average score, and red lines indicate the average cost within the chain. Blue indicates the high-appeal treatment and grey indicates the low-appeal treatment.

Cumulative Cultural Evolution without Payoff-Biased Social Learning

In a previous study, we investigated a variant of this experiment that was identical to the present design except that participants were not provided with information about demonstrators' scores. Participants instead relied exclusively on written advice transmitted by earlier participants prior to selecting a solution. This design allowed for social learning via instruction transmission, while eliminating the possibility of payoff-biased social learning.

Results show no statistically significant evidence of cumulative improvement in scores across generations in either treatment. Although the figures suggest a gradual divergence in solution costs between treatments (Supplementary Fig. 15), this difference is not statistically supported by the regression analyses (Supplementary Table 7). We see a similar pattern of exploration in this variant of the game: In Low-Appeal treatment, participants create a higher number of unique solutions (Supplementary Fig. 16).

Together, these findings suggest that in this task, payoff-biased social learning is necessary but not sufficient for cumulative cultural evolution. While payoff information can overcome payoff noise and enable adaptive refinement in environments with low cognitive appeal, strong biases in expectations of efficacy can erode its benefits, limit the exploration and allow inefficient solutions to persist even under conditions typically favorable to cumulative improvement.

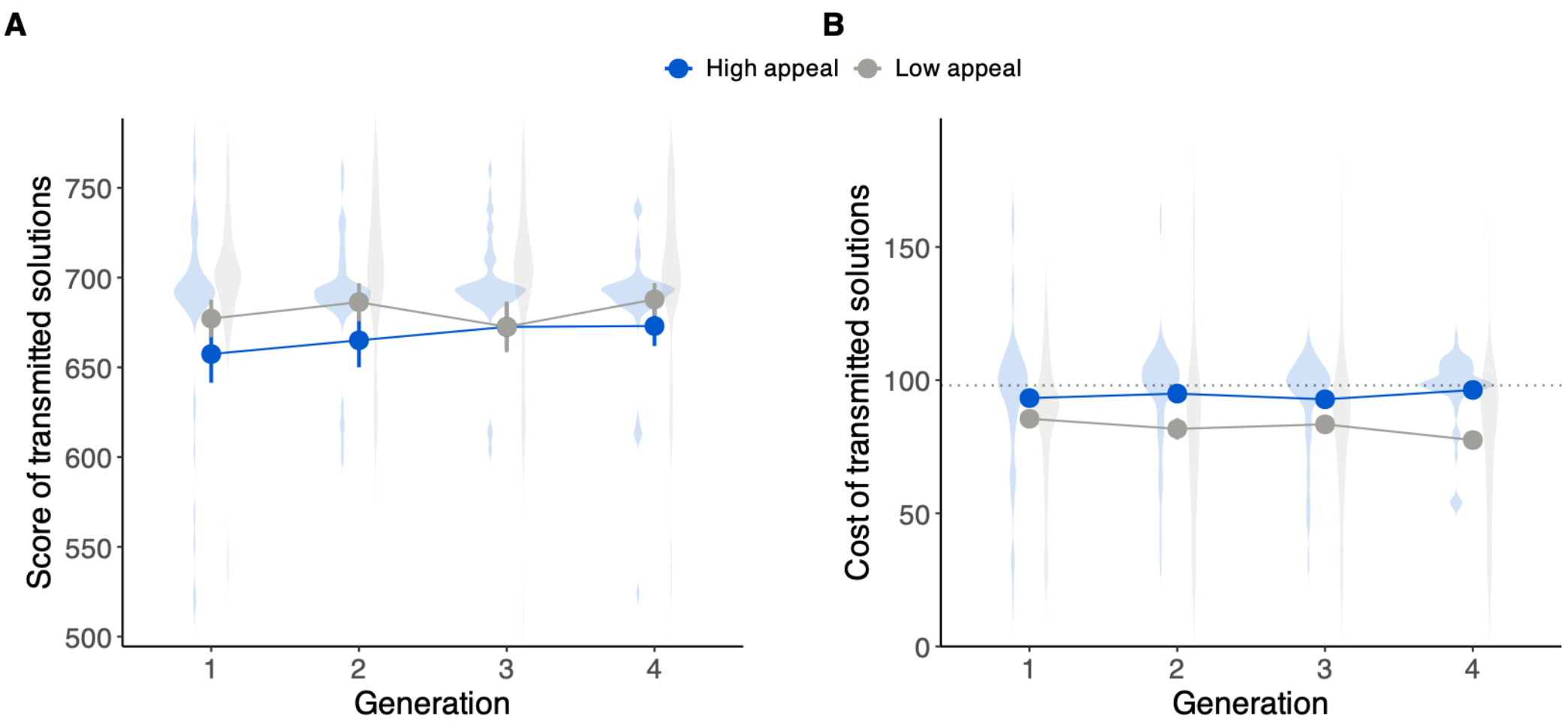

Supplementary Fig. 15. Study without demonstrator payoff information: Score and cost of culturally transmitted solutions across generations. A) In the low-appeal treatment (gray line), participants showed gradual improvements in solution scores across generations, whereas scores in the high-appeal treatment (blue line) did not change significantly. B) Participants in the low-appeal treatment progressively reduced the cost of their solutions, while those in the high-appeal treatment showed no statistically significant change over time. The dotted line represents the cost of the mix-and-match solution (see Figure 5 for an illustration). Blue indicates the high-appeal treatment condition, whereas grey indicates the low-appeal treatment condition.

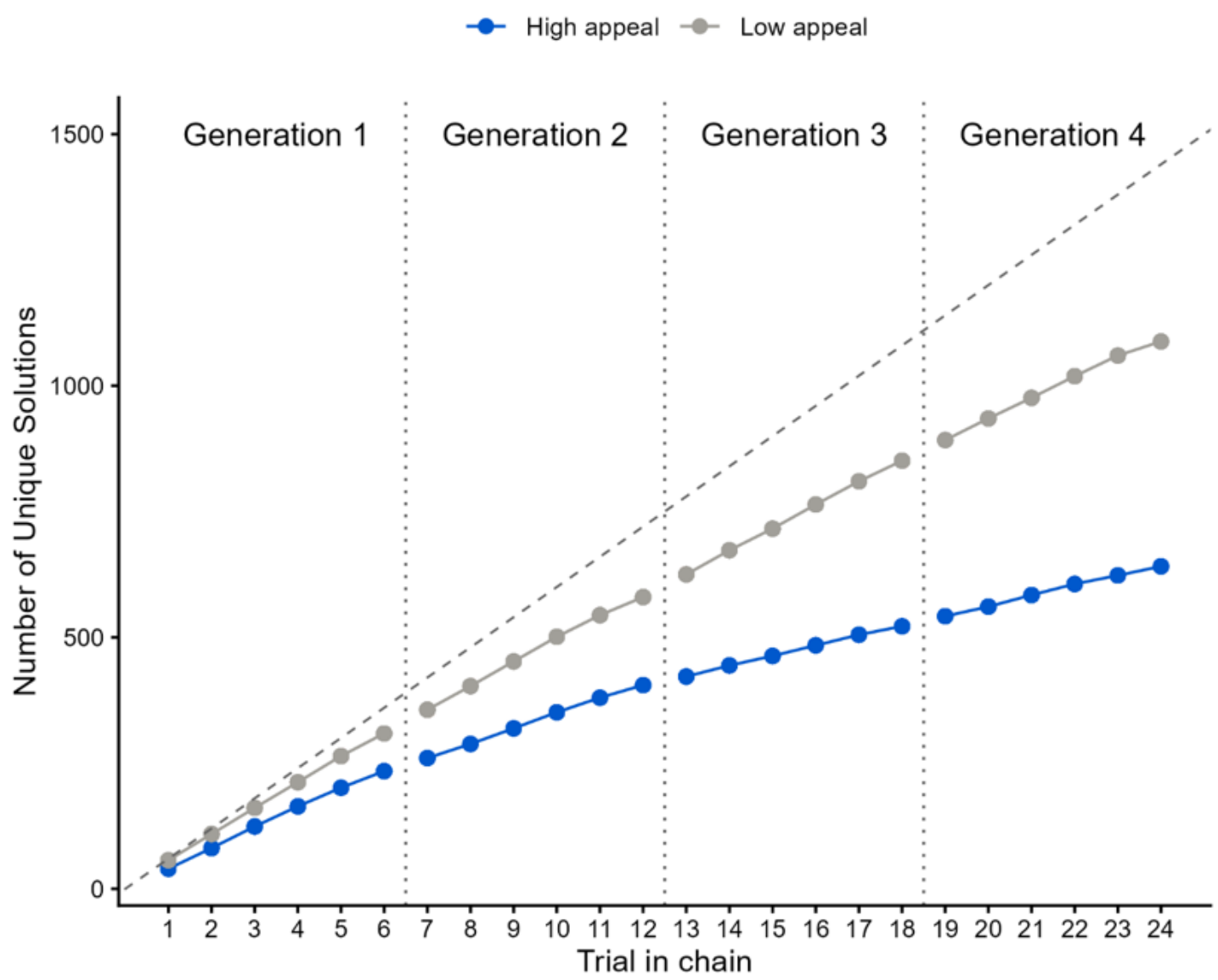

Supplementary Fig. 16. Study without demonstrator payoff information: Exploration across trials. Cumulative number of unique solutions (i.e., nutrient-to-plant allocation patterns) across trials in each treatment. The dashed black line indicates the theoretical upper bound of exploration, assuming every participant submitted a solution not previously produced in this treatment. The gray line shows the cumulative number of unique solutions observed in the low-appeal treatment, while the blue line shows the high-appeal treatment. Blue indicates the high-appeal treatment condition, whereas grey indicates the low-appeal treatment condition.

Term	Estimate (b)	SE	95% CI	df	t	p
**A Scores**						
Low-appeal only (n=240, num. chains = 20)						
(Intercept)	678.23	9.67	[659.28, 697.18]	98.59	70.13	<0.001
Generation	1.83	4.93	[-7.84, 11.50]	220.00	0.37	0.711
High-appeal only (n=240, num. chains = 20)						
(Intercept)	658.85	11.51	[636.29, 681.42]	240.00	57.23	<0.001
Generation	5.44	6.15	[-6.62, 17.50]	240.00	0.88	0.378
Pooled (n=480, num. chains = 40)						
(Intercept)	658.85	10.58	[638.12, 679.58]	229.37	62.30	<0.001
Low-Appeal	19.38	14.96	[-9.94, 48.69]	229.37	1.30	0.196
Generation	5.44	5.59	[-5.51, 16.39]	440.00	0.97	0.331
Low-Appeal:Generation	-3.61	7.90	[-19.09, 11.88]	440.00	-0.46	0.648
**B Cost**						
Low-appeal only (n=240, num. chains = 20)						
(Intercept)	85.37	3.55	[78.41, 92.33]	47.72	24.05	<0.001
Generation	-2.23	1.42	[-5.02, 0.56]	220.00	-1.57	0.119
High-appeal only (n=240, num. chains = 20)						
(Intercept)	93.28	2.30	[88.77, 97.79]	79.99	40.56	<0.001
Generation	0.70	1.11	[-1.48, 2.88]	220.00	0.63	0.532
Pooled (n=480, num. chains = 40)						
(Intercept)	93.28	2.99	[87.42, 99.14]	110.18	31.19	<0.001
Low-Appeal	-7.91	4.23	[-16.20, 0.38]	110.18	-1.87	0.064
Generation	0.70	1.28	[-1.81, 3.20]	440.00	0.55	0.586
Low-Appeal:Generation	-2.93	1.81	[-6.47, 0.61]	440.00	-1.62	0.106

Supplementary Table 7. Study without demonstrator payoff information: linear mixed-effects model estimates for expected scores (A) and costs (B) of culturally transmitted solutions across treatments. Separate models were fit for the low-appeal condition, the high-appeal condition, and pooled data to compare changes over generations. To account for the nested data structure, models included random intercepts for transmission chain. Fixed effects were tested using two-sided Wald t-tests with Satterthwaite-approximated degrees of freedom, and uncertainty is reported as 95% Wald confidence intervals. Model baselines: Generation=1, Treatment: High-Appeal (pooled-model)

Experimental Instructions and Task Screenshots

Instructions and control questions

1/12

Welcome to our study

Over the next few slides, we will explain the rules of the game and what you must do to maximize your monetary reward. Read them carefully so that you can earn as much bonus payment as possible.

Next

High-Appeal

2/12

Principle of the game

Your goal is to maximize the output of a field of 9 plants across 6 rounds.

Plants come in a variety of forms and colors and can be fed using 3 distinct nutrients.

The way you feed your plants affects the output of your field. However, we will not tell you how best to feed your plants. This is for you to figure out.

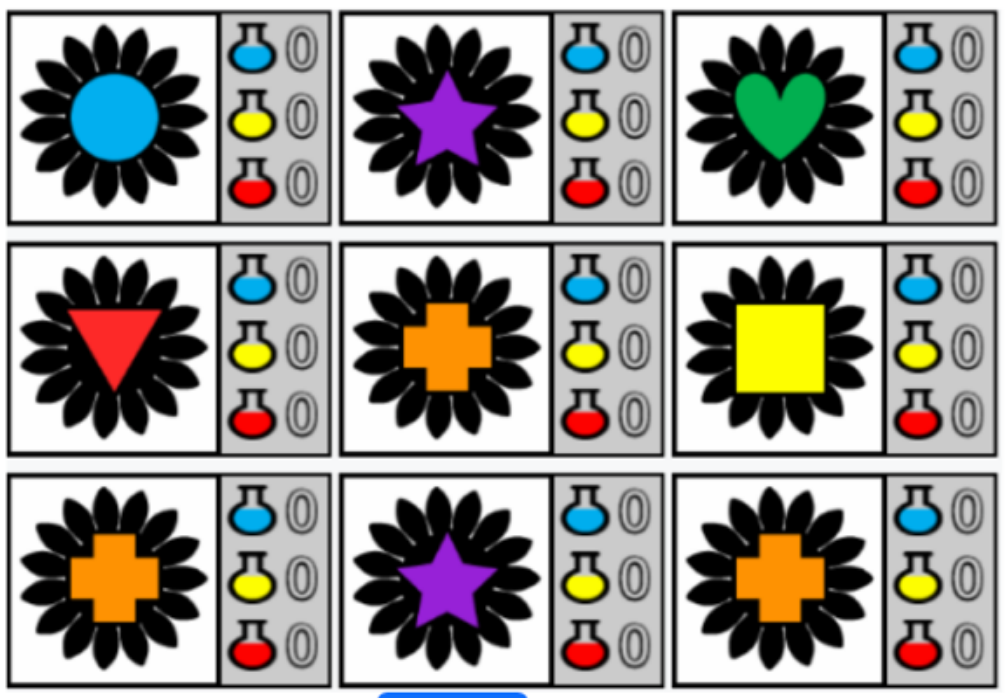

Previous

Next

Low-Appeal

2/12

Principle of the game

Your goal is to maximize the output of a field of 9 plants across 6 rounds.

Plants come in a variety of forms and can be fed using 3 distinct nutrients.

The way you feed your plants affects the output of your field. However, we will not tell you how best to feed your plants. This is for you to figure out.

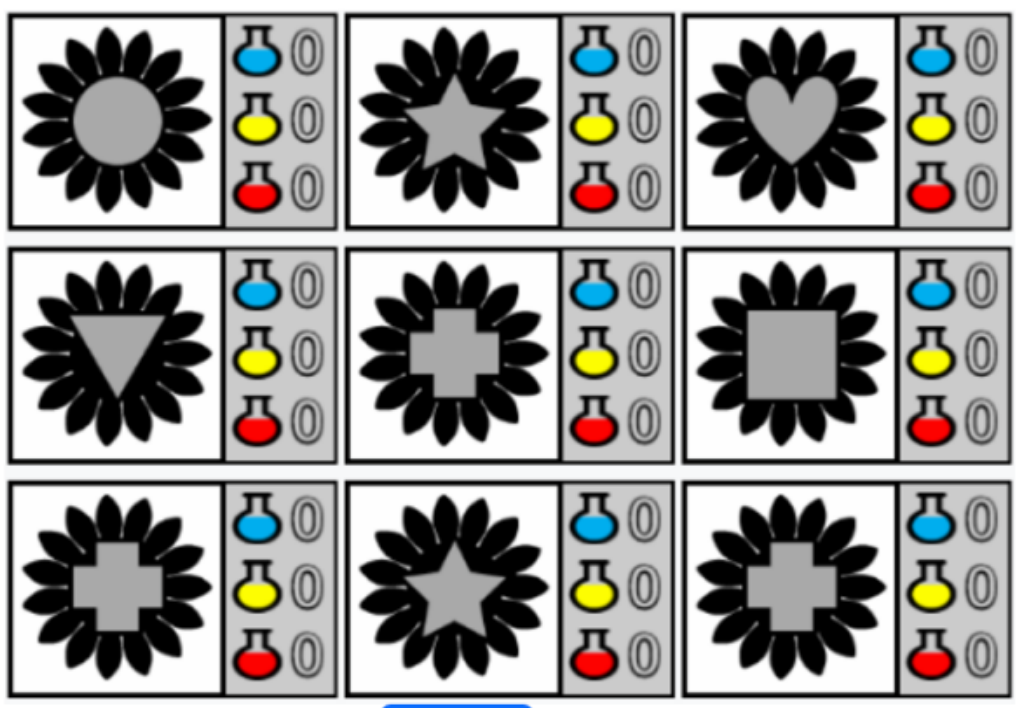

Previous

Next

High-Appeal

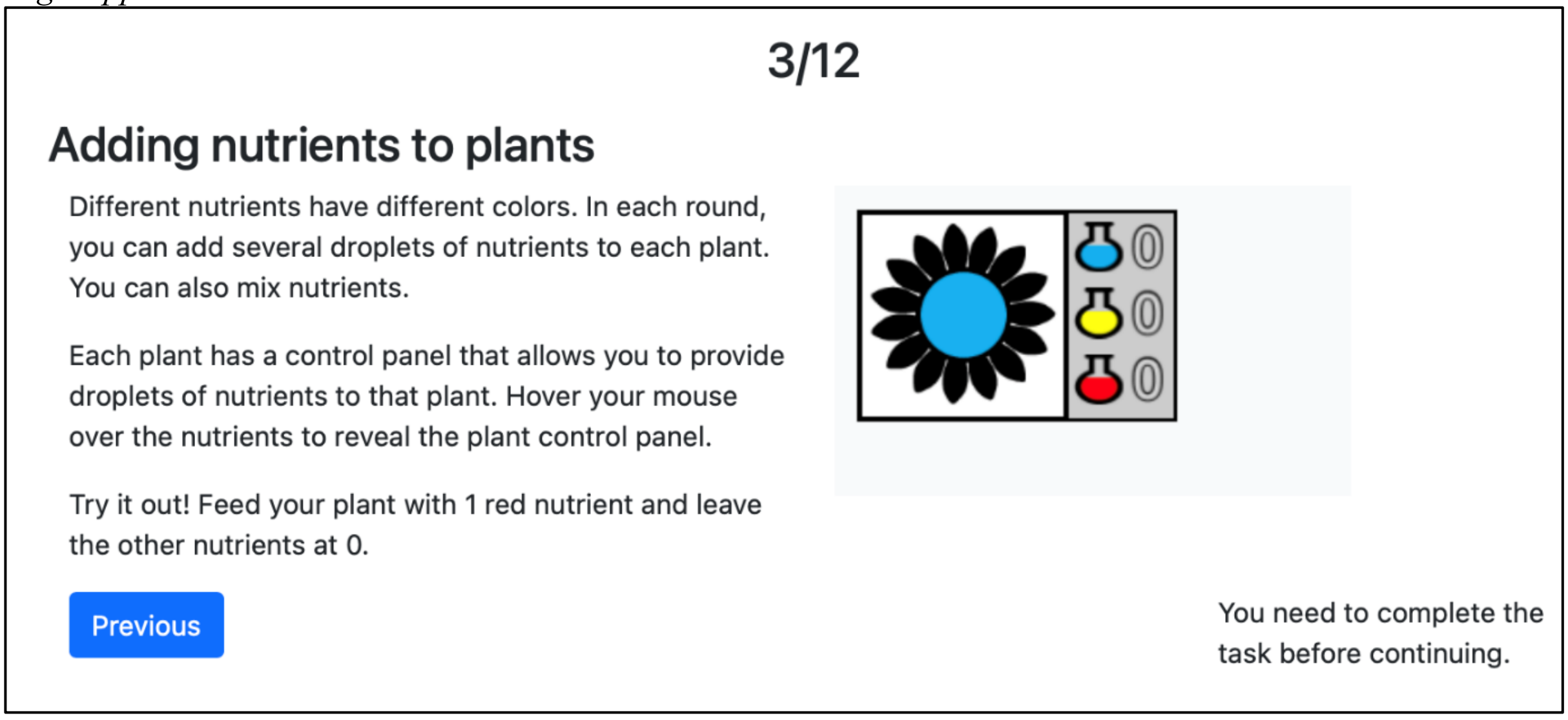

Low-Appeal

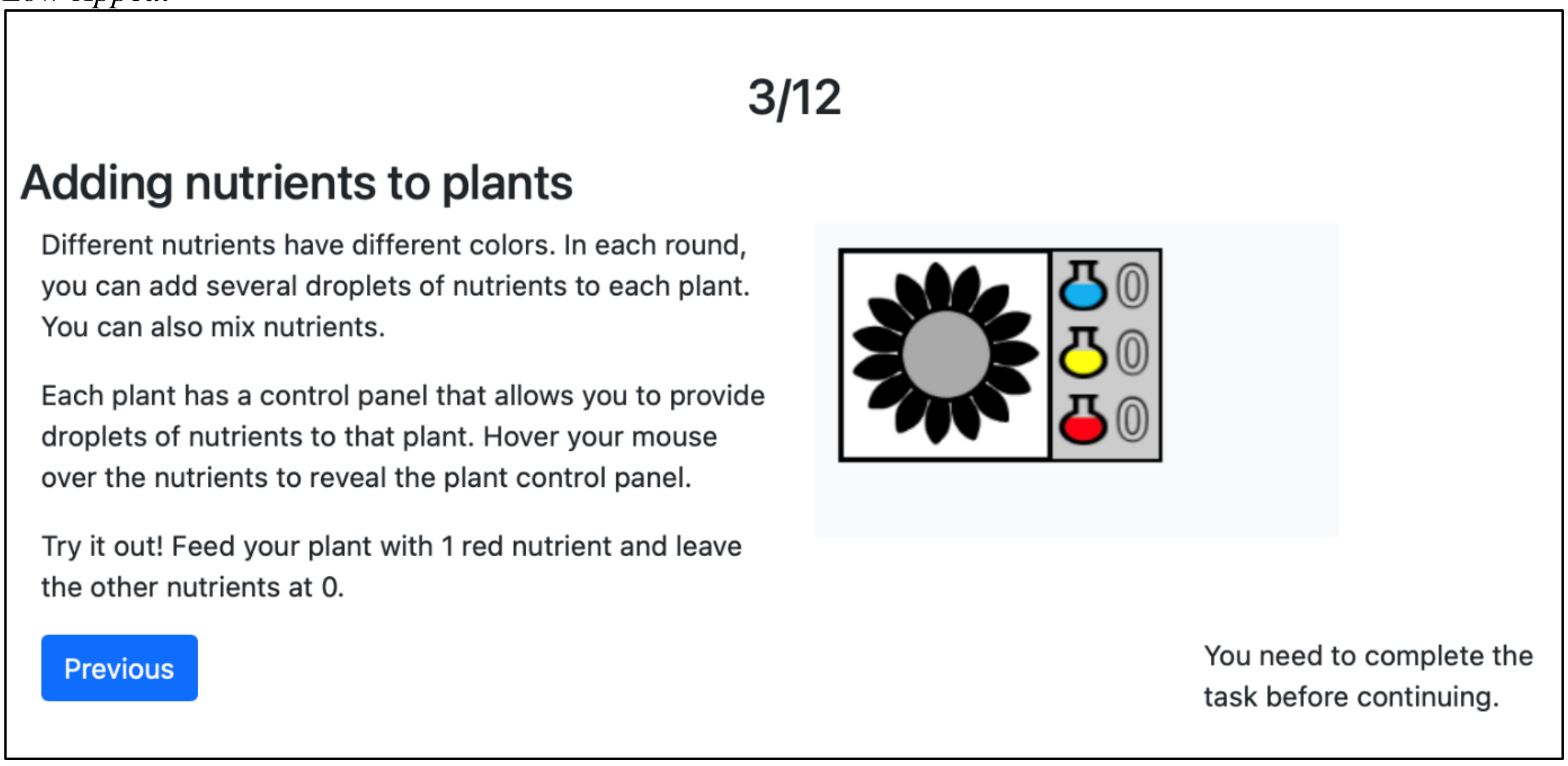

High-Appeal

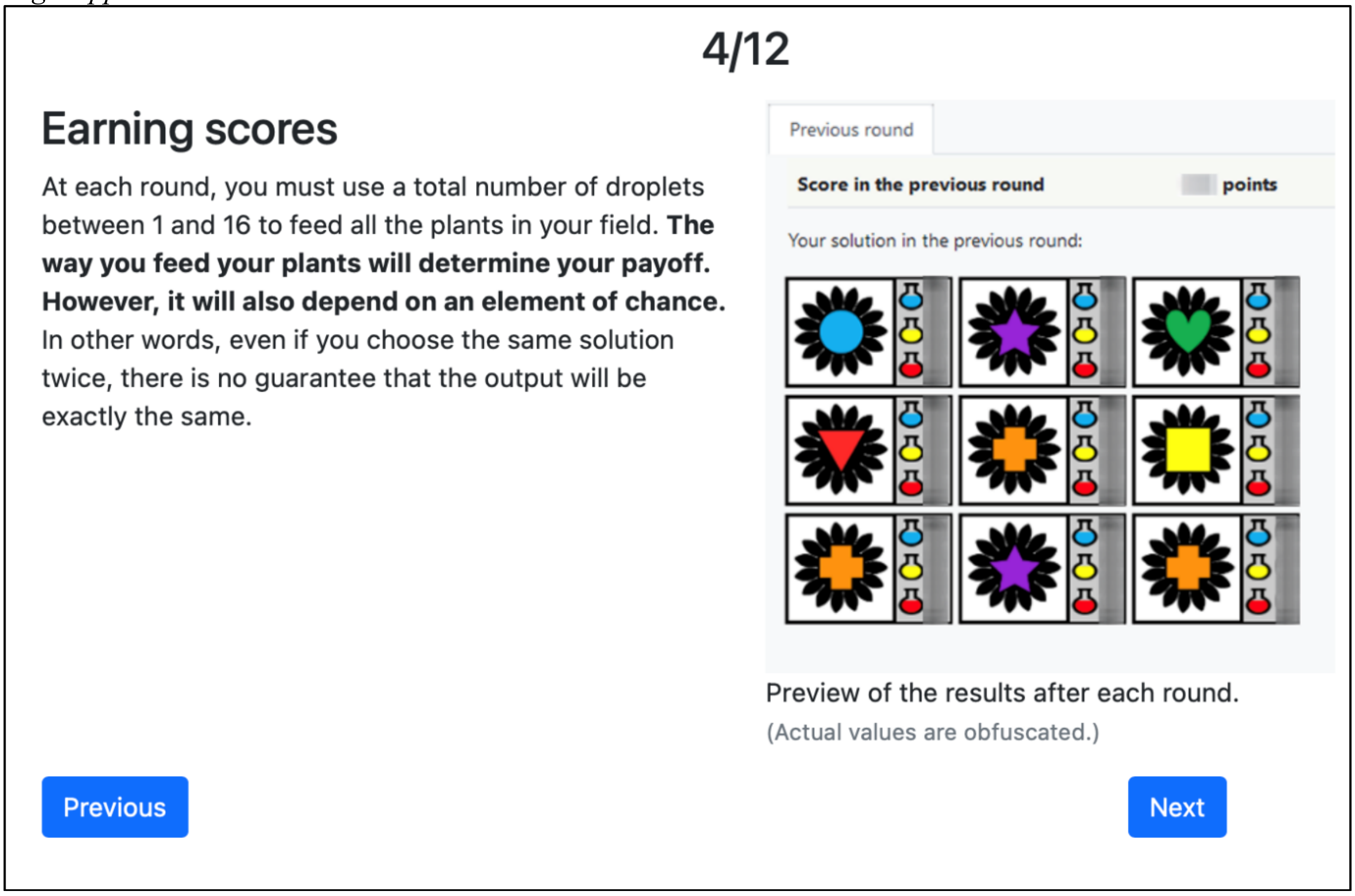

Low-Appeal

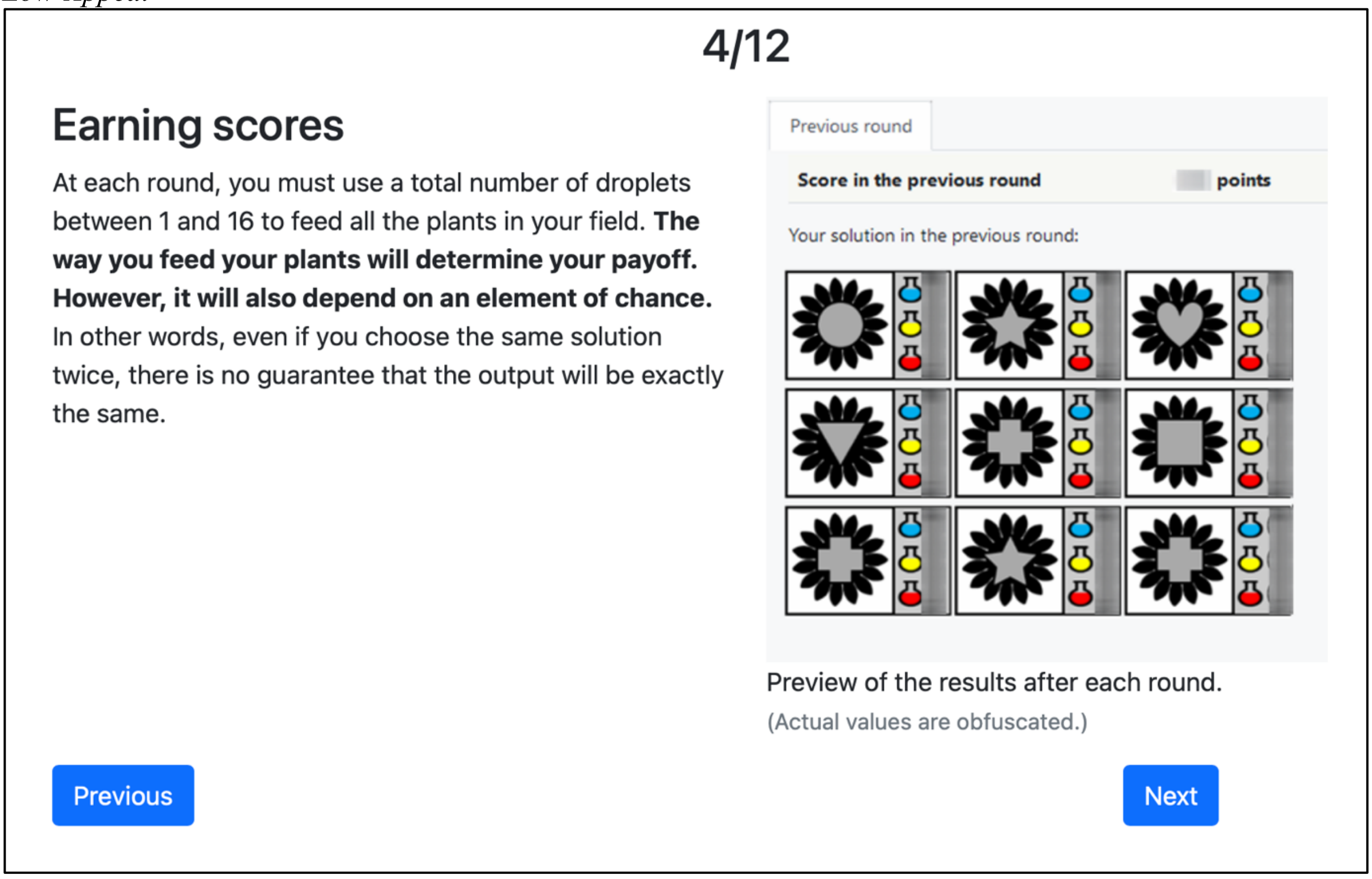

5/12

Please answer the questions based on the provided information. You are allowed to go back and re-read the information.

What factors determine your score in each round?

The way the field has been fed

An element of chance

Both of the above

Previous

You need to complete the task before continuing.

6/12

Please answer the questions based on the provided information. You are allowed to go back and re-read the information.

How many nutrients can you use in total in each round?

Any number

Any number from 1 to 16

Exactly 16

Previous

You need to complete the task before continuing.

7/12

Collective task and social information

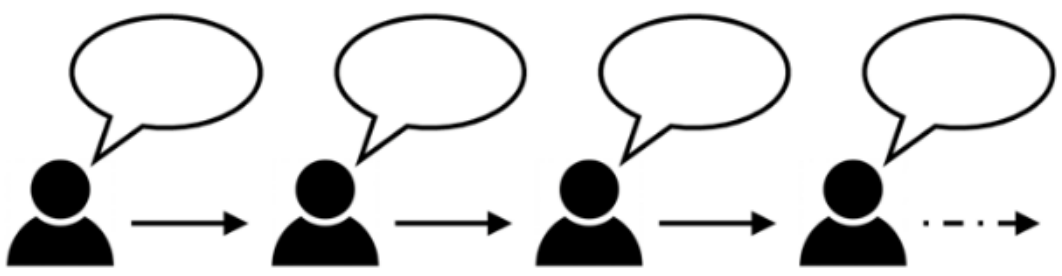

Improving the output of the field is a collective task. Although you are playing alone, you are part of a chain of several players, each of whom also has 6 rounds to improve their solution.

You are the first player in the chain. In the last round, we will ask you to write a piece of advice for the next player and share your solution.

The number of players in a chain is not disclosed. So, there might be, or might not be, a player after you.

Previous

Next

High-Appeal, First generation

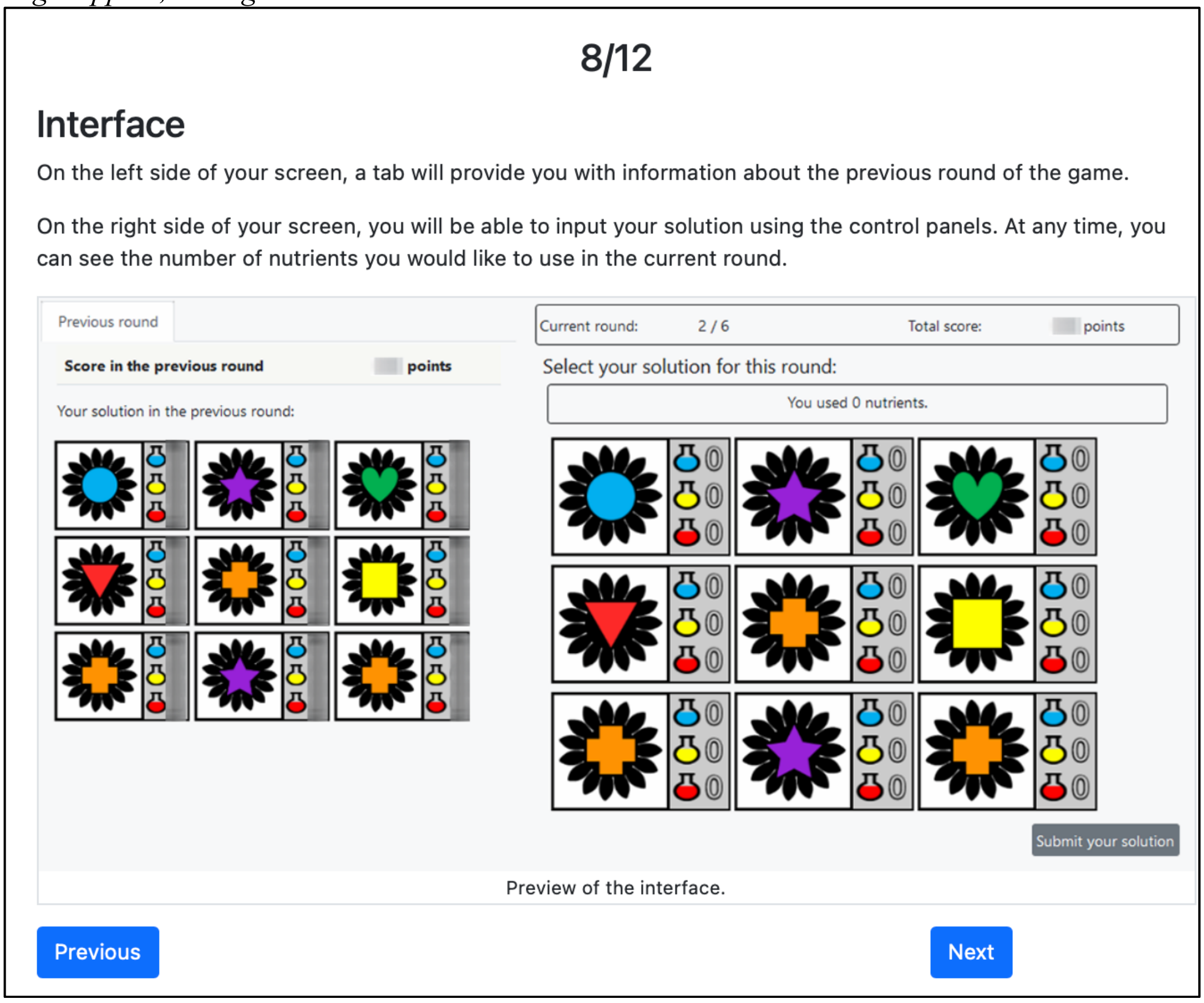

Low-Appeal, First generation

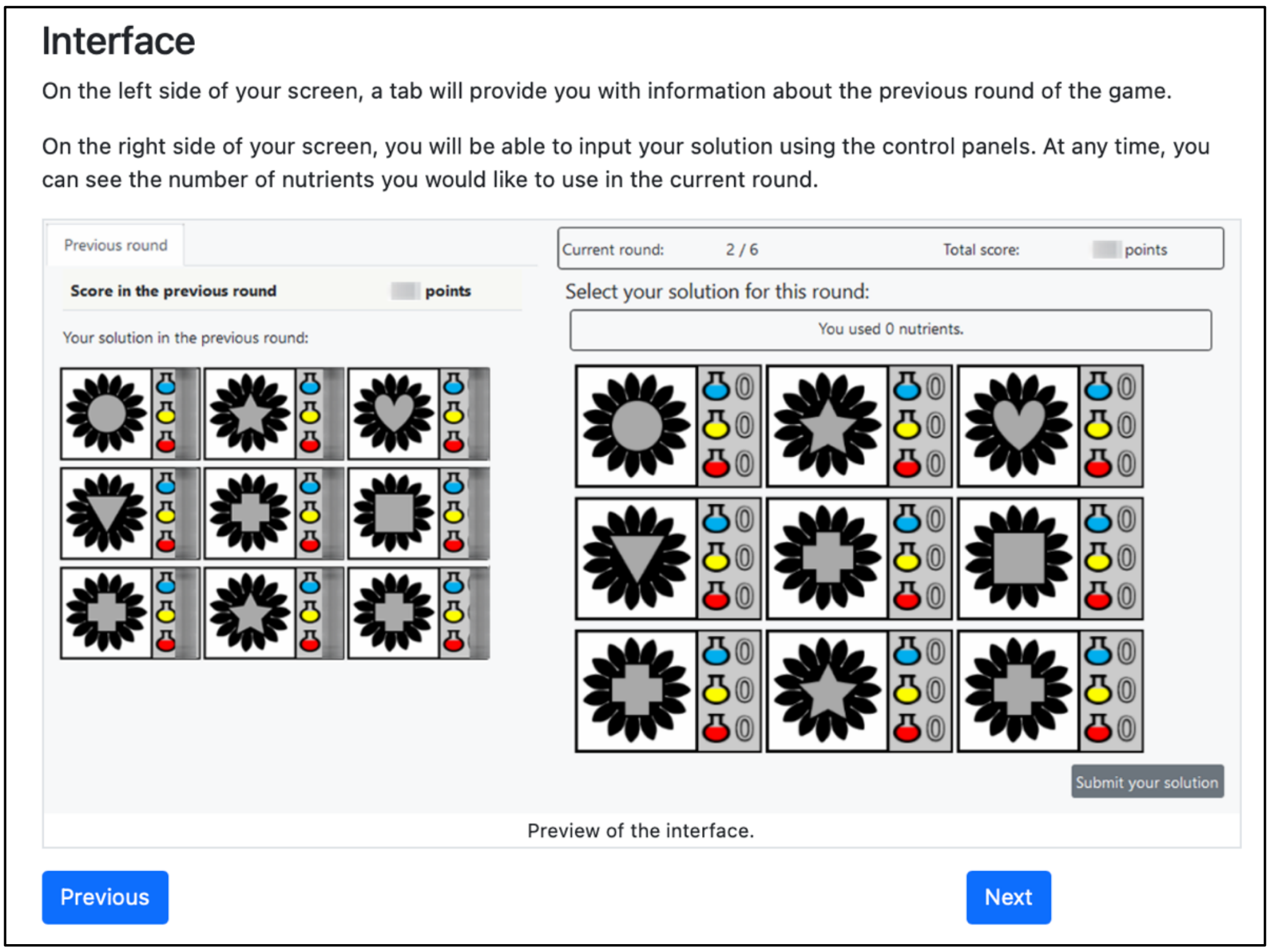

Low-Appeal, 2nd-4th generation

8/12

Interface

On the left side of your screen, a tab will provide you with information about the previous round of the game. In a second tab, you will see the advice and solution from a previous player.

On the right side of your screen, you will be able to input your solution using the control panels. At any time, you can see the number of nutrients you would like to use in the current round.

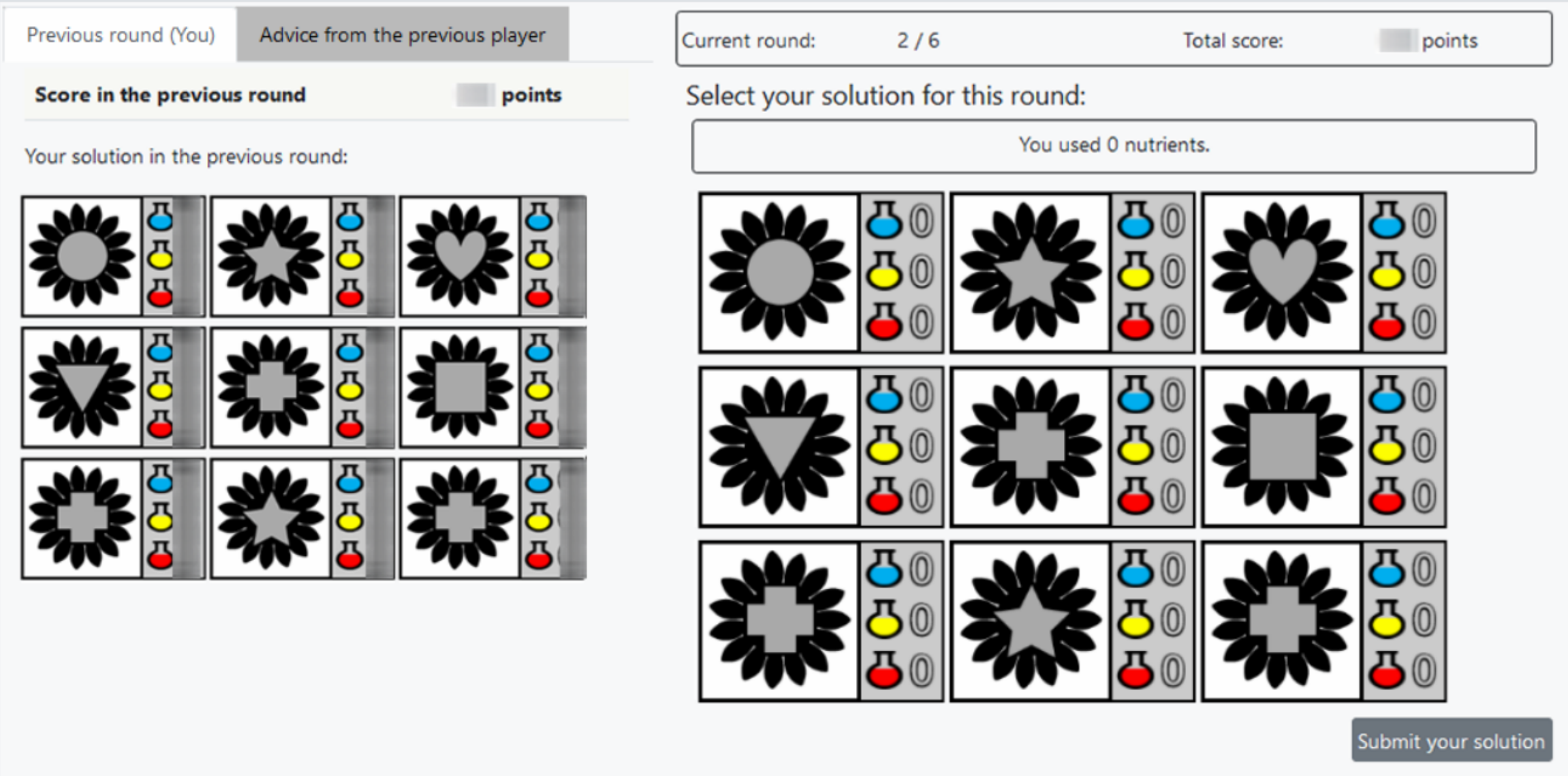

Preview of the interface.

Previous

Next

9/12

Monetary reward

Your reward will depend on how well you did in the game and how well a future player does in their first round. We will add up the points you obtained in each round. Then, we will add the points earned by the next player in their first round to your points (unless you are the last participant in the chain).

We will then convert those points into a monetary reward in GBP. The conversion rate is:

2500 points = £1.

This reward will be added to your participation fee on Prolific as a bonus payment.

Previous

Next

10/12

Please answer the questions based on the provided information. You are allowed to go back and re-read the information.

How is your bonus payment calculated?

It will be based only on my own score.

It will be based on the score of the next player in the chain.

It will be based on my own score and the score of the next player in the first round.

Previous

You need to complete the task before continuing.

11/12

Please answer the questions based on the provided information. You are allowed to go back and re-read the information.

What is the equivalent of 2500 points in pounds?

£1

£1.5

£0.5

Previous

You need to complete the task before continuing.

12/12

You have finished the instructions. You won't be able to go back to them once you start the experiment, so please make sure you have understood everything before starting and go through the instructions again if you feel you need to.

To start the experiment, click on the "Start Experiment" button below.

Previous

Start Experiment

Screenshots from the gameplay

Task Screen, Low-Appeal, First generation

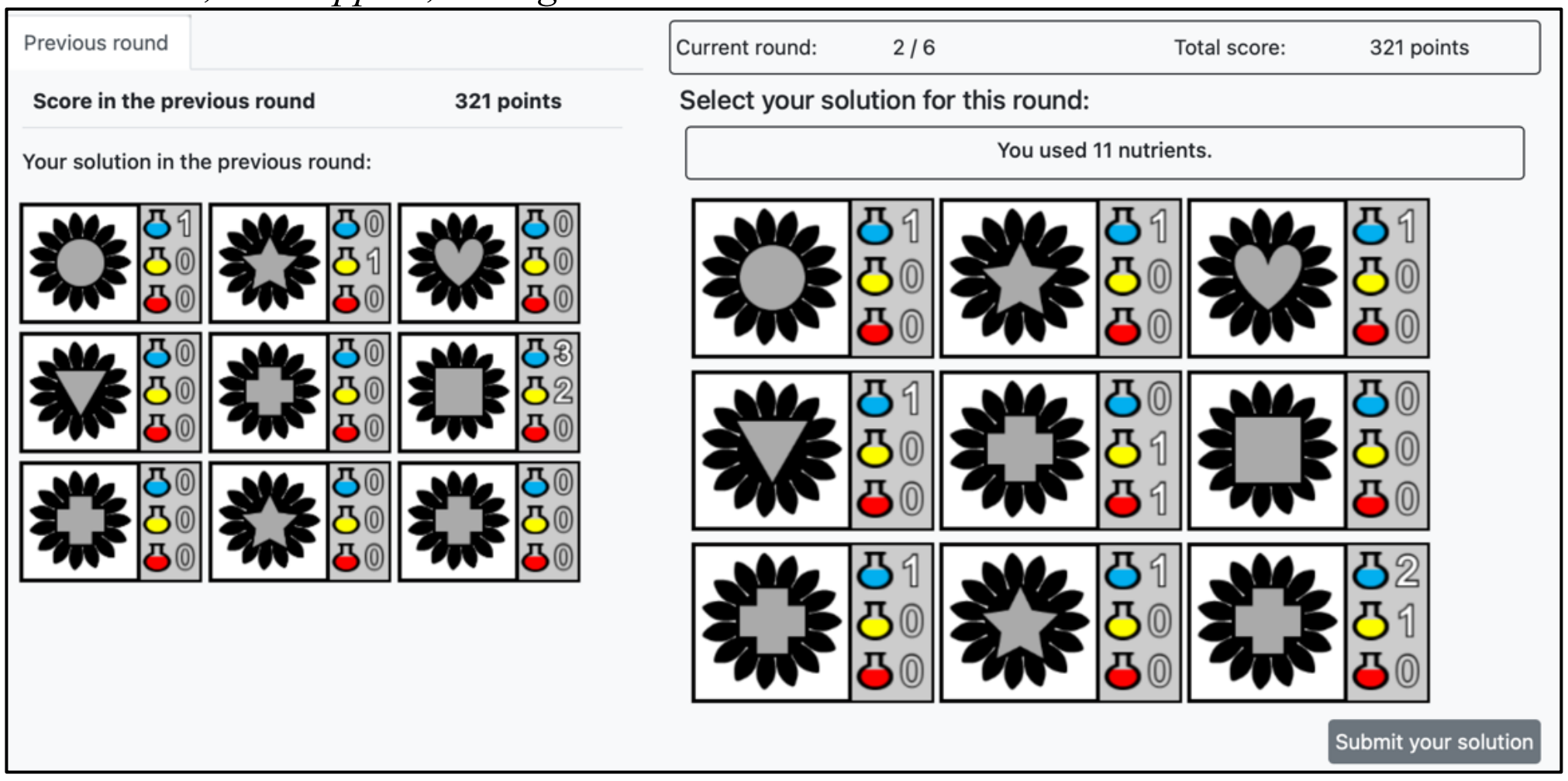

Task screen, Last round, High-Appeal, First generation

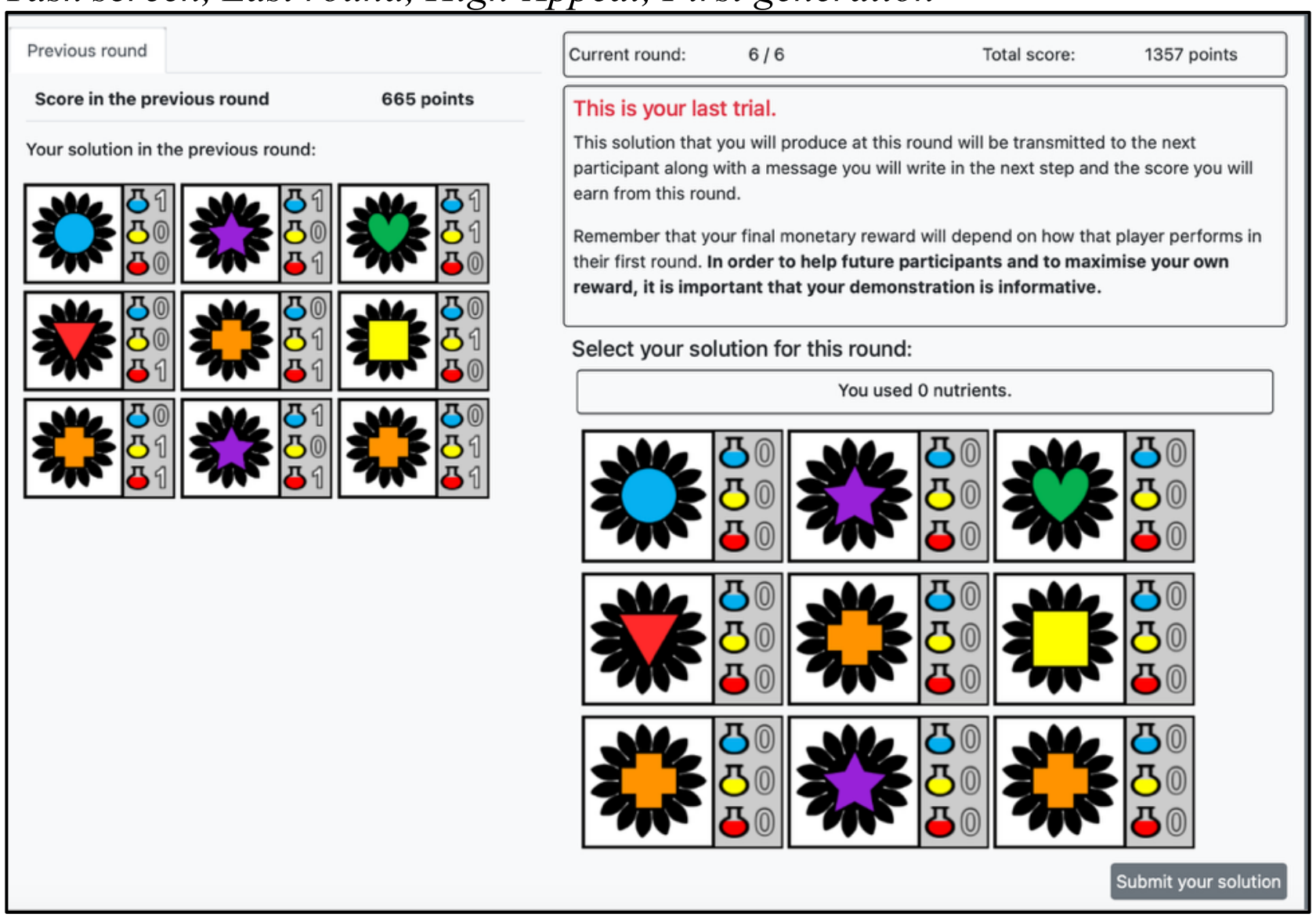

Advice Selection Screen, Low-Appeal, 2nd-4th generation

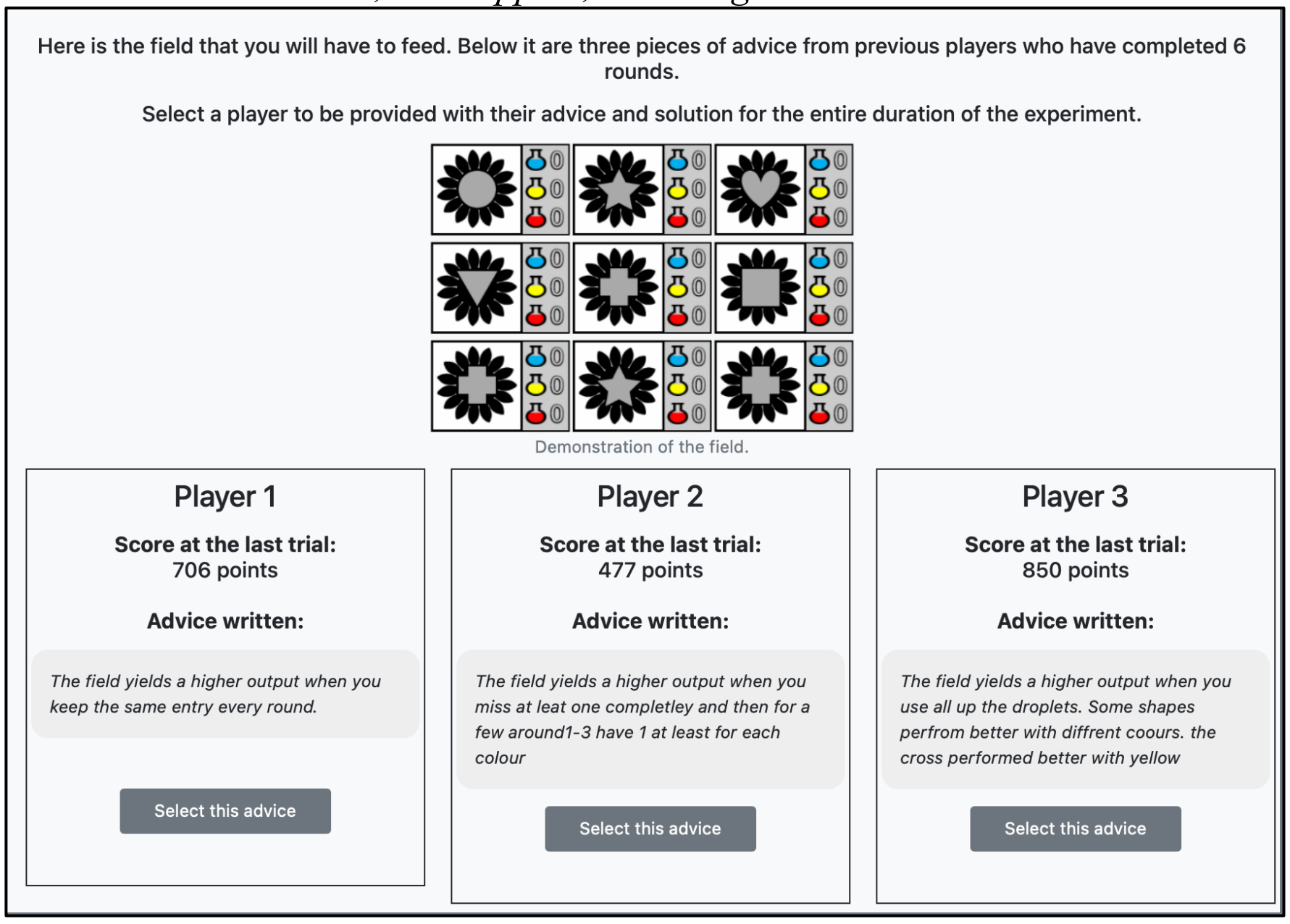

Task Screen, Low-Appeal, 2nd-4th generation

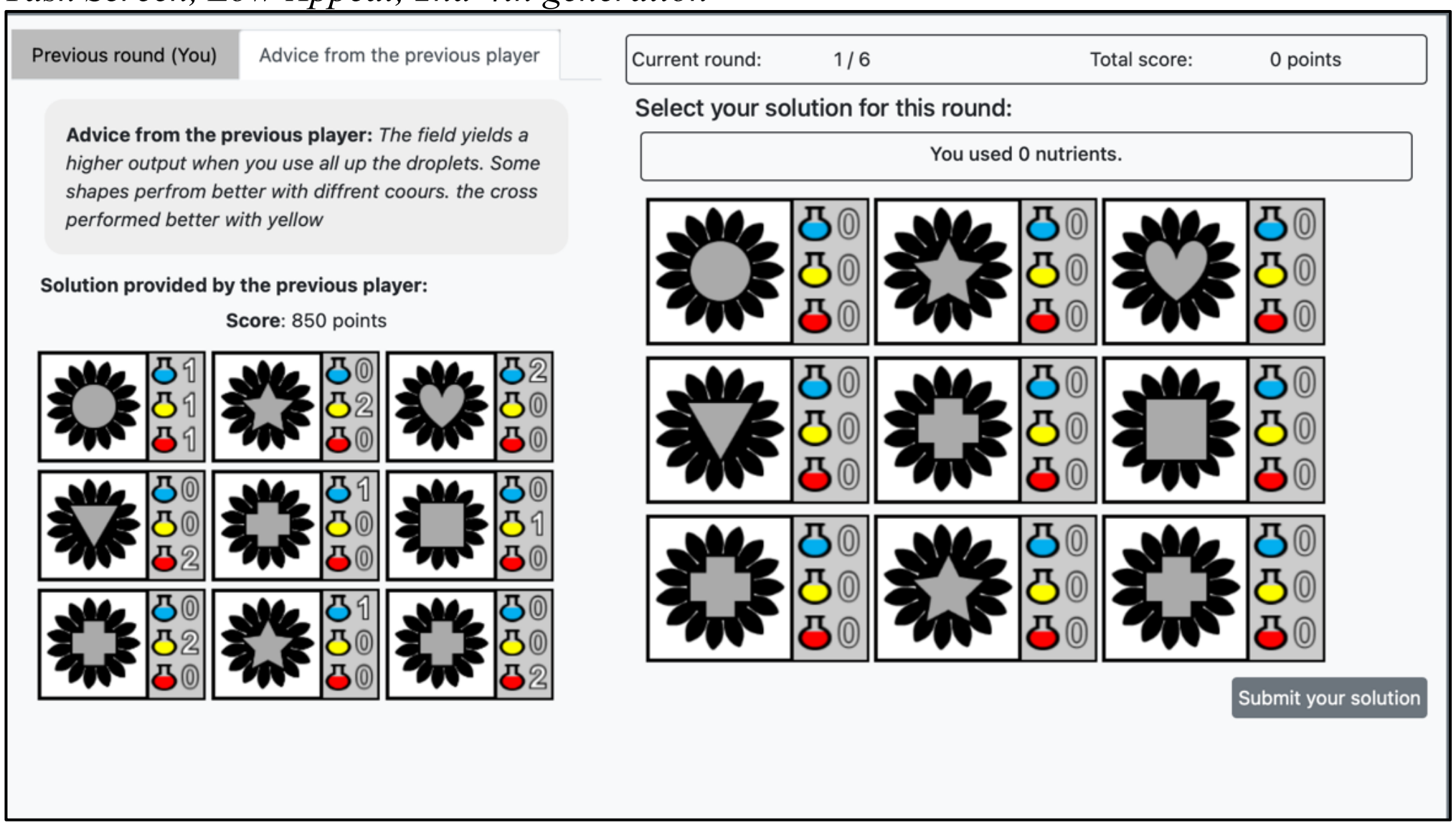

Task Screen, Low-Appeal, 2nd-4th generation

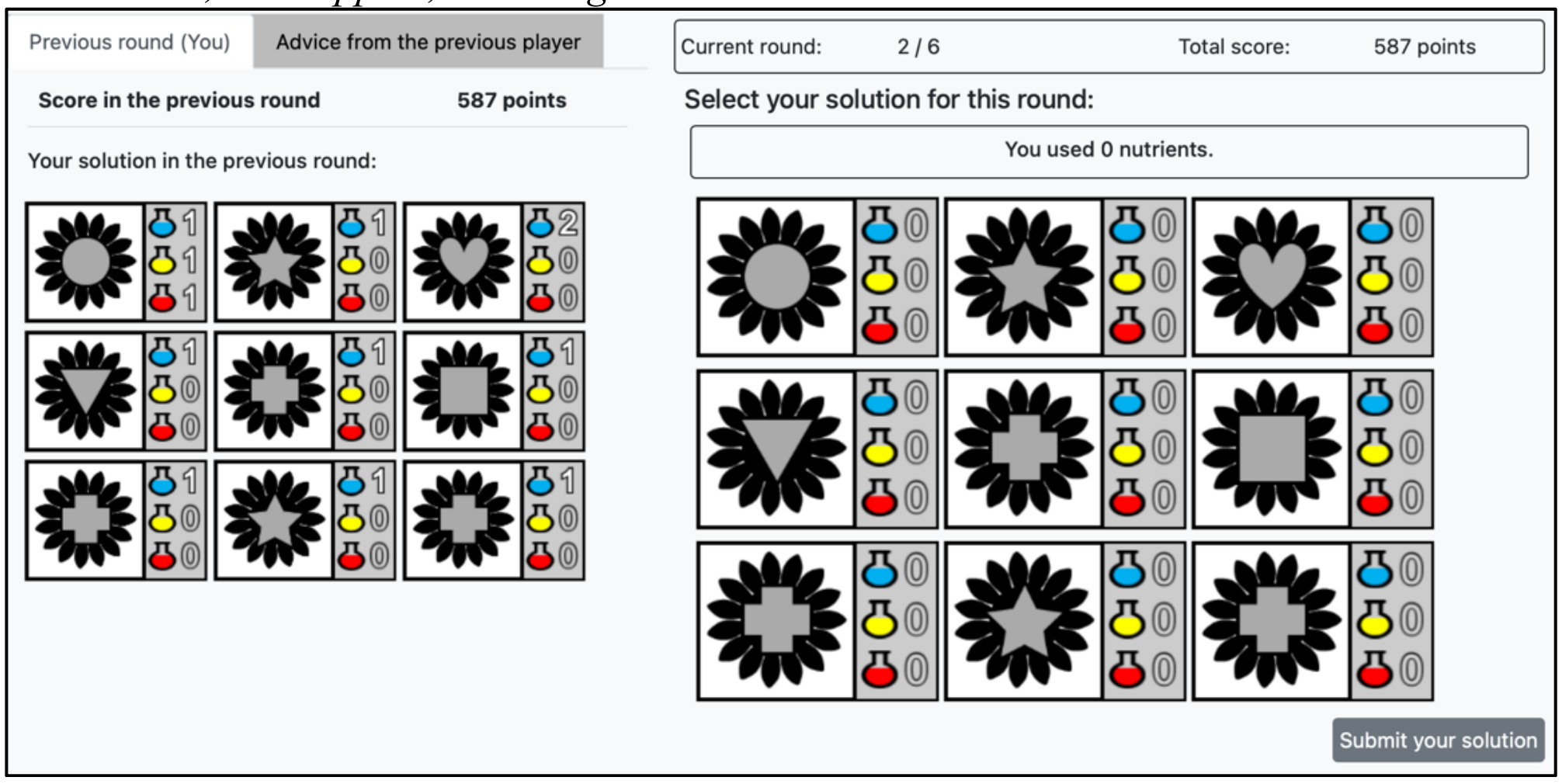

Advice Message Screen, High-Appeal

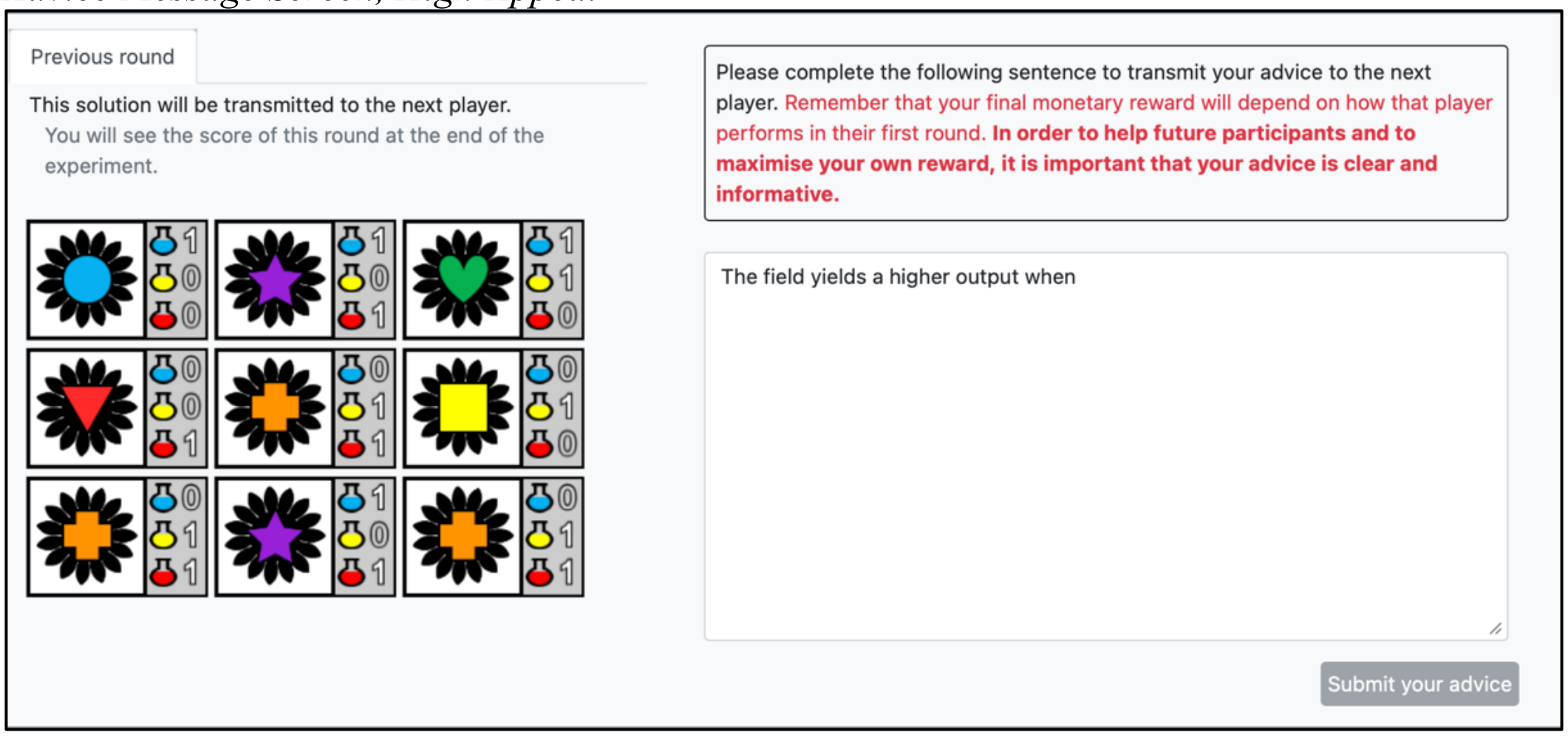

Supplement: Supplementary file 1 [file 41467_2026_77677_MOESM1_ESM.pdf]
