# Supplementary material for: Cognitive appeal promotes the persistence of inefficient solutions and hinders cumulative cultural evolution
Source: Nat Commun. 2026 Sep 12;17:10752. doi: 10.1038/s41467-026-77677-9 (PMC13661520; doi:10.1038/s41467-026-77677-9)
Supplement: Supplementary file 2 [file 41467_2026_77677_MOESM2_ESM.pdf]

nature portfolio

Peer Review File

Cognitive appeal promotes the persistence of inefficient solutions and hinders cumulative cultural evolution

Corresponding Author: Dr Maxime Derex

This file contains all reviewer reports in order by version, followed by all author rebuttals in order by version.

Attachments originally included by the reviewers as part of their assessment can be found at the end of this file.

Version 0:

Reviewer comments:

Reviewer #1

(Remarks to the Author)
see attached

Reviewer #2

(Remarks to the Author)
This study examines the impact of visual appeal on the efficient transmission of solutions. Examining how different kinds of information/constraints/payoffs impact cultural transmission is relevant to core questions in cultural evolution. The experiments is well designed to explore the effects of an aspect of visual appeal on cultural transmission, albeit with the limitations of experiments of this kind. The methods are clear with adequate detail.

The data are also interesting, but the paper requires revision for the following reason(s). There is a mismatch between the more narrow and careful framing of the results in some of the narrative of the discussion and the overly general claim that these data may explain the persistence of ineffective cultural technologies-shamanism, bloodletting etc.

I suggest more discussion of perceptual interference-which I think the data speak directly to, and less discussion about massively more complex cultural phenomena like magic. The low vs high appeal framing could mean many things-I suggest a more specific and accurate labeling of the experimental manipulation. I also suggest more reflection on mechanisms of perceptual processing underlying the effects of condition on the efficient transmission of solutions.

Reviewer #3

(Remarks to the Author)
This manuscript describes and explores an interesting experimental design to investigate the ``law of similarity” found in a variety of anthropological case studies. Using what the authors call subjective appeal, they investigate whether the degree of visual similarity between features of plants and features of nutrients determines the effectiveness of the solutions. They conclude that their experimental design can successfully replicate situations seen in the ``real world” where inefficient but matching solutions persist.

Is this a fair assessment or do the authors propose subjective appeal to be the cognitive bias that generates the mix and match pattern? If so, the cognitive underpinnings of subjective appeal should be better explained so that the contribution of the experiment beyond replicating the “real-world” pattern (which is great!) becomes more obvious.

I interpreted Figure 1 as proof that the experiment is carefully designed to generate situations where an ineffective but ``matching” solution (similar to the observed resemblance between herbs and body parts) can persist, and the authors are now in the position to explore why this is the case. However, I was left disappointed by this analysis. The experiment has generated such a rich data set, which seems underexplored, apart from looking at the number of unique solutions and the structure of the most common solution.

Regarding the most common solution, the authors say that the most common solution in the low appeal treatment accounted for 3% of the sixth-trial responses. What does this mean? There are 20 chains, and in each chain there are 4 sixth trials. 3% of 80 is 2.4 individuals (should it not be an integer?). So the most common solution has been discovered by 2 individuals? In the high appeal group 16% of 80 (12.8) individuals show the same solution. This is interesting but is this from individuals at the beginning of the chain or at the end? In any case, the authors contrast the most common solution as if they were representative of the different treatments, which they are not — especially not in the low appeal group, given the very low numbers of individuals having discovered them. One needs to quantify the variation in the solutions produced by both treatments possibly along at least three dimensions contributing to the theoretically most efficient solution: number of plants used, number of blue treatments and number of treatments per plant.

Further, in Figure 4 net payoff is only the average net payoff as the payoffs are uniformly distributed across the intervals [580,940] and [514,874]. From these uniform distributions, it is also obvious how much the payoff values overlap and that there is a not small probability that the solution labelled as insufficient actually provides a payoff higher than the one of the efficient solutions. Do those large variations in the payoff, in part, drive the result? Can the authors motivate their choice of the payoff function — it seems unusual that the “more right” the solution is (the more plants are included), the higher is the variation, i.e. the harder is it to distinguish between ``inefficient” and ``efficient” solutions based on a very small number of trials.

The authors state that in both treatments, payoff-biased social learning is used equally. However, what is shown is that the individuals choose the best of the three pieces of information and not whether they indeed use this solution. Can you provide more details on how individuals incorporate the ``learnt” solution in their first try, and so quantify the extent to which social information is used? Currently, the analysis does not tell whether both treatments use social learning to the same extent.

What is the role of the written advice? It may provide interesting information about how individuals reconstruct the payoff function. Why is it not analysed?

The authors state that the low appeal treatments each generation was associated with a 17 point increase but Figure 1 indicates that the mean payoff stays at best constant between generation 3 and 4. Could you clarify? Again, it would be very illuminating if the authors would analyse the diversity in more detail, for exampl,e through exploring development of different parts of the solution within the chains (for example, what do you discover first and when: number of plants, the colour of the nutrient or the number of nutrients per plant). The data set is so rich and provides so much information that can be used to generate a better understanding of why we see what we are seeing. At the moment, the analysis does not do the experiment justice. Also, it would be interesting to see the results of the individual chains in the SI to gain an understanding of the variation within and between the chains.

In the conclusion, the authors state that the experiment uncovered the association between visual similarity and functional relationship. In my view, this statement is not supported by the current analysis. If you would take the first individuals in each chain and look at their first solutions. There is no payoff information yet. So if those first solutions are similar to the solutions produced later in the chain, then the functional argument does not hold. The matching of colour may simply be a cognitive attractor (in the absence of any other information), which is hard to overcome, in part due to the high variation in payoff, making it hard to identify a more efficient solution from a small number of trials.

Version 1:

Reviewer comments:

Reviewer #1

(Remarks to the Author)
I appreciate the authors' revision in addression reviewers' concerns. I am satisfied with the revisions as well as the the additional analyses, and recommend acceptance of the manuscript.

I sign my reviews,
Kevin Hong

Reviewer #2

(Remarks to the Author)
The authors have been responsive to reviewer feedback and the paper is improved as a result.

Reviewer #3

(Remarks to the Author)
I thank the authors for their thoughtful replies to my comments and also for clearing up misunderstandings on my part. I

appreciate that they did extra analysis to investigate some of my questions. I find the paper much improved and an interesting contribution to the literature on cumulative cultural evolution.

I am intrigued by some of the new results! Below are additional thoughts that may help strengthen the paper further. Given that we are in round 2, I offer these as suggestions rather than requirements. The authors should prioritise those they find most helpful, given the focus of the paper.

I would encourage the authors to have a more nuanced discussion about the social learning claim. You report the percentage of individuals choosing the role model with the best solution with roughly 75% but only 15% are using those solutions unmodified. Now it might be even expected that not the entire solution is copied and Fig. S3 in the SI shows that the differences between the transmitted and first solution are not too small. (Maybe I missed this but what is the solution vector? Is it the vector of 3x9 components describing the number of times red, blue, and yellow is used for every plant?). To make the claim that payoff-biased social learning is used stronger, it would be great to see what the groups actually learn/use from the transmitted solution (they need to learn something to claim social learning). Could you determine where the variation is? For example, does the low-appeal group learn to attend to all 9 plants and use dominantly blue nutrients, and therefore, we see variation mainly in the number of nutrients per plant? This may provide insights into which information is used from the available social information.

I really like Figure A1. Both groups infer that you have to attend to all plants. The low appeal group infers that blue nutrients should be used but both groups struggle with inferring that you should only provide one nutrient per plant. And this may not be too surprising as this fact may not be identifiable due to the large payoff variation. Just a minor point, could you add the error bars?

It's interesting that the most efficient solution has been repeatedly found in the low-appeal group but did not spread. I guess this is expected given the large payoff variation. The advantage of the best solution may only become apparent when this solution is used repeatedly (what indiviudals cannot do). Again, I was wondering whether there is something structurally similar about the “common” variants shown in Fig. 4, especially in the low-appeal group. For example, do they only differ in the number of nutrients per plant? Then one could say that the low-appeal group did actually discover what they could, given the large payoff variation.

I guess the same goes for progress within a generation. Which aspect of the solution are individuals varying or is there no structure to this variation?

line 191: missing table and figure numbers

I still find net-payoff in Fig. 5 misleading. Individuals almost never get this payoff when using this treatment. So either label it average payoff or provide the interval of variation.

Version 2:

Reviewer comments:

Reviewer #3

(Remarks to the Author)
I thank the authors for their revision and I am happy with the paper. It presents an interesting contribution to the cultural evolution literature.

Open Access This Peer Review File is licensed under a Creative Commons Attribution 4.0 International License, which permits use, sharing, adaptation, distribution and reproduction in any medium or format, as long as you give appropriate credit to the original author(s) and the source, provide a link to the Creative Commons license, and indicate if changes were made.
In cases where reviewers are anonymous, credit should be given to 'Anonymous Referee' and the source.
The images or other third party material in this Peer Review File are included in the article's Creative Commons license, unless indicated otherwise in a credit line to the material. If material is not included in the article's Creative Commons license and your intended use is not permitted by statutory regulation or exceeds the permitted use, you will need to obtain permission directly from the copyright holder.
To view a copy of this license, visit https://creativecommons.org/licenses/by/4.0/

REVIEWER COMMENTS

Reviewer #1:

This paper presents a pre-registered transmission-chain experiment examining how cognitive appeal can interfere with cumulative cultural evolution. Using a “plant-feeding” task, the authors manipulated whether plants had appealing visual correspondences with nutrient colors (high-appeal condition) or were neutral gray (low-appeal condition). Across four generations, participants in the low-appeal condition progressively converged on efficient, low-cost solutions, while participants in the high-appeal condition developed and culturally transmitted costly but ineffective “mix-and-match” strategies. The results suggest that subjective appeal—stemming from perceptual resemblance— can bias expectations of efficacy, reduce exploratory search, and lead to the persistence of inefficient solutions despite payoff-biased learning.

This is a neat paper that I think would make a contribution to the cumulative cultural evolution literature. I do have a few substantive comments and some specific suggestions which I hope would improve future versions of this manuscript.

Reply: We thank Reviewer 1 for their positive and constructive feedback. We respond point-by-point below. We believe that the additional analyses and clarifications requested by the reviewer have strengthened the manuscript and improved the presentation of our conclusions.

1) I was requested by the editor to provide specific comment as to any issues regarding the match between the pre-registrations and the reporting in the manuscript, so I’ll discuss this first. I think the overall matching between the preregistration and the reporting is fine, but there are two notable issues.

· In the pre-registration, the first hypothesis/prediction is that “Participants in the low-appeal treatment are more likely to choose the solution with the highest payoff among the three solutions they can choose from, compared to the high- appeal treatment”. The result as reported in the manuscript show no such difference: “No differences between treatments were observed in terms of social learning strategies. Participants in both treatments relied on payoff-biased social learning to choose from the three available cultural models, selecting the highest- scoring demonstrators 72% of the time in the low-appeal treatment and 78% of the time in the high-appeal treatment (ß= -0.06; 95% CI [-0.16, 0.05]; p = 0.31).” Given that this is a main hypothesis of the study, such discrepancy should be acknowledged and explicitly discussed. I suggest adding a few sentences before the “No difference between treatments were observed…” paragraph. As I’ll elaborate in a later comment, I think the lack of such predicted difference has important implications on the conclusions of the study.

Reply: We thank Reviewer 1 for raising this point. The revised manuscript now explicitly states the discrepancy between our preregistered expectations and what we observed (line 155). The revised manuscript also includes two additional paragraphs discussing the implications of participants’ reliance on payoff information (lines 272-289).

In the last part of the pre-registration, the authors proposed several additional variables to analyze (copy-pasted here):

- Cultural selection: Agreement among learners across generations and between treatments.

- Social Influence (1): Probability of copying the previous participant's solution at the first trial across generations and between treatments.

- Social Influence (2): Distance between inherited and transmitted solutions across generations and between treatments (calculated as the sum of the absolute differences between each nutrient for each plant)

- Individual exploration (1): Number of unique solutions across generations and between treatments.

- Individual exploration (2): Distance between each pair of solutions produced by the same individual across generations and between treatments. (calculated as the sum of the absolute differences between each nutrient for each plant)

- Cultural convergence: Within- and Between-chain cultural diversity of transmitted solutions across generations and between treatments.

- We will also compare the data to a previous study, where participants only had access to advice from previous participants before selecting a solution, as opposed to the current study, where they had access to both advice and the payoff from the last trial."

Among these, the present manuscript explicitly discussed "Individual exploration (1)". I assume that most of the other variables did not yield significant and/or interesting patterns from the authors' perspective, but I think they are nonetheless valuable to be included. I suggest the authors include the analyses in the Supplemental Information and briefly mention these in the main text. In particular, I'd be very interested in the comparison with previous studies where participants only had access to advice from previous rounds but no payoff information.

Reply: We thank Reviewer 1 for this comment. These analyses were preregistered as secondary analyses because they were either redundant with the preregistered primary analyses reported in the manuscript and/or exploratory. For transparency, we now report all of them in the supplementary materials, and briefly mention them in the main text.

We provide a brief summary of these results here for the reviewer's convenience:

- Agreement among learners did not differ across generations or between treatments. This is consistent with the main-text result showing that participants in both treatments relied on payoff-biased social learning when selecting cultural demonstrators. (line 159)
- Social influence did not differ between treatments when measured as the probability of copying the inherited solution at Trial 1, but it did differ between treatments when measured as 1) the distance between inherited solution and the solution produced at trial 1 and 2) the distance between inherited and transmitted solutions (with the distance being smaller in the high-appeal treatment in both cases). This is consistent with our interpretation that subjective appeal hinders cumulative cultural evolution by reducing exploration. (line 164)
- Individual exploration differed between treatments when measured as the distance between each pair of solutions produced by the same individual (line 177), which is consistent with both our interpretation that subjective appeal hinders cumulative

cultural evolution by reducing exploration and the reported result that the number of unique solutions produced by each participant is lower in the high-appeal treatment.

- Analyses of cultural convergence show that both within-chain and between-chain diversity is lower in the high-appeal treatment (line 183). This is consistent with the reported effect that subjective appeal reduces exploration.
- Data from our previous study, in which participants had access to advice from earlier rounds but no payoff information, show no statistically significant evidence of cumulative improvement in scores across generations in either treatment. This indicates that, in our experimental setting, payoff information is necessary for cumulative cultural evolution to occur, but not sufficient. We now discuss this point in the revised manuscript (lines 272-289)

2) In my opinion, the most interesting (and somewhat surprising) aspect of this paper is that it seems that participants are not paying less attention to payoff information in the high appeal condition (if anything they are paying more attention to payoffs, though this was not statistically significant), but are rather exploring less. I suspect that this result was contrary to the authors expectation as well, as the rationale behind the original hypothesis 1 was (if I understand correctly) that the high appeal of the color matching solution reduces participants' reliance on payoff-based copying. So, it seems that at least in this particular experimental setup, what's being negatively affected was the exploration of the solution space. The implication here is that if the experiment had been run long enough generations, mutant solutions with higher payoffs (regardless of appeal) would be noticed and in theory spread in the population, and what subjective appeal negatively affects is the probability that such a mutant solution is generated as individuals are not exploring the solution space as much. The current manuscript has a short paragraph on cultural evolvability, but I think this point should be emphasized more. There is a very large literature on exploration vs. exploitation tradeoff, for example, that the authors could relate to.

Reply: In our experiment, we did expect the high appeal of the color-matching solution to reduce participants' reliance on payoff-based copying, which is not what we observed. We now explicitly acknowledge this discrepancy and discuss it in detail in the revised manuscript (lines 272-289). We framed this as a primary analysis because cultural evolution experiments that identify selective learning as critical to cumulative culture rarely allow learners to combine content-biased and payoff-biased social learning within the same setting.

Reduced exploration of the solution space was not preregistered as a primary prediction. However, we did anticipate that any reduction in performance in the high-appeal condition would be linked to reduced exploration, as reflected in our preregistered secondary analyses on exploration and cultural convergence.

Regarding the implication that the persistence of payoff-biased social learning should necessarily yield cumulative improvement if the experiment were run for sufficiently many generations, we think this conclusion is not warranted. First, demographic models of cultural evolution show that payoff-biased social learning is not sufficient on its own to support

cumulative cultural evolution. Whether cumulative improvement occurs also depends on other factors, including population size and transmission fidelity.

More importantly, social learning strategies are unlikely to be independent of the informational content learners are exposed to, and should instead depend on the informativeness and redundancy of the available cues. In our data, reduced exploration and higher similarity among solutions in the high-appeal treatment led to lower variation in the advice participants received (see Supplementary Figure A7). This is something that we had not anticipated. Under such conditions where advice is highly redundant, payoffs may become the primary basis for discriminating among demonstrators. Conversely, in a counterfactual scenario where higher-payoff "mutant" solutions emerge and spread, the advice received would likely become progressively more heterogeneous. This would create informational conditions that were not present in our experiment, under which learners' strategies might plausibly differ. We now discuss these points in the revised manuscript (lines 272-289).

The current experimental results show that subjective appeal of inefficient solutions do not prevent efficient ones from being noticed (after their payoffs have been observed), and I wonder if this is generally true, especially in naturalistic settings. In the current setup, the payoff information is highly salient (despite the built-in stochasticity). There might be circumstances where the payoff information is sufficiently vague (e.g., not quantifiable in any straightforward way) and subjective appeals dominate the adoption and transmission of cultural practices. This is also why I'm interested in the comparison with a previous study where payoff information was unavailable, as the authors mention in the preregistration.

Reply: We agree with Reviewer 1 that payoff information is highly salient in our experiment. In our setup, evaluating demonstrators on payoffs is cognitively straightforward (participants compare three numerical scores), whereas comparing advice requires reading and integrating several text messages. This asymmetry likely contributes to the finding that participants relied on payoff information when selecting a cultural demonstrator even in the high-appeal condition (now discussed lines 272-289).

In addition, analyses introduced in the revised manuscript show that advice messages in the high-appeal condition were more homogeneous than in the low-appeal condition. As a result, advice content was less likely to differentiate demonstrators in the high-appeal condition, so that payoffs could have been the primary basis for discriminating among demonstrators in this condition.

Whether strong reliance on payoff information should be expected in more naturalistic settings is an important question. We agree that outside the laboratory, payoff information is often noisier, delayed, or difficult to quantify, which could increase reliance on subjective appeal. Moreover, our experimental design likely artificially limits the extent of variation in how appealing candidate solutions can be. With greater variation in appeal among competing solutions, appeal could plausibly play a larger role. We now discuss these points in the revised manuscript (lines 272-289).

Importantly, we view the observed reliance on payoff-biased social learning as making our experiment conservative with respect to the role of appeal. Even though payoff-biased social

learning strongly promotes cumulative cultural evolution, and participants in the high-appeal treatment likely relied on it to a greater extent than they would in many naturalistic settings, cumulative cultural evolution was still derailed in our experiment.

3) In the experiments, participants transmitted a piece of advice to the next generation. There is no explicit analyses of such advice in the current manuscript, and I wonder if there is a way for the authors to meaningfully inform the readers their basic themes. In particular, I wonder if the advice in the high-appeal condition explicitly verbalized the mix-and-match strategy that discouraged the exploration of solution space.

Reply: We thank R1 for this suggestion. We have added new analyses of transmitted advice Specifically, we (i) quantify how advice differs across treatments in terms of length and semantic similarity, and (ii) summarize the main themes of advice in each treatment (Supplementary Section *Analyses on Written Advice;* Supplementary Table A5; Supplementary Figure A8).

The results confirm that participants in the high-appeal treatment transmitted advice consistent with the transmitted feeding patterns, most often recommending the "mix-and-match" strategy throughout the experiment (see Supplementary Figure A8). Exploratory analyses suggest that receiving "mix-and-match" advice tends to reduce exploration more than receiving other types of advice, although the difference is not significant (see Supplementary Figure A9 and Table A6)

Minor comments

One consideration is that participants' cognitive biases may also have a strong cultural component. For individuals familiar with modern computer games, it is quite reasonable to assume that colors convey meaning and therefore should guide plant-feeding decisions. While I agree that it's likely that humans have some innate "like affects like" intuitions, it seems important to acknowledge the possibility that these tendencies are culturally reinforced—potentially grounded in evolved predispositions but scaffolded and amplified through cultural experience.

Reply: The reviewer makes an important point here. Following their recommendation, we now clarify that "The exposure to cultural products that harness these intuitions, such as video games, may further amplify such causal associations" (line 301).

When referencing classic works, use the original when possible (e.g. Frazer's Golden Bough).

Reply: This change has been made.

Subjective appeal has been described as "content biases" in the cultural evolution literature, see (Henrich & McElreath, 2003). Do the authors consider the phenomenon discussed here to be the same as content bias, or do they view it as distinct in some way?

References Henrich, J., & McElreath, R. (2003). The Evolution of Cultural Evolution. *Evolutionary Anthropology*. https://doi.org/10.1002/evan.10110

Reply: We appreciate this question by the reviewer. We see the cognitive biases that influence people's evaluations of solutions to be examples of the kinds of content biases described by cultural evolutionary scholars. We now explicitly state this in the manuscript (lines 68-69).

Reviewer #2:

Remarks to the Author:

This study examines the impact of visual appeal on the efficient transmission of solutions. Examining how different kinds of information/constraints/payoffs impact cultural transmission is relevant to core questions in cultural evolution. The experiments is well designed to explore the effects of an aspect of visual appeal on cultural transmission, albeit with the limitations of experiments of this kind. The methods are clear with adequate detail.

Reply: We thank Reviewer 2 for this positive assessment of our study.

The data are also interesting, but the paper requires revision for the following reason(s). There is a mismatch between the more narrow and careful framing of the results in some of the narrative of the discussion and the overly general claim that these data may explain the persistence of ineffective cultural technologies-shamanism, bloodletting etc.

I suggest more discussion of perceptual interference-which I think the data speak directly to, and less discussion about massively more complex cultural phenomena like magic. The low vs high appeal framing could mean many things-I suggest a more specific and accurate labeling of the experimental manipulation. I also suggest more reflection on mechanisms of perceptual processing underlying the effects of condition on the efficient transmission of solutions.

Reply: We thank the reviewer for pointing out the mismatch between the framing of the results and the general claim that they can explain practices like shamanism and bloodletting. We also now recognize that "appeal" is much more expansive than our very specific manipulation and that it's important to focus on the mechanism under investigation. To address these concerns, we have made the following changes to the manuscript:

1. We now specify that we are investigating a form of subjective appeal rather than subjective appeal more broadly (see, for example, the final sentence of the abstract, and lines 308-310). In the introduction, we have also edited the sentence laying out our hypothesis so that it now states, "Here, we hypothesize that subjective appeal—in this case, the potential for similarity between interventions and targets— can drive the emergence and persistence of ineffective solutions even in conditions otherwise conducive to cumulative cultural evolution." (lines 80-81)
2. We have expanded the discussion of this particular form of subjective appeal in the introduction so that it now constitutes its own paragraph. Again, we frame the visual correspondence between interventions and targets as one of many potential features that bias people's evaluations of solutions; we write, "Of the features that can bias

evaluations of potential solutions, one of the most widespread seems to be a visual correspondence between an intervention and its target." (lines 72-73)

3. We have substantially revised the paragraph of the discussion that related our experiment to more complex cultural practices like divination and shamanism. This includes the following new material, which seeks to clarify that we do not see our experiment as explaining divination and shamanism but establishing the simpler fact that effective practices can be maintained by forms of subjective appeal:
   "Our results experimentally illustrate that ineffective cultural practices can be maintained by subjective appeal. Our experiment investigated a single form of subjective appeal—visual similarity—and the resulting cultural product was much simpler than practices like divination or shamanism. We suspect that these more complex practices tap into other dimensions of subjective appeal beyond those investigated here. Nevertheless, our study serves as a proof of concept, demonstrating that functional benefits are not required for the emergence and persistence of costly ineffective cultural practices." (lines 307-313)
4. In line with the reviewer's suggestion to reflect more on the mechanisms underlying the appeal of visual similarity, we have added the following sentence to the discussion: "The reasons for such causal biases remain debated; proposals include that people erroneously learn causal relationships from environmental regularities and that the principle that like-affects-like occurs frequently enough in the natural world that it proved a useful scaffold for understanding causality for our ancestors." (lines 293-297). We have also acknowledged that cultural products might further reinforce these intuitions: "The exposure to cultural products that harness these intuitions, such as video games, may further amplify such causal associations." (lines 301-302)

Reviewer #3:

Remarks to the Author:

This manuscript describes and explores an interesting experimental design to investigate the ``law of similarity" found in a variety of anthropological case studies. Using what the authors call subjective appeal, they investigate whether the degree of visual similarity between features of plants and features of nutrients determines the effectiveness of the solutions. They conclude that their experimental design can successfully replicate situations seen in the ``real world" where inefficient but matching solutions persist.

Is this a fair assessment or do the authors propose subjective appeal to be the cognitive bias that generates the mix and match pattern? If so, the cognitive underpinnings of subjective appeal should be better explained so that the contribution of the experiment beyond replicating the "real-world" pattern (which is great!) becomes more obvious.

Reply:

We thank the reviewer for this helpful question. We now recognize that the connection between subjective appeal and the features explored in our particular manipulation (visual correspondence—an example of the law of similarity) was unclear in the original manuscript. We have thus made several changes to clarify that subjective appeal comprises the broader set

of features that influence people's evaluations of solutions by appealing to cognitive biases. Our experiment just tested one form of subjective appeal: the visual correspondence between intervention and target. We have made the following changes to clarify this point:

1. We specify that we are investigating a form of subjective appeal rather than subjective appeal more broadly. See, for example, the final sentence of the abstract, line 308-310 in the discussion, and line 80-81 in the introduction.
2. In the introduction, we now separate the discussion of subjective appeal more broadly (paragraph 3) and the particular form that we are investigating (paragraph 4).
3. We have substantially revised the paragraph in the discussion relating the form of subjective appeal we investigated to more complex cultural practices. We clarify, for example: "Our experiment investigated a single form of subjective appeal—visual similarity—and the resulting cultural product was much simpler than practices like divination or shamanism. We suspect that these more complex practices tap into other dimensions of subjective appeal beyond those investigated here. Nevertheless, our study serves as a proof of concept, demonstrating that functional benefits are not required for the emergence and persistence of costly ineffective cultural practices." (lines 308-313)

I interpreted Figure 1 as proof that the experiment is carefully designed to generate situations where an ineffective but ``matching" solution (similar to the observed resemblance between herbs and body parts) can persist, and the authors are now in the position to explore why this is the case. However, I was left disappointed by this analysis. The experiment has generated such a rich data set, which seems underexplored, apart from looking at the number of unique solutions and the structure of the most common solution.

Reply: We thank Reviewer 3 for raising this point. In the original submission, we intentionally kept the analyses focused on our preregistered primary outcomes and on the key variables needed to test our hypotheses. In the revised manuscript, we substantially expand the analyses to better exploit the dataset. Specifically, we now include additional analyses that: (i) decompose the dimensions contributing to solution efficacy, (ii) quantify social influence using multiple complementary measures, (iii) characterize exploration at both the individual and collective levels, and (iv) analyze the written advice (length, semantic similarity, and content classification).

Regarding the most common solution, the authors say that the most common solution in the low appeal treatment accounted for 3% of the sixth-trial responses. What does this mean? There are 20 chains, and in each chain there are 4 sixth trials. 3% of 80 is 2.4 individuals (should it not be an integer?). So the most common solution has been discovered by 2 individuals? In the high appeal group 16% of 80 (12.8) individuals show the same solution. This is interesting but is this from individuals at the beginning of the chain or at the end? In any case, the authors contrast the most common solution as if they were representative of the different treatments, which they are not — especially not in the low appeal group, given the very low numbers of individuals having discovered them. One needs to quantify the variation in the solutions produced by both treatments possibly along at least three dimensions contributing to the

theoretically most efficient solution: number of plants used, number of blue treatments and number of treatments per plant.

Reply: We thank Reviewer 3 for this constructive comment. Our design comprised 20 transmission chains of four generations, with each generation consisting of three participants, for a total of 240 participants (and 240 corresponding sixth-trial/transmitted solutions) per treatment. In the low-appeal treatment, the most common solution was observed in 7 of 240 sixth-trial solutions (about 3%). In the high-appeal treatment, the most common solution was observed in 38 of 240 sixth-trial solutions (about 16%). To improve readability, the revised manuscript now reports these counts directly in Figure 5. We also added a new figure showing the cumulative proportion of transmitted solutions ranked by frequency in each treatment, to make clear that these solutions are not equally representative across conditions (Figure 4).

Figure 5 is intended to illustrate how the most common solutions differ qualitatively between treatments. However, we agree that the two most common solutions are not representative of the low-appeal treatment. We therefore added additional analyses, as suggested by the reviewer, decomposing transmitted solutions into the number of plants treated, the total number of nutrients used, and the number of blue nutrients used. Consistent with the qualitative patterns highlighted in Figure 5, these analyses show that the number of blue nutrients differs between treatments, with blue nutrients increasing across generations only in the low-appeal treatment. We now report these results in the revised manuscript (lines 149–154).

Further, in Figure 4 net payoff is only the average net payoff as the payoffs are uniformly distributed across the intervals [580,940] and [514,874]. From these uniform distributions, it is also obvious how much the payoff values overlap and that there is a not small probability that the solution labelled as insufficient actually provides a payoff higher than the one of the efficient solutions. Do those large variations in the payoff, in part, drive the result? Can the authors motivate their choice of the payoff function — it seems unusual that the "more right" the solution is (the more plants are included), the higher is the variation, i.e. the harder is it to distinguish between ``inefficient" and ``efficient" solutions based on a very small number of trials.

Reply: We agree with Reviewer 3 that payoffs are highly noisy in our experiment. This was intentional, to capture the fact that in many real-world cultural domains payoffs are only imperfectly diagnostic of the underlying quality of a practice. With lower payoff noise, learners might be able to identify and accumulate efficient solutions more rapidly. We acknowledge the role noise in the introduction (lines 54–55). Importantly, because the payoff-generating process is identical across treatments, payoff noise cannot explain the divergence between treatments and does not affect our central conclusion that high appeal can derail cumulative cultural evolution.

Regarding why noise is implemented at the plant level, the goal was to allow participants to treat each plant as an independent subproblem. We agree that a consequence of plant-level noise is that solutions that treat more plants can exhibit greater variability in total payoff. In practice, however, we observe little variation across participants in the number of plants treated (see Supplementary Figure A1), so differences in payoff variability driven by the number of treated plants are unlikely to drive our results.

The authors state that in both treatments, payoff-biased social learning is used equally. However, what is shown is that the individuals choose the best of the three pieces of information and not whether they indeed use this solution. Can you provide more details on how individuals incorporate the ``learnt" solution in their first try, and so quantify the extent to which social information is used? Currently, the analysis does not tell whether both treatments use social learning to the same extent.

Reply: We thank Reviewer 3 for this constructive comment. We agree that demonstrator choice alone does not establish the extent to which participants implement the selected social information. In the revised manuscript, we therefore added three complementary analyses to quantify social information use: (i) the probability of copying the inherited solution on Trial 1, and (ii) the distance between inherited solution and the solution at the first trial, (iii) the distance between inherited and transmitted solutions.

These analyses show that the probability of copying the received solution at Trial 1 is comparable across treatments, while 1) the distance between inherited solution and the solution produced at trial 1, and 2) the distance between inherited and transmitted solutions differ between treatments (with the distances being smaller in the high-appeal treatment in both cases, line 163).

We note, however, that (1) information from the selected demonstrator remained available throughout all six trials (see screenshots of the experimental interface in the Supplementary Material), giving participants the opportunity to incorporate social information at any point, and (2) these distances reflect both reliance on inherited information and the extent of independent exploration.

Reduced exploration in the high-appeal condition is apparent in multiple analyses (including measures of within-chain and between-chain diversity; see lines 183-184), meaning that these distances are expected to be smaller even if reliance on social information is unchanged.

Moreover, previous experiments show that social information does not result in a dichotomous choice between disregarding and exactly replicating social information (e.g., Derex, M., et al. 2015. Social learning and the replication process: an experimental investigation. Proceedings B), which is consistent with the relatively low probability of replicating the inherited solution in our experiment (see Figure A3.B). Instead, social information tends to channel exploration toward a specific region of the search space (Derex, M., et al. 2025. Social learning preserves both useful and useless theories by canalizing learners' exploration. Proceedings B.). This is now highlighted lines 263-268.

What is the role of the written advice? It may provide interesting information about how individuals reconstruct the payoff function. Why is it not analysed?

Reply: We thank Reviewer 3 for this question. We have added new analyses of transmitted advice. Specifically, we (i) quantify how advice differs across treatments in terms of length and semantic similarity, and (ii) summarize the main themes of advice in each treatment (lines 233–241; Supplementary Section *Analyses on Written Advice;* Supplementary Table A5; Supplementary Figure A8).

The authors state that the low appeal treatments each generation was associated with a 17 point increase but Figure 1 indicates that the mean payoff stays at best constant between generation 3 and 4. Could you clarify? Again, it would be very illuminating if the authors would analyse the diversity in more detail, for exampl,e through exploring development of different parts of the solution within the chains (for example, what do you discover first and when: number of plants, the colour of the nutrient or the number of nutrients per plant). The data set is so rich and provides so much information that can be used to generate a better understanding of why we see what we are seeing. At the moment, the analysis does not do the experiment justice. Also, it would be interesting to see the results of the individual chains in the SI to gain an understanding of the variation within and between the chains.

Reply: The 17-point increase in score is the estimate produced by our statistical model for the parameter Generation. This is now clarified in the revised manuscript (lines 137-140).

We also added additional analyses, as suggested by the reviewer, decomposing transmitted solutions into the number of plants treated, the total number of nutrients used, and the number of blue nutrients used. These analyses show that only the number of blue nutrients differs between treatments, with the use of blue nutrients increasing across generations in the low-appeal treatment. By contrast, the number of plants treated and the total number of nutrients used do not differ between treatments. We now report these results in the revised manuscript (lines 149–154).

Finally, as requested, we added figures showing the results of individual chains for both Score and Cost in the Supplementary Information.

In the conclusion, the authors state that the experiment uncovered the association between visual similarity and functional relationship. In my view, this statement is not supported by the current analysis. If you would take the first individuals in each chain and look at their first solutions. There is no payoff information yet. So if those first solutions are similar to the solutions produced later in the chain, then the functional argument does not hold. The matching of colour may simply be a cognitive attractor (in the absence of any other information), which is hard to overcome, in part due to the high variation in payoff, making it hard to identify a more efficient solution from a small number of trials.

Reply:

We thank the reviewer for raising this point. We now realize that the wording in the original manuscript was confusing; we never meant to imply that the experiment uncovered an association between visual similarity and functional relationship and, in fact, agree that the matching may simply been more akin to a cognitive attractor. We have edited two sentences in the discussion to make this point more clearly:

1. ORIGINAL Such cultural patterns suggest that individuals reliably develop tendencies to associate visual similarity with functional relationships, thereby biasing their assessment of causal relationships.

REVISED: Such cultural patterns suggest that individuals reliably develop tendencies to infer causal relationships from visual similarity.

2. ORIGINAL: In our experiment, the high-appeal treatment implemented conditions likely to engage participants' intuitive tendency to associate visual similarity with functional relationships.

REVISED: In our experiment, the high-appeal treatment created opportunities for visual similarity, which we expected would bias how participants evaluated potential solutions.

REVIEWER COMMENTS

Reviewer #1:

Remarks to the Author:

I appreciate the authors' revision in addression reviewers' concerns. I am satisfied with the revisions as well as the the additional analyses, and recommend acceptance of the manuscript.

Reply: We thank Reviewer 1 for their positive assessment.

Reviewer #2:

Remarks to the Author:

The authors have been responsive to reviewer feedback and the paper is improved as a result.

Reply: We thank Reviewer 2 for their positive assessment.

Reviewer #3:

Remarks to the Author:

I thank the authors for their thoughtful replies to my comments and also for clearing up misunderstandings on my part. I appreciate that they did extra analysis to investigate some of my questions. I find the paper much improved and an interesting contribution to the literature on cumulative cultural evolution.

Reply: We thank Reviewer 3 for their positive assessment.

I am intrigued by some of the new results! Below are additional thoughts that may help strengthen the paper further. Given that we are in round 2, I offer these as suggestions rather than requirements. The authors should prioritise those they find most helpful, given the focus of the paper.

Reply: We thank Reviewer 3 for these constructive suggestions.

I would encourage the authors to have a more nuanced discussion about the social learning claim. You report the percentage of individuals choosing the role model with the best solution with roughly 75% but only 15% are using those solutions unmodified. Now it might be even expected that not the entire solution is copied and Fig. S3 in the SI shows that the differences between the transmitted and first solution are not too small. (Maybe I missed this but what is the solution vector? Is it the vector of 3x9 components describing the number of times red, blue, and yellow is used for every plant?). To make the claim that payoff-biased social learning is used stronger, it would be great to see what the groups actually learn/use from the transmitted solution (they need to learn something to claim social learning). Could you determine where the variation is? For example, does the low-appeal group learn to attend to all 9 plants and use dominantly blue nutrients, and therefore, we see variation mainly in the number of nutrients per plant? This may provide insights into which information is used from the available social information.

Reply: We thank the reviewer for this comment and the helpful suggestions. In terms of social information use, we want to stress that participants do not need to copy the inherited solution at Trial 1, but can implement it at any point during the experiment. When taking subsequent trials into account, the probability of reproducing the inherited solution is 34% in the high-appeal treatment and 21% in the low-appeal treatment. This is substantial given that participants also inherit a piece of advice that can function as a prescriptive rule for how to produce solutions, encouraging implementation of specific features rather than faithful replication of the inherited configuration (e.g., 'The field yields a higher output when you use more blue nutrients').

However, we would like to clarify that our primary claim about payoff-biased social learning concerns demonstrator choice (which corresponds to our preregistered analysis), and not the fidelity with which participants reproduce the inherited configuration. Specifically, we test whether participants use payoff information when selecting among cultural models in a context where they are also exposed to additional informational cues (i.e., written advice) that could, in principle, provide an alternative basis for demonstrator choice. We have revised the phrasing throughout manuscript to make this clearer (lines 127-129; 158; 275-277 & 281-282)

The fidelity with which participants reproduce the inherited configuration is a different question, which is not the target of our main hypothesis. With regard to this point, we do not claim that social information use is stronger in the high-appeal treatment. Although we report that the similarity between the inherited solution and the solution used in the first trial is higher in the high-appeal treatment than in the low-appeal treatment, we explicitly note in the Discussion that these distances reflect both reliance on inherited information and the extent of independent exploration (discussed lines 267–275).

Regarding variation in configurations, we have added additional figures showing how individuals vary the number of plants treated, the number of nutrients used and the proportion of blue, yellow and red nutrients in their configuration (which shows that participants in the low-appeal treatment learn to use dominantly blue nutrients, see Figure A13). We have also clarified how the solution vector is represented (lines 425-435).

I really like Figure A1. Both groups infer that you have to attend to all plants. The low appeal group infers that blue nutrients should be used but both groups struggle with inferring that you should only provide one nutrient per plant. And this may not be too surprising as this fact may not be identifiable due to the large payoff variation. Just a minor point, could you add the error bars?

Reply: We thank the reviewer for this positive assessment of Figure A1. Error bars were included in Figure A1, but they are very small and may therefore be difficult to perceive at first glance, particularly for the number of plants treated and the number of nutrients used (Figure A1.A and A1.C, respectively). This reflects the very low variation observed across trials, generations, and individuals for these variables. To make it clearer, we now added T-shaped error bars for standard errors for each plot that has an error bar. One exception is Figure A7, for which standard errors are extremely small and not meaningfully visible at the scale of the figure.

It's interesting that the most efficient solution has been repeatedly found in the low-appeal group but did not spread. I guess this is expected given the large payoff variation. The advantage of the best solution may only become apparent when this solution is used repeatedly (what indiviudals cannot do). Again, I was wondering whether there is something structurally

similar about the "common" variants shown in Fig. 4, especially in the low-appeal group. For example, do they only differ in the number of nutrients per plant? Then one could say that the low-appeal group did actually discover what they could, given the large payoff variation. I guess the same goes for progress within a generation. Which aspect of the solution are individuals varying or is there no structure to this variation?

Reply: We thank the reviewer for this comment. Although the optimal solution does not spread systematically, participants within chains converge toward solutions with a prevalence of blue nutrients across generations. To illustrate these chain-level dynamics more explicitly, we have added Figure A10 (line 737), which shows, for each generation and treatment condition, the number of transmitted solutions classified as optimal, exclusively blue, or predominantly blue. We have also added additional figures showing, across trials, how individuals vary the number of plants treated, the number of nutrients used and the proportion of blue, yellow and red nutrients in their configuration (Figure A11-13). These figures reveal no systematic pattern in how participants vary the number of plants treated or the number of nutrients used, but confirm that participants in the low-appeal treatment progressively learn to use more blue nutrients and fewer yellow and red nutrients.

line 191: missing table and figure numbers

Reply: Thank you. We have added the missing references and included Table A5, which presents the statistical tests supporting Figure A5.

I still find net-payoff in Fig. 5 misleading. Individuals almost never get this payoff when using this treatment. So either label it average payoff or provide the interval of variation.

Reply: We thank R3 for this suggestion. We have relabeled "net payoff" as "expected score".

This paper presents a pre-registered transmission-chain experiment examining how cognitive appeal can interfere with cumulative cultural evolution. Using a “plant-feeding” task, the authors manipulated whether plants had appealing visual correspondences with nutrient colors (high-appeal condition) or were neutral gray (low-appeal condition). Across four generations, participants in the low-appeal condition progressively converged on efficient, low-cost solutions, while participants in the high-appeal condition developed and culturally transmitted costly but ineffective “mix-and-match” strategies. The results suggest that subjective appeal—stemming from perceptual resemblance—can bias expectations of efficacy, reduce exploratory search, and lead to the persistence of inefficient solutions despite payoff-biased learning.

This is a neat paper that I think would make a contribution to the cumulative cultural evolution literature. I do have a few substantive comments and some specific suggestions which I hope would improve future versions of this manuscript.

1) I was requested by the editor to provide specific comment as to any issues regarding the match between the pre-registrations and the reporting in the manuscript, so I’ll discuss this first. I think the overall matching between the preregistration and the reporting is fine, but there are two notable issues.

- In the pre-registration, the first hypothesis/prediction is that “Participants in the low-appeal treatment are more likely to choose the solution with the highest payoff among the three solutions they can choose from, compared to the high-appeal treatment”. The result as reported in the manuscript show no such difference: “No differences between treatments were observed in terms of social learning strategies. Participants in both treatments relied on payoff-biased social learning to choose from the three available cultural models, selecting the highest-scoring demonstrators 72% of the time in the low-appeal treatment and 78% of the time in the high-appeal treatment (ß= -0.06; 95% CI [-0.16, 0.05]; p = 0.31).” Given that this is a main hypothesis of the study, such discrepancy should be acknowledged and explicitly discussed. I suggest adding a few sentences before the “No difference between treatments were observed…” paragraph. As I’ll elaborate in a later comment, I think the lack of such predicted difference has important implications on the conclusions of the study.
- In the last part of the pre-registration, the authors proposed several additional variables to analyze (copy-pasted here):

  “- Cultural selection: Agreement among learners across generations and between treatments.
  - Social Influence (1): Probability of copying the previous participant's solution at

the first trial across generations and between treatments.
- Social Influence (2): Distance between inherited and transmitted solutions across generations and between treatments (calculated as the sum of the absolute differences between each nutrient for each plant)
- Individual exploration (1): Number of unique solutions across generations and between treatments.
- Individual exploration (2): Distance between each pair of solutions produced by the same individual across generations and between treatments. (calculated as the sum of the absolute differences between each nutrient for each plant)
- Cultural convergence: Within- and Between-chain cultural diversity of transmitted solutions across generations and between treatments.
- We will also compare the data to a previous study, where participants only had access to advice from previous participants before selecting a solution, as opposed to the current study, where they had access to both advice and the payoff from the last trial. ”

Among these, the present manuscript explicitly discussed “Individual exploration (1)”. I assume that most of the other variables did not yield significant and/or interesting patterns from the authors’ perspective, but I think they are nonetheless valuable to be included. I suggest the authors include the analyses in the Supplemental Information and briefly mention these in the main text. In particular, I’d be very interested in the comparison with previous studies where participants only had access to advice from previous rounds but no payoff information.

2) In my opinion, the most interesting (and somewhat surprising) aspect of this paper is that it seems that participants are not paying less attention to payoff information in the high appeal condition (if anything they are paying more attention to payoffs, though this was not statistically significant), but are rather exploring less. I suspect that this result was contrary to the authors expectation as well, as the rationale behind the original hypothesis 1 was (if I understand correctly) that the high appeal of the color matching solution reduces participants’ reliance on payoff-based copying. So, it seems that at least in this particular experimental setup, what’s being negatively affected was the exploration of the solution space. The implication here is that if the experiment had been run long enough generations, mutant solutions with higher payoffs (regardless of appeal) would be noticed and in theory spread in the population, and what subjective appeal negatively affects is the probability that such a mutant solution is generated as individuals are not exploring the solution space as much. The current manuscript has a short paragraph on cultural evolvability, but I think this point should be emphasized

more. There is a very large literature on exploration vs. exploitation tradeoff, for example, that the authors could relate to.

The current experimental results show that subjective appeal of inefficient solutions do not prevent efficient ones from being noticed (after their payoffs have been observed), and I wonder if this is generally true, especially in naturalistic settings. In the current setup, the payoff information is highly salient (despite the built-in stochasticity). There might be circumstances where the payoff information is sufficiently vague (e.g., not quantifiable in any straightforward way) and subjective appeals dominate the adoption and transmission of cultural practices. This is also why I'm interested in the comparison with a previous study where payoff information was unavailable, as the authors mention in the preregistration.

3) In the experiments, participants transmitted a piece of advice to the next generation. There is no explicit analyses of such advice in the current manuscript, and I wonder if there is a way for the authors to meaningfully inform the readers their basic themes. In particular, I wonder if the advice in the high-appeal condition explicitly verbalized the mix-and-match strategy that discouraged the exploration of solution space.

Minor comments

-One consideration is that participants' cognitive biases may also have a strong cultural component. For individuals familiar with modern computer games, it is quite reasonable to assume that colors convey meaning and therefore should guide plant-feeding decisions. While I agree that it's likely that humans have some innate "like affects like" intuitions, it seems important to acknowledge the possibility that these tendencies are culturally reinforced—potentially grounded in evolved predispositions but scaffolded and amplified through cultural experience.

-When referencing classic works, use the original when possible (e.g. Frazer's Golden Bough).

-Subjective appeal has been described as "content biases" in the cultural evolution literature, see (Henrich & McElreath, 2003). Do the authors consider the phenomenon discussed here to be the same as content bias, or do they view it as distinct in some way?

References

Henrich, J., & McElreath, R. (2003). The Evolution of Cultural Evolution. *Evolutionary Anthropology*. https://doi.org/10.1002/evan.10110

Ze Hong
Assistant Professor of Sociology
University of Macau
